# Analysis of Host Jejunum Transcriptome and Associated Microbial Community Structure Variation in Young Calves with Feed-Induced Acidosis

**DOI:** 10.3390/metabo11070414

**Published:** 2021-06-23

**Authors:** Naren Gaowa, Wenli Li, Sonia Gelsinger, Brianna Murphy, Shengli Li

**Affiliations:** 1 College of Animal Science and Technology, China Agricultural University, No.2 Yuanmingyuan West Road, Haidian, Beijing 100193, China; narengaowa@cau.edu.cn; 2 Cell Wall Biology and Utilization Research Unit, US Dairy Forage Research Center, Agricultural Research Service, US Department of Agriculture, 1925 Linden Drive, Madison, WI 53706, USA; bmurphy8@wisc.edu; 3 Department of Dairy Science, University of Wisconsin-Madison, Madison, WI 53706, USA; slgelsinger@gmail.com

**Keywords:** jejunum, acidosis, young calves, transcriptome, meta-transcriptome

## Abstract

Diet-induced acidosis imposes a health risk to young calves. In this study, we aimed to investigate the host jejunum transcriptome changes, along with its microbial community variations, using our established model of feed-induced ruminal acidosis in young calves. Eight bull calves were randomly assigned to two diet treatments beginning at birth (a starch-rich diet, Aci; a control diet, Con). Whole-transcriptome RNA sequencing was performed on the jejunum tissues collected at 17 weeks of age. Ribosomal RNA reads were used for studying microbial community structure variations in the jejunum. A total of 853 differentially expressed genes were identified (402 upregulated and 451 downregulated) between the two groups. The cell cycle and the digestion and absorption of protein in jejunal tissue were affected by acidosis. Compared to the control, genera of *Campylobacter, Burkholderia, Acidaminococcus, Corynebacterium*, and *Olsenella* significantly increased in abundance in the Aci group, while *Lachnoclostridium* and *Ruminococcus* were significantly lower in the Aci group. Expression changes in the *AXL* gene were associated with the abundance variations of a high number of genera in jejunum. Our study provided a snapshot of the transcriptome changes in the jejunum and its associated meta-transcriptome changes in microbial communities in young calves with feed-induced acidosis.

## 1. Introduction

During early development, ruminants go through a suite of gradual and complex morphological changes, enabling them to efficiently digest milk as its primary diet, and then to transition into a plant-based diet. During the weaning transition period, as solid feed intake increases, there must be qualitative changes in nutrient digestion and metabolism pathways [1]. These changes include the development of immune defense mechanisms, adaptation to energy metabolism, and nutrient digestion and absorption [2]. In this context, the early development and maturation of the gastrointestinal tract (GIT) is important because it plays a crucial role in nutrient absorption in addition to its role as the first line of immune defense [3]. In cattle, calf GIT development is affected by different feeding strategies, and such effects have long-lasting impacts on calf performance [4,5,6]. To promote rumen development and help with the early weaning of the calves, consumption of the maximum amount of easily fermentable starter is commonly practiced during the weaning period [7,8]. However, calves fed high-starch diets could produce an excessive amount of short-chain fatty acids (SCFAs). Once the production of SCFAs exceeds ruminal buffering capacity, passage rate, and especially absorption rate, ruminal pH will drop substantially [9], leading to subacute ruminal acidosis (SARA) [10,11], which is one of the most common metabolic diseases in ruminants [12].

So far, the rumen has been the major focus in dairy cattle nutritional physiology research. It is important to recognize that the effects and the conceivable pathogenesis of acidosis and SARA may not be restricted solely to the rumen. Other parts of the GIT may well be affected [13,14,15]. Among them, the lower gut has received very limited attention. Studies of the lower gut in cattle afflicted by SARA will most likely provide critical knowledge in the treatment and prevention of feed-induced SARA. Bridging the stomach and large intestine in mammals, the small intestine serves as the key point for nutrient and mineral absorption. The three distinct regions of the small intestine, the duodenum, jejunum, and ileum work together to complete the nutrient digestion. The jejunum mainly absorbs small nutrient particles digested in the duodenum [16]. The main function of the ileum is to absorb the available nutrients that were not absorbed by the jejunum. The nutrients transported through the epithelial cells of the jejunum and ileum include fructose, vitamins, glucose, amino acids, and small peptides [17,18]. The interior of the jejunum is lined with villi (small finger-like projections), which expand the effective surface area needed for nutrient absorption. As some of the early studies in the immunoglobulin absorption and calf health, El-Nageh proposed that the absorption of colostral proteins occurred predominantly in the jejunum in neonatal calves [19,20]. A recent study by Hammon and co-authors reported that different diets have significant effects on the jejunum mucosal immune system as reflected by their RNA sequencing data [5]. These studies collectively indicated the vital role of jejunum in calf nutrition and health. Despite its important function in nutrient absorption, to our knowledge, there were few reports focusing on the molecular mechanisms underlying jejunum development in young calves and their roles in nutritional metabolic diseases.

Transcriptome studies of the small intestine have provided valuable information in the understanding of the nutritional metabolism and immune responses in piglets [21,22], chickens [23], children [24], and calves [5,25]. In our established model of feed-induced acidosis in young calves, the two diets used in the model were designed to induce or blunt ruminal acidosis. We observed significant physiological differences in the calves receiving each diet, e.g., significantly lower ruminal pH (*p* < 0.01) and reduced dry-matter intake (*p* < 0.04) and body weight (*p* < 0.02) [26]. The host transcriptome profile in rumen epithelial and liver changed significantly in young calves with acidosis induction [27]. Several anti-inflammatory genes were differentially expressed in the liver between the treatment groups. Significant positive correlations between these anti-inflammatory genes and the rumen microbial community were also observed [28]. Our previous work indicated that highly coordinated transcriptome changes may occur in the GIT of young calves inflicted by acidosis. As a follow-up study, the objective of this study was to investigate the transcriptome changes in host jejunum and its associated microbial community in the same group of young calves with nutritionally induced or blunted ruminal acidosis. Whole transcriptome RNA-seq enabled us to conduct a hypothesis-free analysis of gene-expression changes in the jejunum epithelium and any associated changes in the microbial community to delineate the response differences in the jejunum in these young calves with or without feed-induced acidosis. It was expected that calves fed an acidosis inducing diet would exhibit significant changes in the jejunum transcriptome and its microbial communities compared to the ones receiving acidosis blunt diet.

## 2. Results

### 2.1. Acidosis Model Induced by Feed

This work was conducted by using our established acidosis-inducing/blunting model in young calves [26]. Per our previous report, rumen pH was reported in our recently published work [27]. Overall, the mean ruminal pH in the Aci group was significantly lower than the Con group (*p* < 0.01; 5.36 vs. 5.69). Ruminal pH was lower in the Aci group during the week of weaning (*p* < 0.05; 5.00 vs. 5.47), though pH was not different by diets at other weeks. Beginning at week 4, lower starter intake was observed in calves from the Aci group compared with those in the control group (*p* < 0.04). Beginning at week 5 through 16, the body weight of Aci calves was lower than that of Con calves (*p* < 0.02).

### 2.2. Transcriptome Changes in Host Jejunum Tissue

Total RNA sequencing of jejunum tissue generated an average of 46,541,838 ± 667,279 and 46,965,765 ± 320,045 (mean ± SE) reads in Con and Aci groups respectively. After sequence alignment, an average of 96.75% ± 0.44% (for the Con) and 96.98% ± 0.10% (for the Aci) of the reads within each group were mapped to the reference genome. Using the Bioconductor package ssizeRNA [29], we determined that we had sufficient power to identify significantly differentially expressed genes with the RNA-sequencing data we generated (power = 0.90).

#### 2.2.1. Differentially Expressed Genes in Jejunum

**Figure 1:**
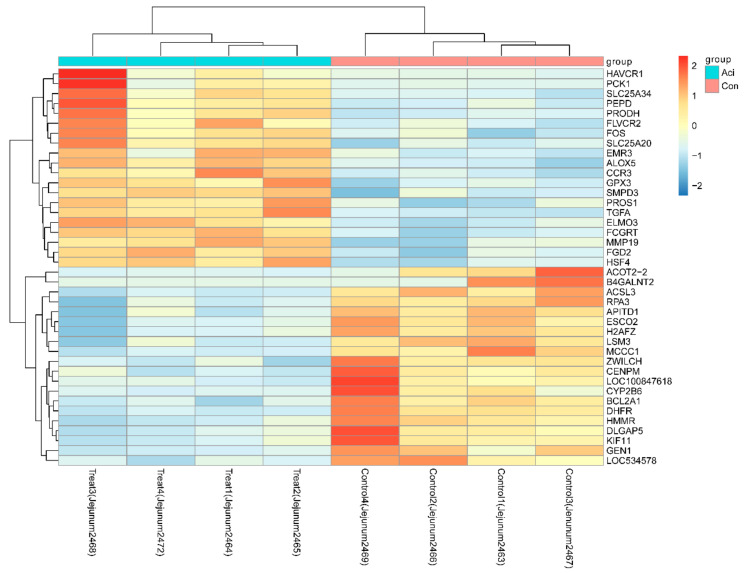
Heatmap showing top 40 DEGs between treatments. The log2 ratio values of DEG abundance were used for cluster analysis with the R pheatmap package. Red and blue indicate relative over- or under-expression of genes, respectively. Aci, feed-induced acidosis group; Con, control group.

**Figure 2:**
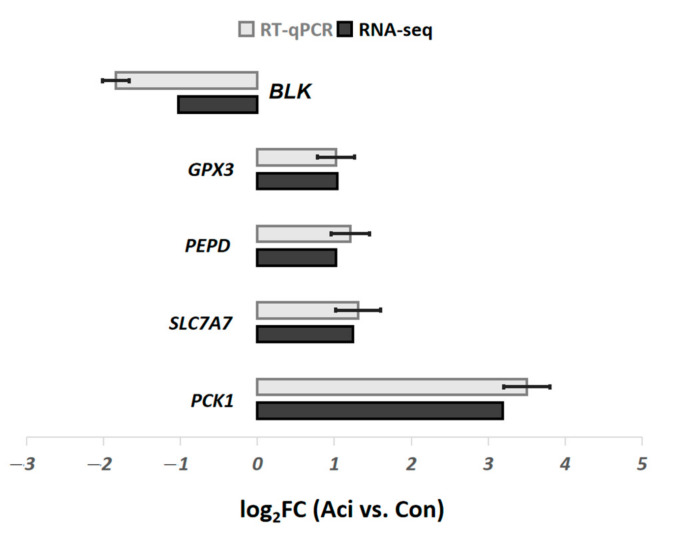
RT-qPCR confirmation of five DEGs identified by RNA-seq.

A total of 853 differentially expressed genes (DEGs) were identified between the Con and Aci groups (*p* < 0.05), with 402 showing increased expression (IEGs) and 451 showing reduced expression (REGs) in the Aci group (Supplementary Materials Table S1). A clear separation between the Aci and Con groups was identified by using the top 40 most significant DEGs (Figure 1). The expression of five target genes was successfully confirmed by using RT-qPCR (Figure 2).

#### 2.2.2. Functional Annotation of DEGs

**Figure 3:**
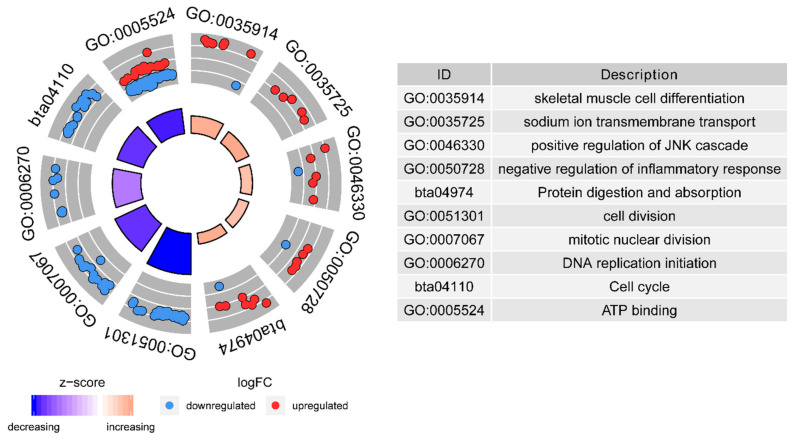
The GO circle of functional enrichment analyses with DEGs. Each segment of the circle shows the scatter plot the log2FC of the enriched genes. The red points display upregulation, and the teal-blue ones display downregulation. The bigger segment size of the inner circle indicated the higher expression of the enriched pathway. The darker the shade of blue means more decreasing. The darker the shade of red means more increasing.

As shown in Figure 3 and Supplementary Materials Table S2, the most noteworthy eight identified GO functions using all of the DEGs were sodium ion transmembrane transport (GO: BP:0035725, five genes, z-score = 2.34), skeletal muscle cell differentiation (GO: BP:0035914, eight genes, z-score = 2.12), negative regulation of inflammatory response (GO: BP:0050728, six genes, z-score = 1.63), positive regulation of JNK cascade (GO: BP:0046330, six genes, z-score = 1.63), cell division (GO: BP:0051301, 24 genes, z-score = −4.90), ATP binding (GO: MF:0005524, 76 genes, z-score = −4.59), mitotic nuclear division (GO: BP:0007067, 18 genes, z-score = −4.24), and DNA replication initiation (GO: BP:0006270, eight genes, z-score = −2.83). Moreover, two KEGG pathways were enriched, including cell cycle (bta04110, 18 genes, z-score = −4.24 and protein digestion/absorption (bta04974, eight genes, z-score = 2.12).

Additionally, the genes enriched in the biological progress of sodium ion transmembrane transport were all IEGs (*SLC6A8*, log_2_FC = 1.25; *SLC24A1*, log_2_FC = 1.03; *SLC4A4*, log_2_FC = 1.24; *SCNN1A*, log_2_FC = 1.17; *ANO6*, log_2_FC = 1.01; *p* < 0.05, Supplementary Materials Table S1). The DEGs enriched in the following KEGG pathways, cell cycle, biological progress of mitotic nuclear division, cell division, and DNA replication initiation were mainly IEGs (only one REG). The genes enriched in the molecular function of ATP binding included both the REGs and IEGs, with a smaller portion of it contributed by REGs.

Our RNA sequencing work identified 28 DEGs encoding the solute carrier group (SLC) of membrane transport proteins. Among these SLC protein-coding genes, 15 DEGs (*SLC15A1, SLC22A15, SLC6A12, SLC3A2, SLC7A10, SLC7A7, SLC25A20, SLC25A34, SLC25A22, SLC9B2, SLC22A5, SLC4A4, SLC51A, SLC1A1,* and *SLC31A1*) were involved in integral component of membrane (GO:0016021, *p* < 0.05, Supplementary Materials Table S3). Five IEGs (*SLC15A1, SLC3A2, SLC7A8, SLC1A1,* and *SLC7A7*) were enriched in KEGG pathway of protein digestion and absorption.

### 2.3. Jejunum Active Microorganisms and Its Association with Jejunum mRNA Expression Changes

**Figure 4:**
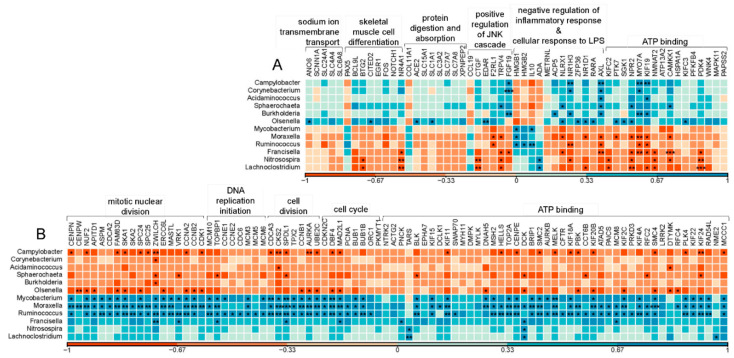
The Pearson correlation matrix between the active bacteria with significant abundance changes and the host jejunum DEGs. The color scale bar below (**A**) and (**B**) show the Pearson *r* value: the more positive the correlation (closer to 1), the darker the shade of blue; the more negative the correlation (closer to −1), the darker the shade of red. (**A**) The IEGs enriched in ATP binding and the DEGs enriched in protein digestion and absorption, sodium ion transmembrane transport, skeletal muscle cell differentiation, negative regulation of inflammatory response, cellular response to LPS, and positive regulation of JNK cascade. (**B**) The REGs enriched in ATP binding and the DEGs enriched in cell cycle, cell division, mitotic nuclear division, and DNA replication initiation; * *p* < 0.05; ** *p* < 0.01; *** *p* < 0.001.

Using bioinformatically extracted rRNA reads, microbial taxonomic classification was achieved by using Kraken, which identified a total of 60 genera in the jejunum (Supplementary Materials Table S4). Kruskal–Wallis test indicated 12 significantly differentially abundant genera in jejunum between Aci and Con groups (Table 1, *p* < 0.05). Among them, three Gram-negative bacteria (*Campylobacter, Burkholderia,* and *Acidaminococcus*) and two Gram-positive bacteria (*Corynebacterium* and *Olsenella*) showed significantly higher abundance in Aci group compared to the Con group. Pearson’s correlation analysis between these 12 genera and DEGs in the jejunum indicated that *Campylobacter, Corynebacterium,* and *Burkholderia* had significantly positive correlation with the four IEGs collectively (*FGF19, NR1H3, AXL*, and *MYO7A*; Figure 4A; *r* > 0.7; *p* < 0.05). GO analysis showed that these genes were involved in the biological progress of positive regulation of c-Jun N-terminal kinases (JNK) cascade, negative regulation of inflammatory response and the molecular function of ATP binding (Figure 4A).

Furthermore, several genera, *Ruminococcus, Mycobacterium, Moraxella, Francisella, Nitrosospira*, and *Lachnoclostridium*, had significantly lower abundance in Aci group compared to the control. Among them, three genera (*Francisella, Nitrosospira*, and *Lachnoclostridium*) were positively related to gene *DCK* and negatively related to five IEGs (*NPR2, NMNAT2, PDK4, NR4A1*, and *TRPV4*; Figure 4A, *r* < −0.7, *p* < 0.05). These genes showed an enrichment in several biological processes, including positive regulation of JNK cascade, negative regulation of inflammatory response and molecular function of ATP binding. *Moraxella*, *Ruminococcus*, and *Mycobacterium* were associated with more than half of the REGs, which were enriched in the first five decreased functions, as shown in Figure 4B (*r* > 0.7, *p* < 0.05).

## 3. Discussion

### 3.1. Jejunum Remodeling at the Transcriptome Level Caused by Acidosis-Induced Feed

In our study, the impact of feed-induced acidosis was reflected by the enrichment of DEGs in cell division, DNA replication initiation, ATP binding, cell cycle, and mitotic nuclear division. These findings implied that ruminal acidosis inflicted cellular reproduction and development in the jejunum at the transcriptome level. The genes classified as downstream regulators and relevant to the cell division, DNA replication initiation, cell cycle, and mitotic nuclear division showed coordinated negative activation z-scores (Figure 3). Among them, the cyclin-dependent kinase 1 (*CDK1*) gene was shared by these biological functions and was a REG in rumen tissue (log_2_FC = −0.78; *p* < 0.05), as shown in our previous study [27]. CDK1 is a highly conserved protein acting as a serine/threonine kinase and it has a key role in cell-cycle regulation [30]. However, little is known about its function in cattle, specifically in the context of ruminal acidosis.

**Figure 5:**
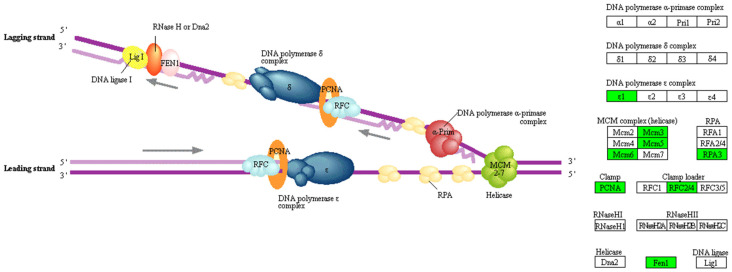
DNA replication of jejunum epithelial cell. This figure is based on the pathway bta03030 shown in KEGG [42]. Green markers indicate that proteins encoded by genes with reduced expression levels in treated calves. The graph was produced by “pathview” in R.

As a systematic and complex process, DNA replication ensures the faithful duplication of the genome as required by the cell division at each cell cycle [31]. Of note, as shown in Figure 5, nine DEGs enriched in DNA replication were significantly downregulated (*RFC4*, log_2_FC = −0.56; *RFC2*, log_2_FC = −0.59; *POLE*, log_2_FC = −0.66; *PCNA*, log_2_FC = −0.78; *MCM3*, log_2_FC = −0.67; *MCM5*, log_2_FC = −0.51; *FEN1*, log_2_FC = −0.55; *MCM6*, log_2_FC = −0.70; *RPA3*, log_2_FC = −0.82; *p* < 0.05, Supplementary Materials Table S1). Mini-chromosome maintenance proteins (MCMs) are highly conserved proteins, involved in the initiation of eukaryotic genome replication [32]. These proteins are subunits of a larger protein complex, including MCM2-7 [33]. Within MCM2-7, six MCMs are loaded onto G1-phase DNA in a bicyclic heterohexameric structure (with the arrangement of MCM2-MCM6-MCM4-MCM7-MCM3-MCM5 in counterclockwise order) [34]. Most MCM2-7 hexamers remain dormant on chromatin [33]. They can be activated when the replication fork stalls or slows down. This feature is critical for maintaining genome integrity and stability in the event of DNA damage and replication stress [35,36]. In cattle, the direct causal relationship between MCM5 variants and calf growth has not been reported. However, it has been suggested that growth defects are associated with decreased MCM5 expression in zebrafish [37]. In our study, lower body weight in calves in the Aci group might be associated with the decreased *MCM5* expression in jejunum and liver tissues [28]. However, the Aci group also consumed significantly less starter feed. Though we were not able to make definitive conclusions about the potential causal relationships between reduced weight gain and decreased expression in *MCM5*, future studies are needed to further investigate the potential association between *MCM5* expression and reduced body weight. Notably, MCM2-7 genes were all downregulated in Aci group although *MCM2, MCM4,* and *MCM7* were not identified as the REGs (*MCM2*, log_2_FC = −0.40; *p* = 0.058; *MCM4*, log_2_FC = −0.43; *p* = 0.052; *MCM7*, log_2_FC = −0.46; *p* = 0.038). Thus, it is possible that early age ruminal acidosis could potentially affect not only the overall body growth but also organ development. Acting as a DNA clamp, proliferating cell nuclear antigen (PCNA) is a synthesis factor for DNA polymerase δ in eukaryotic cells [38]. DNA polymerase epsilon is involved in the resynthesis of damaged DNA strands during DNA repair. Thus, *PCNA* is essential to both DNA synthesis and DNA repair [39]. Additionally, it is a common marker to assess cell apoptosis and proliferation in the jejunum as reported in many studies [40,41]. As shown in the study by Greeff et al. [40], the expression of *PCNA* is positively related to mucosa length of jejunum in the piglet. The reduced *PCNA* expression in the Aci group may suggest that early age rumen acidosis could affect jejunum tissue growth. Further targeted, follow-up studies are warranted.

Two other REGs identified in this study, *MASTL* and *AURKA* (log_2_FC = −0.60, and log_2_FC = −0.52, respectively; *p* < 0.05, Supplementary Materials Table S1) were also downregulated in the liver (log_2_FC = −1.63, and log_2_FC = −1.35, respectively; FDR < 0.05) of young calves in the Aci group [28]. *MASTL* gene encodes for the protein microtubule-associated serine/threonine kinase, which is a key regulator of mitosis, ensuring the proper maintenance of mitotic substrate phosphorylation [43]. As reported before, MASTL kinase could enhance the CDK1 mitotic phosphorylation events during mitosis [44]. On the other hand, CDK1 inactivation reduced its ability in phosphorylation inhabitation, which is followed by a decline in MASTL activity [45,46,47]. Thus, mitotic phosphorylation might decline during mitosis in the jejunum tissue of young calves with ruminal acidosis. Another important regulator of cell-cycle events is Aurora kinases (AURKs), which are conserved serine/threonine kinases with an important role in regulating cell-cycle events [48]. As one of these AURKs, the *AURKA* gene encodes aurora kinase A, known as serine/threonine-protein kinase 6. Located at the spindle poles, AURKA is closely associated with chromosome segregation [49] and it regulates cytokinesis by regulating centrosome maturation [50]. Aurora B kinase, encoded by *AURKB* gene, plays a role in the attachment of the mitotic spindle to the centromere. As a REG in our study (log_2_FC = −0.69, *p* < 0.05, Supplementary Materials Table S1), *AURKB* was involved in the biological progress of ATP binding (Figure 4B). Interestingly, Zhu and co-authors’ suggested that AURKA and AURKB were instrumental in affecting intestinal microbiome homeostasis, which was essential for intestinal health [51]. In our study, we found that *AURKA* was negatively related to two Gram-positive bacteria (*Corynebacterium* and *Olsenella*, Figure 4B, *r* < −0.7, *p* < 0.05) while *AURKB* was positively related to *Ruminococcus* and *Mycobacterium* (Figure 4B, *r* > 0.7, *p* < 0.05). Taken together, our findings suggested that acidosis-inducing diet may affect cellular growth of jejunum tissue at the transcriptome level and the expression changes in *AURK*s might be related to jejunal microbiome homeostasis.

### 3.2. Significant Expression Changes in Nutrient Transporters and Sodium Ion Transmembrane Transport in the Jejunum of Acidotic Calves

**Figure 6:**
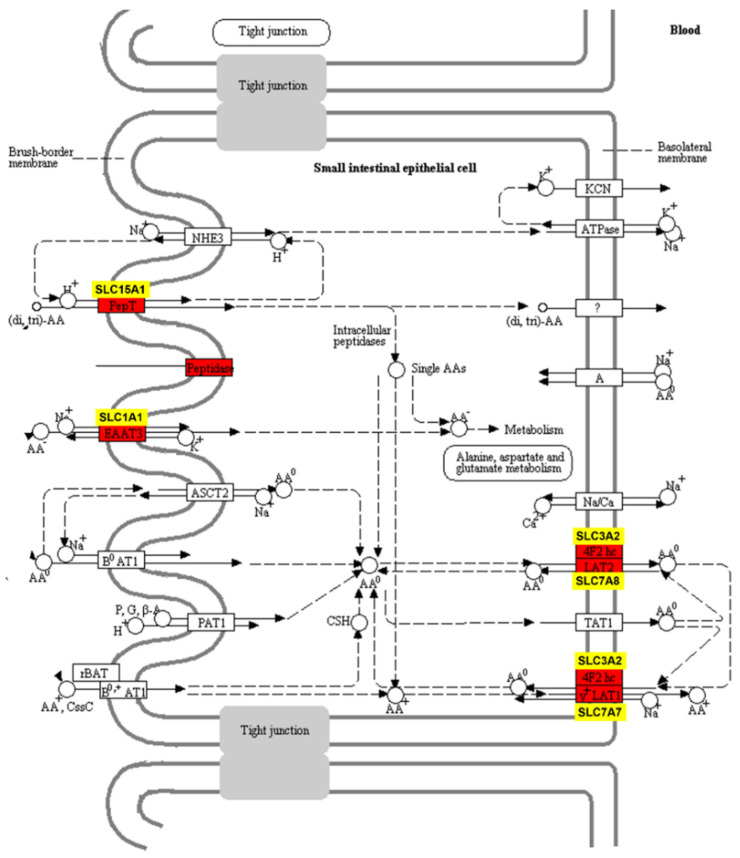
Amino acids and peptides transport in jejunum epithelial cell. Red marker: transporters encoded by the IEGs. Yellow marker: IEGs encode the transporters for anionic amino acid (AA), peptides, neutral AA. This figure is based on the pathway bta04974 shown in KEGG [42]. Red markers were printed by “pathview” in R.

The solute carrier (SLC) group of membrane transport proteins included more than 400 members that were categorized into 65 families. Most of these proteins located in the cell membrane [52]. *SLC1A1, SLC15A1, SLC7A7*, and *SLC7A8* have been identified as the major intestinal transporters for anionic amino acid (AA), peptides, and neutral AA. Their elevated expression in the acidotic calves might be promoted by the acidosis-inducing diet [53,54]. As shown in Figure 6, genes *SLC1A1* and *SLC7A7* encode AA transporter excitatory amino acid transporter 3 (EAAT3) and Y + L amino acid transporter 1 (Y^+^ LAT1) respectively. These two AA transporters (EAAT3 and Y^+^ LAT1) belong to the Na^+^-dependent systems of AA transporters [55]. *SLC7A7* was reported with high transport efficiency of Methionine in the jejunum in growing goats [56] and has been shown to interact with solute carrier family 3 member 2 (*SLC3A2*) [57]. *SLC7A8* gene encodes LAT2, which is an energy-independent exchanger of neutral amino acids [58]. *SLC3A2*, encoding the 4F2 cell-surface antigen heavy chain, was upregulated in the Aci group and was consistent with the expression change of *SLC7A7* gene in this study. Meanwhile, according to Liao’s study, increasing ruminal supplementation of starch hydrolysate could improve the supply of microbe-derived AA in the jejunum [59]. Thus, in our study, the IEGs of AA transporters may respond to increased AA in jejunum lumen because of the higher concentration of starch in the Aci group. Alternatively, the Aci calves consumed less starter, which leads to reduced microbial protein. In turn, jejunal mucosa may respond to the lack of protein by elevated gene expression of amino acid transporters. *SLC15A1* gene encodes the peptide transporter 1 (PEPT1), which transports dipeptide and tripeptide located in the brush border membrane (BBM) of enterocytes in the small intestine [60]. PEPT1 plays an important role in the mammalian neonate [61] and is highly expressed in the duodenum, jejunum, and ileum [62,63]. Collectively, these results suggested that the direct response to increased nutrient availability might be the increased expression levels of nutrient transporters in jejunal enterocytes.

Moreover, solute carrier family 6 member 8 (*SLC6A8*) encodes the creatine transporter 1, a member of the superfamily of Na^+^, Cl^−^ coupled transporters for neurotransmitters [64,65] and organic osmolytes [66]. The expression of this gene has been reported in a wide variety of tissues, such as brain, heart, retina, small intestine, kidney, and skeletal muscle [67,68]. *SLC4A4* gene encodes the electrogenic sodium bicarbonate cotransporter 1, which is important for a transepithelial HCO_3_^–^ transport in the intestine of fishes [69,70]. However, very limited work was performed on these genes in cattle. It will be worthwhile to determine the function of SLC group genes in the small intestine of young cattle. Nonetheless, our findings provided evidence that sodium ion transmembrane transporters were an important class of genes significantly impacted by the acidosis-inducing diet in the jejunum.

### 3.3. Immune Response in Host Jejunum Tissue of Young Calves Treated with Acidosis-Inducing Diet

The investigation in jejunum could help better understand the immune responses in neonatal cattle fed a highly fermentable diet [71,72]. Our work indicated that the expression of immunity-related genes in the jejunum was highly responsive to ruminal acidosis. Notably, in the context of the jejunal immune response, NOD-like receptor X1 (*NLRX1*) and nuclear receptor subfamily 1, group H, member 3 (*NR1H3*) were among the IEGs (log_2_FC = −0.60, and log_2_FC = −0.52, respectively; *p* < 0.05, Supplementary Materials Table S1). *NLRX1* gene plays a pivotal in host immunity when it comes to bacterial infections [73]. The expression level of *NLRX1* may be regulated by early, negative feedback circuitry induced by infection [73,74]. In this study, we observed that the expression of *NLRX1* gene was negatively correlated with two pathogenic bacterial genera (*Francisella* and *Moraxella*, Figure 4A, *r* < −0.7, *p* < 0.05). *NR1H3* gene encodes Liver X receptor alpha (LXR-α). As a nuclear receptor [75], *NR1H3* was previously reported as a potentially important transcription regulator of milk fat synthesis [76]. Future studies are needed to fully understand the importance of the *NR1H3* gene function in jejunum after treatment of acidosis-inducing diet. *AXL* is a suppressor of the innate immune response [77] and was upregulated in the Aci group (log_2_FC = 0.52, *p* < 0.05, Supplementary Materials Table S1). In normal tissues, as reported by Rothlin et al. [78], activated *AXL* attenuated the TLR-dependent inflammatory response and natural killer cell activity. Furthermore, *AXL* gene showed significant association with a large number of genera in the jejunum (Figure 4A). Thus, it is evident that early age ruminal acidosis in cattle could affect *AXL* gene expression in the jejunum tissue, and such change is accompanied by coordinated changes in the bacterial community. Interestingly, we did not observe any clinical signs pertaining to pathological infection; for example, there were no significant changes in body temperature between the Aci and control calves. These observations likely depicted a picture of acting immune machinery in the calves treated with high-starch diet. Further follow-up studies are needed to investigate genes involved in inflammation response.

### 3.4. Active Jejunal Microbes and Its Association with Host mRNA Expression Changes

In previously published studies, the abundance of *Campylobacter* and *Olsenella* was higher and the abundance of *Ruminococcus* was lower in acidotic group [79,80,81,82]. Using an RNA-sequencing based approach, we also observed consistent profiles in both the rumen [27] and jejunum (Table 1). *Campylobacters* are generally considered as commensal bacteria within the cattle GIT [79,81,83]. They catabolize glucose [84,85] and play an important role in the nitrogen cycle in the GIT [86]. However, some *Campylobacter* species can infect humans and animals and cause diseases [87]. Among them, *Campylobacter fetus* was reported as a contributing factor to spontaneous abortions in cattle and sheep [88]. Further work is needed to determine whether the abundance changes in the *Campylobacter* genus could damage the functionality of jejunum with the presence of ruminal acidosis. *Olsenella* can ferment carbohydrates into lactic acid. *Olsenella* spp. has also been found to break down glucose in the rumen, owing to its beta-glucosidase capacity [89]. As shown in Kraatz et al.’s study, dietary starch increased the abundance of rumen *Olsenella* [89]. Thus, the increased abundance of the *Olsenella* in jejunum might be the result of the higher concentration of starch in Aci group. *Ruminococcus* is a bacterial genus in the phylum *Firmicutes* and has been found in the jejunum in many studies [28,90,91]. *Ruminococcus* could generate energy through carbohydrate fermentation [92] and produce carbohydrate-active enzymes, such as cellulases and hemicelluloses [93,94]. In this study, *Ruminococcus* was significantly lower in Con group. This might be due to the lower starch and more fiber in the Con group. The increased abundance of *Ruminococcus* in the Aci group may indicate that carbohydrate metabolism is active. This is consistent with the findings in Jami et al.’s study, where the abundance of *Ruminococcus* decreased with increased fiber content in feeds [95]. *Ruminococcus* may also participate in amino acid metabolism [28]. In Li’s work [28], *Ruminococcus* was positively correlated with the biosynthesis of tyrosine, phenylalanine, and tryptophan biosynthesis, and positively involved in methionine and cysteine metabolisms. However, we did not identify any significant association between *Ruminococcus* and jejunum mRNAs that were enriched in protein digestion and absorption. On the other hand, as shown in Wang’s study, the overgrowth of *Ruminococcus* in the jejunum may be detrimental to the host’s ability in nitrogen digestion and utilization [91].

Chemical digestion is considered as the main form of digestion in the jejunum due to its limited number of microorganisms [91]. However, microorganisms can assist to some extent in the digestion of nutrients in the jejunum [96]. In our study, the abundance of *Acidaminococcus* and *Corynebacterium*, which can digest the amino acids [97,98], was significantly higher in Aci group compared to the Con group (Table 1, *p* < 0.05). *Acidaminococcus* could use amino acids and peptides [97] and are amino acid fermenting bacteria found in the rumen [99]. In Myer’s study [100], increased abundance of *Acidaminococcus* was identified in the high feed-efficiency steer, suggesting that the presence of *Acidaminococcus* in jejunal digesta may be indicative of greater GIT proteolytic activity for high-efficiency animals. *Corynebacterium* is a predominant genus in the small intestine of non-ruminants, such as pigs [101], rats [102], and broiler chickens [103,104]. Higher abundance of this genus was found in the small intestine than it is in both the stomach and the large intestine in growing/finishing pigs [101]. Due to its ability to digest the amino acids in the ileum of growing pigs, this genus is a new protein source in growing pigs [98]. So far limited studies investigated the *Corynebacterium* and associated function in the jejunum of cattle. This genus may represent a new avenue for functional microbial ecology in the cattle GIT. *Burkholderia* is a genus of *Proteobacteria*, with most species in this genus being pathogenic [105]. This genus has higher abundance in the Aci group while the abundance of three other genera (*Mycobacterium, Francisella,* and *Moraxella*) was lower in Aci group. It is hard to draw conclusion whether substantial abundance changes in these genera might be deleterious in the jejunum in Aci group. However, it is fair to conclude that ruminal acidosis in calves was accompanied with dramatic abundance changes of dominant bacterial groups in the jejunum, and the major causal factor of such changes lies in the diet composition.

## 4. Materials and Methods

### 4.1. Acidosis Model Induced by Feed Remodeling

Calves included in this study were part of a larger study that was published [26,27,28]. Throughout the experiment, all animal protocols (A005848) were approved by the Animal Care and Use Committee at the University of Wisconsin–Madison. All the procedures relating to animal care and use in this study were implemented in accordance with the guidelines and regulations by the US Dairy Forage Research Center Farm.

In brief, eight Holstein bull calves were enrolled for this experiment. Rumen acidosis was induced by a starch-rich, low-fiber diet (Aci; pelleted, 42.7% starch, 15.1% neutral detergent fiber (NDF), and 5.56% sugar), while texturized starter was fed as a control (Con, 35.3% starch, 25.3% NDF, and 6.17% sugar). The trial started at 1 week of age through 16 weeks with 4 calves randomly assigned to each treatment. At 3 weeks of age, soft rubber cannulas (28 mm i.d.) were fitted to each calf following the method by Kristensen et al. [106]. Before incising the rumen and subsequent cannula insertion, an initial incision was created in the skin and the rumen tissue was sewn to the skin and to heal for 5 days. Between 7 and 9 weeks of age, larger soft rubber cannulas (51 mm i.d.; Bar-Diamond Inc., Parma, ID, USA) were put into place to replace the original cannulas due to accommodate the increase in the size of the fistula. A calibrated pH electrode was inserted into the rumen before each collection to measure rumen pH. Ruminal pH was tested at seven time-points (−8, −4, 0, 2, 4, 8, and 12 h relative to grain feeding) in a single day every other week from week 6 to week 16. Until 17 weeks of age, the 4 calves in each group were sacrificed and jejunum tissues were harvested. Upon tissue collection, they were rinsed with PBS and cut into 4–5 mm^2^ pieces, using sterilized scalpels and placed into Eppendorf safe-lock tubes (Eppendorf North America, Hauppauge, NY, USA). Collected tissues were immediately frozen in liquid nitrogen and stored at −80 °C for further RNA extraction.

### 4.2. RNA Sequencing, Bioinformatics, and Statistical Analysis

**Figure 7:**
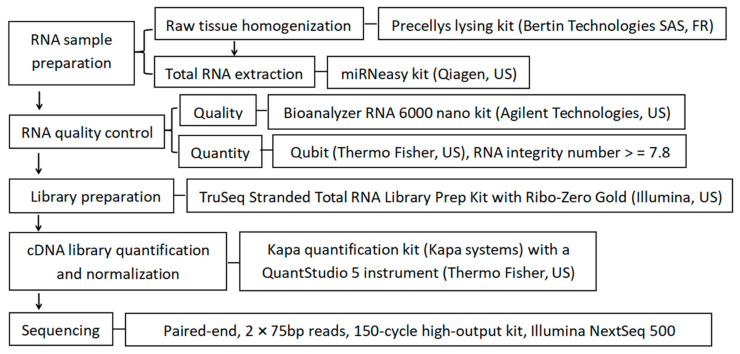
Workflow of RNA library preparation and sequencing.

**Figure 8:**
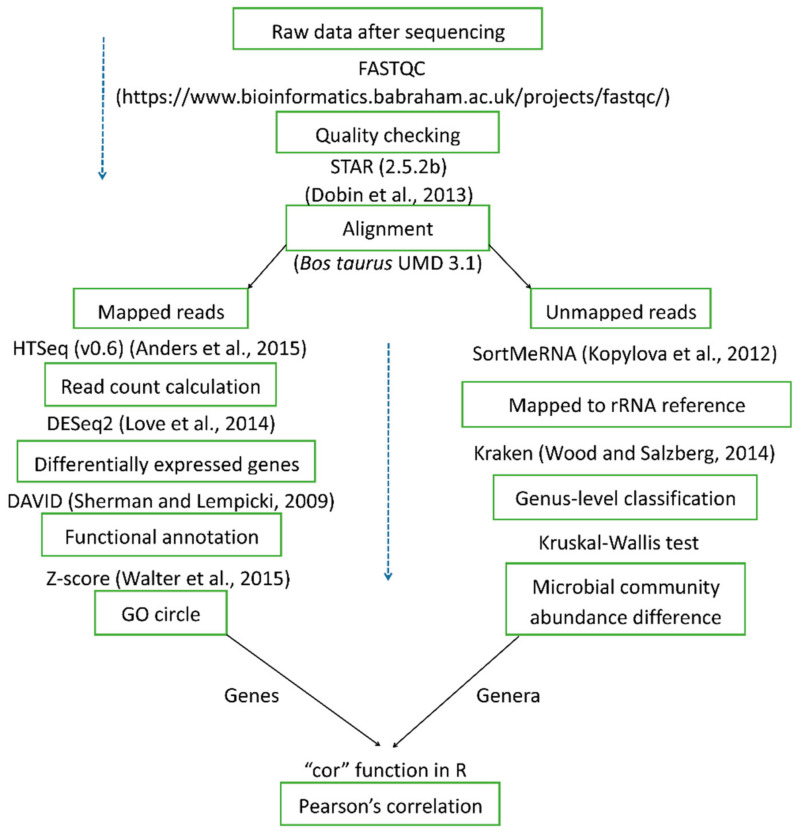
Bioinformatics workflow of whole-transcriptome sequencing data analysis.

RNA library preparation and sequencing following the detailed procedure described in the co-author’s publication [27]. The main steps are shown in Figure 7. Detailed bioinformatics and data analysis steps following the procedures described in the co-author’s publication [27,28] are shown in Figure 8. Briefly, RNA sequencing raw reads were mapped to the cattle reference genome (UMD 3.1) by using STAR (2.5.2b) [107]. Gene-level reads counts were calculated by using HTseq (v0.6) [108]. Differential gene expression analysis was performed by using DESeq2 [109]. Gene ontology and pathway analysis were performed by using DAVID [110]. Cattle genome unmapped reads were considered to be of microbial origin. SortMeRNA (version, 2.1b) [111] was used to enrich rRNA reads from the host genome unmapped reads, using the reference rRNA databases provided by Silva (release 119) [112] and Rfam 11.0 [113]. The enriched rRNA reads were used for bacterial taxonomic classification, using Kraken2 (v.2.0.8-beta) [114]. Raw-read counts at each taxonomic level (i.e., phylum, genus, and species) as identified by Kraken2 were normalized by total number of classified reads per sample by following these steps: (1) the total number of reads mapped to the given taxonomic level (i.e., phylum, genus, and species) was divided by 1,000,000 to obtain the “per million factor”; (2) the total number of reads mapped to each specific given taxonomic level was divided by the “per million factor” to yield the normalized read count. Normalized read counts at a given taxonomic level was used as the measurement for microbial abundance analysis. Pearson’s *r* correlation analysis between the gene expression and microbial genus level abundance was performed by using the cor function in R. The cutoff values of |*r*| > 0.7 and *p* < 0.05 were used to determine significant correlations. Raw read counts for each gene were deposited in FigShare (10.6084/m9.figshare.12845963). Raw rRNA reads were uploaded in NCBI, with the project number of PRJNA647364.

### 4.3. RT-qPCR Analysis

A collection of five randomly selected DEGs was pursued for expression analysis, using RT-qPCR. They included *PCK1, SLC7A7, PEPD, GPX3,* and *BLK*. *PCK1* (phosphoenolpyruvate carboxykinase 1) has a reported role in regulating cell-cycle progression [115]. *SLC7A7* provides instructions an amino acid transporter, which is involved in certain building blocks of protein [116]. *PEPD* (peptidase D) provides instruction for making prolidase, which is involved in the finals steps of breaking down some proteins obtained through dies [117]. *GPX3* (Glutathione peroxidase 3), as a member of glutathione peroxidase family, functions in the detoxification of hydrogen peroxide [118]. *BLK* (Tyrosine-protein kinase) is typically involved in cell proliferation and differentiation [119]. The protein encoded by *BLK* has been reported in stimulating insulin synthesis in response to glucose [120]. *PAX5 encodes* a member of the paired box transcription factor family. Paired box transcription factors are important regulators during early development. Encoding a B-cell lineage specific activator protein, this gene expresses at early stages of B-cell differentiation [121].

We performed cDNA synthesis by using High-Capacity cDNA synthesis mix (Thermo Fisher Scientific, Waltham, MA, USA) with 2 μg of total RNA. Gene-specific, Taqman assay probes were ordered from Thermo Fisher’s probe assay collection (Thermo Fisher Scientific, Waltham, MA, USA) as follows: Bt03224295_m1 (*PCK1*), Bt03244891_m1 (*SLC7A7*), Bt00944663_m1 (*PEPD*), Bt03259219_m1 (*GPX3*), Bt03248098_m1 (*BLK*). QuantStudio 5 fast system (Thermo Fisher Scientific, Waltham, MA, USA) performed all qPCR reactions. The following PCR condition was used: one step of uracil-N-glycosylase (UNG) [122] treatment at 50 °C for 2 min, followed by an initial denaturation/activation step at 95 °C for 10 min, and then 40 cycles at 95 °C for 15 s and 60 °C for 60 s. The fold change in gene expression was obtained following normalization to two reference genes, Beta-actin (*ACTB*) [123] and hydroxymethylbilane synthase (*HMBS*) [124]. These two reference genes were used as an endogenous reference gene for all reactions. Final gene-expression data were obtained by using the 2^−ΔΔCt^ method [125].

## 5. Conclusions

Our study painted the picture of active transcriptome changes in the jejunum of young calves fed an acidosis-inducing diet. We observed transcriptomic remodeling in the jejunum, as reflected by the downregulation of *MCMs* and *PCNA*, which were involved in DNA replication. Moreover, genes encoding transporters of amino acid and peptide, and sodium ion transmembrane transport changed significantly in the jejunum as a response to acidosis-inducing diet. Of note, our study found significant abundance changes in active jejunal microbes. Several of the microbes showed significant association with many host genes involved in the immune response, such as the upregulated *AXL* and *NLRX1*. In summary, our study provided transcriptome-level knowledge of the jejunum functionality in young calves subjected to acidosis-inducing diet. This study was performed at only one time-point (17 weeks of age) with one tissue type. A comprehensive study that encompasses the entire weaning period and covers a wider range of GIT tissues would undoubtedly provide insightful knowledge regarding the dynamics between the host transcriptome and the associated microbial community. Additionally, follow-up research is needed to determine whether calves that experience ruminal acidosis are more susceptible to diseases later in life.

## Figures and Tables

**Table 1:** The active bacteria changes in jejunum when using rRNA transcriptome analysis.

Genus	Normalized Read Counts (Mean ± SEM)		*p*-Value	Description
	Con	Aci		
*Campylobacter*	738.22 ± 208.78	3581.05 ± 1312.38	0.021	Gram-negative
*Corynebacterium*	1096.51 ± 181.40	2828.44 ± 1278.40	0.021	Gram-positive
*Acidaminococcus*	658.54 ± 88.52	2119.04 ± 1371.72	0.021	Gram-negative
*Olsenella*	484.36 ± 174.05	1521.03 ± 908.39	0.043	Gram-positive
*Sphaerochaeta*	206.85 ± 44.44	520.41 ± 172.81	0.043	--
*Burkholderia*	0.00	129.69 ± 116.15	0.047	Gram-negative
*Moraxella*	370.53 ± 64.52	60.63 ± 37.34	0.020	Gram-negative
*Francisella*	3510.84 ± 200.53	1768.27 ± 361.60	0.021	Gram-negative
*Nitrosospira*	4510.05 ± 690.55	1783.64 ± 546.93	0.021	--
*Lachnoclostridium*	5588.09 ± 1076.92	1553.04 ± 356.10	0.021	Butyrate producer
*Mycobacterium*	176.47 ± 55.11	22.34 ± 22.34	0.038	Cannot be stained
*Ruminococcus*	1847.28 ± 380.77	546.59 ± 212.98	0.043	Gram-positive

## Data Availability

The raw read counts for each gene were deposited in FigShare (10.6084/m9.figshare.12845963). Raw rRNA reads were uploaded in NCBI, with the project number of PRJNA647364.

## Supplementary Materials

The following are available online at https://www.mdpi.com/article/10.3390/metabo11070414/s1. Table S1: Differentially expressed genes between Aci and Con groups, Table S2: Functional annotation of DEGs with z-score, Table S3: Functional annotation for differentially expressed SLC protein-coding genes between Aci and Con groups, Table S4: Read counts of genera in the jejunum between Aci and Con groups.
