# Supplementary material for: Analysis of Host Jejunum Transcriptome and Associated Microbial Community Structure Variation in Young Calves with Feed-Induced Acidosis
Source: Metabolites. 2021 Jun 23;11(7):414. doi: 10.3390/metabo11070414 (PMC8303401; doi:10.3390/metabo11070414)
Supplement: Supplementary file 1 [file metabolites-11-00414-s001.zip › Supplemental Table S1-DEGs.pdf]

Supplemental Table S1 Differentially expressed genes between Aci and Con groups

ID	baseMean	log_2FoldChange	*P*-value
PCK1	2296.15	3.19	< 0.001
MAB21L3	51.29	3.07	< 0.001
SLC1A1	1544.57	2.91	0.002
TMEM37	271.23	2.70	0.004
SUSD2	1389.33	2.68	0.005
FGF19	144.29	2.56	0.041
PAPSS2	769.40	2.51	0.007
LOC536516	61.89	2.41	0.003
SLC7A8	908.05	2.26	0.022
AQP7	203.82	2.24	0.009
CYP4F2	547.35	2.23	0.030
LOC100849069	30.16	2.20	< 0.001
HAVCR1	975.04	2.19	< 0.001
VNN1	1319.25	2.15	0.003
LOC101905715	38.68	2.15	0.034
CCL24	329.32	2.14	< 0.001
CAPG	535.36	2.06	0.007
ASB4	26.22	1.92	< 0.001
XPNPEP2	968.08	1.91	0.001
LOC614741	136.59	1.85	< 0.001
SLC15A1	4464.69	1.82	0.015
M-SAA3.2	99.06	1.80	0.002
SLC26A6	338.06	1.78	0.001
SERPINA1	84.28	1.78	0.026
FUT1	185.63	1.72	0.004
SLC23A1	519.58	1.67	0.041
AHCYL2	4652.87	1.66	0.002
MGC152010	111.05	1.63	0.029
HS6ST2	23.96	1.59	0.044
CEBPE	47.63	1.58	< 0.001
LOC786987	820.38	1.54	0.049
GGT1	715.94	1.51	< 0.001
LOC100336635	24.80	1.42	0.005
LOC101903713	40.66	1.42	0.003
LRRN3	45.34	1.38	0.019
C8H9orf43	67.45	1.38	0.013
SLC43A2	1655.59	1.38	< 0.001
CLCN1	18.45	1.36	0.035
FLVCR2	809.98	1.34	< 0.001
RNF183	32.35	1.34	0.008
SPNS2	64.53	1.32	0.006

FLVCR2-2	63.45	1.32	0.001
ATP6AP1L	22.93	1.32	0.033
ZBTB12	142.81	1.30	0.023
LOC101904201	20.78	1.29	0.009
LOC619159	20.26	1.28	< 0.001
CD300LB	29.31	1.28	0.002
KLF11	615.73	1.28	< 0.001
HILPDA	39.25	1.28	< 0.001
SLC31A1	6171.07	1.28	0.002
IZUMO1	34.66	1.26	0.017
SLC6A8	2027.72	1.25	0.008
GGT5	607.21	1.25	< 0.001
PKD1L2	40.31	1.24	0.007
SLC4A4	7328.16	1.24	0.014
DLEC1	81.91	1.24	< 0.001
OMP	110.21	1.23	0.011
SLC7A7	2413.54	1.23	< 0.001
LOC101908214	19.71	1.23	0.002
FFAR2	111.17	1.23	0.003
IYD	153.29	1.23	0.030
NKAIN4	24.88	1.23	0.022
CCR3	106.24	1.20	< 0.001
LOC101905222	105.77	1.20	< 0.001
FBP1	847.18	1.19	< 0.001
RAB17-2	64.37	1.19	0.001
KCNK13	16.56	1.18	0.008
SCNN1A	130.05	1.17	0.029
SEPP1	5431.10	1.15	< 0.001
ALOX5	453.09	1.14	< 0.001
CDIP1	481.10	1.14	0.001
OLFM4	3405.28	1.13	0.018
MAMDC4	72.54	1.13	0.012
ACE2	3758.84	1.12	0.003
LOC100336592	426.24	1.11	0.005
OTOG	241.17	1.10	0.001
MGAT5B	32.04	1.10	0.001
LDHD	204.86	1.10	0.004
MAPK11	95.68	1.10	0.011
LOC532205	43.30	1.09	0.049
LOC101907926	22.02	1.09	0.012
EMR3	556.30	1.09	< 0.001
UCP2	1314.63	1.09	0.001
MAOB	1499.41	1.08	0.021

GPR39	132.35	1.07	0.007
LOC514978	204.33	1.07	0.001
FOXP3	19.79	1.07	0.013
TDRKH	18.53	1.06	0.043
GUCA2A	2686.86	1.06	0.032
LOC781494	256.08	1.06	0.025
MFSD3	75.34	1.06	< 0.001
LOC100335509	47.72	1.05	0.002
DLX4	34.05	1.05	0.025
GPX3	673.72	1.04	< 0.001
GAL3ST2	18.07	1.04	0.013
MMP9	1487.18	1.04	0.001
TGFA	68.03	1.03	< 0.001
LOC101908208	15.48	1.03	0.049
SLC24A1	29.16	1.03	0.035
FOS	651.50	1.03	< 0.001
PEPD	2122.80	1.03	< 0.001
NR4A1	286.28	1.03	0.002
PDZK1	3335.75	1.03	0.008
WNK4	81.47	1.02	0.006
OIT3	35.03	1.02	0.024
LOC618463	116.67	1.02	0.001
CFD	2745.31	1.02	0.001
CA13	1045.92	1.01	0.014
ANO6	3290.68	1.01	0.002
LOC100335263	19.82	1.01	0.016
LNX1	491.91	1.01	0.020
CYP2D14	966.01	1.00	0.008
EDAR	17.98	0.99	0.027
APOH	123.79	0.97	0.045
GPT	777.70	0.97	0.001
LOC101903503	124.14	0.97	0.025
AGT	107.65	0.97	0.024
LOC101903220	18.04	0.97	0.017
SLC3A2	1233.38	0.97	0.004
PDK4	1518.08	0.96	< 0.001
PFKFB4	898.27	0.96	0.006
TARM1	21.97	0.95	0.024
SH3GL2	424.35	0.95	0.003
CPT1A	2292.73	0.95	< 0.001
HEBP2	253.82	0.95	0.041
GPR4	49.37	0.95	0.003
SLC25A34	2016.32	0.94	< 0.001

ANGPTL4	272.99	0.94	0.006
AQP3	160.14	0.94	0.004
ANXA4	4194.83	0.94	0.037
FAM3B	264.78	0.94	0.012
LOC101908145	62.27	0.94	0.020
LOC537895	220.70	0.92	0.031
LOC101904288	54.15	0.92	0.005
SMPD3	560.91	0.91	< 0.001
TICAM1	158.42	0.91	0.023
ZNF385C	16.15	0.91	0.021
RAB17	235.28	0.91	0.005
ISX	354.27	0.88	< 0.001
ALAD	959.67	0.88	0.001
LOC618565	29.55	0.88	0.036
FGF14	15.68	0.88	0.041
MGAM	5125.37	0.88	0.009
LOC101903281	124.47	0.86	< 0.001
ME2	1990.61	0.86	0.048
CYP4F2-2	2406.04	0.86	0.012
SAMD10	44.63	0.86	0.002
PDZD3	836.68	0.85	< 0.001
LOC508441	86.97	0.85	0.003
FAM19A4	22.03	0.85	0.045
KCNK3	21.31	0.85	0.028
GRHL1	92.95	0.85	0.008
LOC101902612	35.33	0.84	0.027
LOC101902803	23.28	0.84	0.013
PRODH	1273.04	0.84	< 0.001
LOC100848006	107.32	0.83	0.003
WFDC2	274.81	0.83	0.004
ARSA	320.13	0.83	0.001
RASL10B	44.11	0.83	0.014
FCGRT	2687.91	0.83	< 0.001
LOC100301363	156.54	0.83	0.001
PKLR	1115.17	0.83	0.001
LOC101906462	34.13	0.83	0.004
LTB4R	45.28	0.82	0.008
LOC100138951	98.45	0.82	0.020
SCUBE1	312.59	0.82	0.004
GSDMB	4728.76	0.82	0.006
EFNA1	422.14	0.82	< 0.001
LOC101905227	28.89	0.81	0.029
PTCH2	231.67	0.81	< 0.001

PIPOX	334.74	0.81	0.023
LOC509703-2	165.28	0.81	0.001
KLK1	35.65	0.80	0.024
LOC505326	28.88	0.80	0.008
ENO4	33.75	0.80	0.013
LOC101901948	38.77	0.80	0.011
LRRC34	22.15	0.80	0.011
LOC100847453	62.10	0.80	0.043
NR1D1	72.47	0.79	< 0.001
PRAM1	125.10	0.79	< 0.001
FGF9	24.57	0.78	0.023
SLC51A	892.10	0.78	0.014
SLC6A7	398.81	0.78	0.040
PROS1	576.18	0.78	< 0.001
SLC25A20	1457.65	0.78	< 0.001
TMEM253	590.17	0.78	0.001
BEST4	341.64	0.78	0.015
LARP6	188.80	0.78	0.025
PLCD1	827.16	0.78	0.001
LOC100196897	50.78	0.77	0.029
KIFC3	330.60	0.77	0.016
FCAR	91.15	0.77	0.003
HSPA1A	7073.65	0.77	0.008
SH2D4A	184.07	0.76	0.020
TRPV4	123.44	0.76	< 0.001
LOC101904278	52.17	0.76	0.014
LOC101902851	20.74	0.76	0.043
LOC509506	1046.41	0.76	0.038
ADRB3	17.69	0.75	0.049
CPAMD8	46.26	0.75	0.038
CD209	137.30	0.75	0.017
CAPN11	25.75	0.75	0.032
CAMKK1	156.97	0.74	< 0.001
EGR1	129.17	0.74	0.019
DHRS13	54.36	0.74	0.007
LOC100847891	132.76	0.74	0.015
CLSTN3	85.90	0.74	0.017
SLCO4A1	183.92	0.73	0.034
LOC511478	27.92	0.72	0.013
RAB15	31.91	0.72	0.014
CDK5R1	20.53	0.72	0.029
LOC100126043	48.80	0.72	0.016
IHH	558.36	0.71	0.001

TMEM140	229.17	0.71	< 0.001
ESPN	213.18	0.71	0.022
ATP13A2	873.50	0.70	0.011
RETN	71.69	0.70	0.012
CHRD	66.20	0.70	0.002
MMP19	293.91	0.69	< 0.001
gzmA	962.22	0.69	0.034
BCL9L	395.31	0.69	0.001
MROH6	217.74	0.69	0.011
SPTBN5	134.76	0.69	0.005
BTN3A3	412.73	0.69	0.006
SNED1	120.23	0.69	0.007
RIN3	308.91	0.69	0.007
ALDOB	7974.56	0.68	< 0.001
ZDHHC14-2	75.73	0.68	0.002
CNDP2	6540.49	0.68	< 0.001
UNC13D	452.03	0.68	< 0.001
CYGB	252.50	0.68	< 0.001
LOC101904129	461.21	0.68	0.031
DEGS2	330.86	0.68	0.036
HS6ST1	195.62	0.68	0.001
HIC1	238.82	0.68	0.015
NMNAT2	114.14	0.67	0.012
ACP5	2248.87	0.67	0.038
C4BPA	2228.83	0.67	0.043
LOC100848231	117.78	0.67	0.005
MX1	1931.75	0.67	0.042
IL28RA	274.64	0.67	0.007
MYRF	171.78	0.67	0.028
SAG	36.31	0.66	0.021
DFNB31	108.08	0.66	0.003
NLRX1	143.69	0.66	< 0.001
CLDND2	27.70	0.66	0.041
WFS1	203.18	0.66	0.006
SAPCD2	322.35	0.66	0.032
MAF	985.67	0.66	0.012
GYLTL1B	226.85	0.66	0.002
DECR2	820.06	0.66	0.023
SLC16A3	130.67	0.65	< 0.001
GPR35	253.41	0.65	0.006
TPRN	280.02	0.65	0.009
NMUR1	32.26	0.65	0.024
SESN2	402.64	0.65	0.049

GUCA2B	661.58	0.65	0.026
LOC101907969	276.81	0.64	0.001
TLE4	1874.66	0.64	0.042
KIF19	60.71	0.64	0.001
PLIN2	918.97	0.64	< 0.001
SLC16A5	357.50	0.64	0.025
CLN8	181.06	0.64	< 0.001
LOC101902385	53.41	0.64	0.007
DHRS7	472.19	0.64	0.017
XKR8	114.45	0.63	0.004
ZNF385A	68.39	0.63	0.040
NR1H3	713.56	0.63	< 0.001
SLC25A22	603.46	0.63	0.028
AMPD3	312.30	0.63	0.002
NT5DC3	301.99	0.63	0.028
TLCD1	36.28	0.63	0.030
PLEKHH3	150.03	0.62	0.004
MICALCL	192.54	0.62	0.002
F2RL1	544.03	0.62	0.001
LOC511919-2	222.50	0.62	0.003
BSPRY	376.65	0.61	< 0.001
TP53I13	126.62	0.61	0.002
SIRPB1	186.13	0.61	0.003
CASS4	345.44	0.61	0.041
PBX4	80.12	0.61	0.043
ISYNA1	63.28	0.61	0.005
FZD3	456.04	0.60	0.027
PLXND1	376.28	0.60	< 0.001
PCSK4	92.65	0.60	0.002
ZNF135	69.58	0.60	0.047
RILP	73.18	0.60	0.006
GLIS3	200.23	0.60	0.005
VWA2	221.36	0.60	0.008
PUSL1	30.12	0.60	0.045
RASL11A	64.74	0.60	0.011
AGRN	977.63	0.59	0.019
LAPTM4B	3535.42	0.59	0.043
MYO7A	231.60	0.59	< 0.001
FREM1	87.98	0.59	0.027
EBI3	116.17	0.59	0.008
KCNJ2	213.20	0.59	0.006
NFAM1	94.33	0.59	0.017
FAM83G	329.74	0.59	0.011

TMEM98	1248.57	0.59	0.034
RARRES2	5000.94	0.59	0.012
NOTCH1	1560.26	0.59	0.011
TMEM8A	860.46	0.59	0.020
ARSG	218.63	0.58	0.004
AGAP3	201.81	0.58	0.014
FGD2	451.67	0.58	< 0.001
WNT2B	303.55	0.58	0.002
KRBA1	139.98	0.57	0.035
EPPK1	2364.73	0.57	0.024
MGAT3	653.74	0.57	0.003
FBXO2	54.64	0.57	0.029
PCGF2	124.26	0.57	0.005
LOC101902444	88.71	0.57	0.015
LOC100336747	319.93	0.57	0.030
SPSB2	155.33	0.57	0.004
MFSD7	248.60	0.57	0.013
RBBP8NL	58.57	0.57	0.019
LOC100336999	3977.59	0.57	0.046
CRIP1	2956.87	0.57	0.047
UAP1L1	306.03	0.57	0.001
ESPNL	133.23	0.57	0.042
JUN	866.24	0.57	< 0.001
CTGF	73.85	0.57	0.031
VSIG4	235.52	0.57	0.022
LOC785168	730.34	0.57	0.026
OSBPL6	896.91	0.57	< 0.001
TMC4	2024.41	0.56	0.003
LOC100337081	337.24	0.56	0.026
ZFP36	449.45	0.56	0.011
LAMA2	365.47	0.56	0.009
MGC127133	1503.04	0.56	0.013
CAMTA2	1233.36	0.56	0.013
COL23A1	174.05	0.56	0.038
KCNT1	210.75	0.56	0.017
TULP2	134.60	0.56	0.033
HTRA3	84.24	0.56	0.021
PILRA	262.13	0.56	0.012
SCAP	750.53	0.55	0.002
ADAMTS13	90.65	0.55	0.005
HSF4	243.11	0.55	< 0.001
BTG2	585.18	0.55	0.001
CITED2	647.44	0.55	0.003

LOC100335281	2268.91	0.55	0.001
PSTPIP1	748.03	0.55	0.001
PLA2G4B	90.96	0.55	0.020
NPR2	192.63	0.55	0.001
SSBP4	213.72	0.55	0.001
RARA	776.08	0.54	0.006
IRF7	769.01	0.54	0.027
PVRL1	190.02	0.54	0.032
GPT2	739.93	0.54	0.020
LOC511919	198.76	0.54	0.033
SGK1	1766.17	0.54	0.028
PEAR1	223.79	0.54	0.004
TMEM204	138.49	0.54	0.001
DOK2	403.74	0.54	0.001
SHANK3	93.06	0.54	0.032
C3H1orf226	722.69	0.53	0.013
ALS2CL	219.65	0.53	0.014
KIAA1147	195.28	0.53	0.019
LPIN3	412.59	0.53	< 0.001
LOC101906354	31.84	0.53	0.048
CLCN2	1128.54	0.53	0.034
ATG2A	427.20	0.53	0.049
PCYOX1L	77.65	0.53	0.007
C7H19orf59	199.46	0.53	0.047
ABTB1	1275.43	0.53	0.049
SARDH	73.58	0.53	0.037
CHST14	102.41	0.53	0.001
PTK7	288.60	0.53	0.007
PM20D1	1183.25	0.53	0.014
TMEM184B	292.24	0.53	0.031
ZNF783	197.81	0.53	0.004
TUBA1C	334.79	0.53	0.021
FBXL15	72.99	0.52	0.048
AXL	1143.42	0.52	0.001
GATA5	272.18	0.52	0.009
NAPRT1	1998.70	0.52	0.013
LOC616337	744.43	0.52	0.007
SCARF1	335.62	0.52	< 0.001
C21H15orf39	253.24	0.52	0.006
RASGRP4	198.06	0.52	0.001
CISH	346.63	0.52	0.008
LOC525938	198.92	0.52	0.012
ELMO3	1026.97	0.52	< 0.001

KCTD1	102.68	0.51	0.005
MLYCD	181.25	0.51	0.010
APLP1	106.82	0.51	0.041
FUOM	684.80	0.51	0.003
KCNAB2	411.94	0.51	< 0.001
NOXA1	331.38	0.51	0.020
SLC22A5	1441.05	0.51	0.005
FGFRL1	41.96	0.51	0.046
LOC511180	376.93	0.51	0.026
SIGLEC1	126.05	0.51	0.020
LOC101903017	44.37	0.51	0.034
C2CD2L	580.17	0.51	0.002
HNF1A	466.99	0.50	0.004
LRP1	9599.10	0.50	0.028
LOC101904478	85.38	0.50	0.036
METRNL	482.16	0.50	0.039
KIFC2	113.31	0.50	0.021
CKS2	291.03	-0.50	0.003
APEX1	772.79	-0.50	< 0.001
LSM3	302.86	-0.50	< 0.001
FKBP5	1782.64	-0.50	< 0.001
RAI14	701.84	-0.50	0.001
MCCC1	523.55	-0.50	< 0.001
DBF4	252.72	-0.51	0.001
NASP	1414.80	-0.51	< 0.001
KBTBD8	197.14	-0.51	0.003
MCM5	652.32	-0.51	0.023
FAM83D	151.48	-0.51	0.005
KPNA2	1202.61	-0.52	< 0.001
C10H14orf1	563.30	-0.52	0.033
FLRT2	457.96	-0.52	0.030
AURKA	352.06	-0.52	< 0.001
CDCA7L	281.21	-0.52	0.015
TEX30	141.23	-0.52	0.003
HMGB1	3614.46	-0.52	0.004
MLF1IP	126.21	-0.52	0.016
PDE5A-2	401.86	-0.52	0.026
ZC3H12D	241.51	-0.52	0.027
PLOD2	272.16	-0.52	0.039
CCDC58	169.30	-0.53	0.011
KCNQ5	96.54	-0.53	0.001
HAT1	512.84	-0.53	0.002
ECT2	692.75	-0.53	0.002

SGOL1	197.38	-0.53	0.040
NME2	697.70	-0.53	0.023
DTL	267.95	-0.53	0.011
LOC509946	78.37	-0.53	0.045
HIST1H2BM	771.74	-0.53	0.042
BASP1	503.32	-0.53	0.044
ANLN	643.77	-0.53	0.004
TNC	1660.13	-0.53	0.006
THOC7	210.59	-0.54	0.012
CDK1	992.44	-0.54	0.001
PRC1	640.76	-0.54	0.006
TICRR	362.26	-0.54	0.028
HIST1H1E	332.16	-0.54	0.013
ROBO1	359.73	-0.54	0.031
MMS22L	313.16	-0.54	0.030
LOC786372	1296.96	-0.54	0.031
RAD54L	309.87	-0.54	0.029
TOPBP1	2017.98	-0.54	< 0.001
FEN1	293.04	-0.55	0.012
RPL22L1	421.00	-0.55	0.012
CETN4	40.72	-0.55	0.043
SGOL2	1288.33	-0.55	0.010
VKORC1L1	660.26	-0.55	0.002
KIF24	244.78	-0.55	< 0.001
RCAN1	218.56	-0.55	0.040
PLEKHG1-2	711.64	-0.55	0.006
HIST1H1D	3551.82	-0.55	0.018
NAP1L1	2647.75	-0.55	0.036
DEPDC1	469.79	-0.55	0.015
KIF22	645.38	-0.55	0.021
HIST1H1E-2	3864.55	-0.55	0.015
MGME1	189.88	-0.55	0.031
CKAP2L	428.29	-0.55	0.012
PLK4	440.83	-0.55	0.002
PLEKHG1	95.71	-0.56	0.049
RFC4	186.88	-0.56	0.010
SPC24	193.90	-0.56	< 0.001
SLC16A7	107.05	-0.56	0.001
BRCA1	903.48	-0.56	0.013
LOC614159	315.31	-0.56	0.029
EID1	307.27	-0.56	0.021
SNRNP25	163.58	-0.56	0.013
ZNF367	215.23	-0.56	0.010

TXN	1234.75	-0.56	0.036
DTYMK	123.41	-0.57	0.017
CCNE1	83.91	-0.57	0.036
SUMO2	881.54	-0.57	0.006
UNG	352.54	-0.57	0.024
NFE2L3	285.20	-0.57	0.002
HMGN5	79.16	-0.57	0.007
RAD18	222.13	-0.58	< 0.001
CCDC34	117.01	-0.58	0.007
LRRK2	2070.84	-0.58	0.042
SMC4	2324.23	-0.58	< 0.001
CDON	556.70	-0.58	0.025
VRK1	365.26	-0.58	< 0.001
LOC512869	4154.16	-0.59	0.015
RFC2	381.89	-0.59	0.003
HIST1H2AC	1425.61	-0.59	0.006
LOC101905203	57.98	-0.59	0.033
HMGB2	1557.56	-0.59	0.032
CDCA7	1339.79	-0.59	0.007
C29H11orf54	565.30	-0.59	0.045
HIST1H2BD	1639.36	-0.59	0.014
CBX3	545.31	-0.59	< 0.001
KIF4A	643.43	-0.59	0.011
CDCA2	337.57	-0.59	0.020
PARPBP	219.14	-0.59	0.003
PGAP1	122.30	-0.59	0.014
PHGDH	554.09	-0.59	0.013
FAM76B	553.49	-0.60	0.037
H2AFZ	934.45	-0.60	< 0.001
PPP1R14A	107.73	-0.60	0.025
DPCD	209.42	-0.60	0.005
MASTL	203.20	-0.60	0.005
PCP4L1	104.08	-0.60	0.028
MPP6	162.81	-0.60	0.033
CENPQ	99.49	-0.60	0.007
GIN1	117.02	-0.60	< 0.001
FAM69A	183.07	-0.60	0.001
CDKN3	96.32	-0.60	0.020
GINS2	92.17	-0.60	0.022
SLAIN1	146.69	-0.61	0.007
PRKD3	1593.18	-0.61	0.032
KIF2C	298.55	-0.61	0.010
MCM8	121.75	-0.61	0.009

SLC16A1	940.98	-0.61	0.010
LOC101902723	105.42	-0.61	0.005
SERPINI1	55.22	-0.61	0.018
LOC100847721	336.07	-0.62	0.019
LOC527388	1751.66	-0.62	0.013
LOC539572	596.14	-0.62	< 0.001
TNFRSF10B	285.04	-0.62	0.015
IKZF2	995.40	-0.62	0.028
SPC25	426.03	-0.62	0.001
MND1	104.58	-0.62	0.016
CENPN	103.68	-0.62	0.002
ADK	679.08	-0.63	0.041
LOC523214	659.70	-0.63	0.012
POSTN	4416.33	-0.63	0.008
TCEAL8	522.50	-0.63	0.048
TNFRSF18	42.87	-0.63	0.045
PAICS	1149.30	-0.63	0.006
LOC100848433	132.51	-0.64	0.003
RRM2	918.16	-0.64	< 0.001
MTBP	246.18	-0.64	0.003
ANP32E	541.23	-0.64	0.002
ACAT1	1073.08	-0.64	0.033
SHCBP1	275.77	-0.64	< 0.001
ATAD5	457.44	-0.64	0.002
CDCA3	212.32	-0.64	0.008
HIST1H2BB	1196.34	-0.64	0.014
RRM2B	331.72	-0.64	0.022
TRIM34	399.63	-0.64	0.015
ARL11	47.79	-0.65	0.026
WDHD1	363.80	-0.65	< 0.001
ZWILCH	573.41	-0.65	< 0.001
CCDC109B	202.75	-0.65	< 0.001
CDKN2C	164.89	-0.65	0.007
KIF20B	833.79	-0.65	< 0.001
CYP2R1	170.20	-0.65	0.019
TLR2	105.27	-0.65	0.002
CEP55	237.07	-0.65	0.013
DUT	472.18	-0.65	0.001
GAS2	107.81	-0.65	0.025
CFL2	475.37	-0.65	0.031
DHFR	586.54	-0.66	< 0.001
KLHL23	189.45	-0.66	0.020
POLE	561.74	-0.66	0.011

HIST1H2AH	1068.99	-0.66	0.024
CASC5	1495.02	-0.66	0.004
CCT6B	49.53	-0.66	0.015
MAD2L1	193.45	-0.66	< 0.001
S1PR3	118.80	-0.66	0.004
IL18RAP	32.17	-0.66	0.046
SCRN3	172.03	-0.66	0.008
FANCB	61.68	-0.66	0.021
PACRGL	62.82	-0.67	0.017
SKA2	280.65	-0.67	< 0.001
ESCO2	243.71	-0.67	< 0.001
CDCA8	300.61	-0.67	0.013
PBK	276.30	-0.67	0.003
LMNB1	978.38	-0.67	0.001
CCNA2	360.27	-0.67	0.001
BRCA2	1071.95	-0.67	0.001
MIS18BP1	626.53	-0.67	0.002
MCM3	997.49	-0.67	0.011
SGCD	162.69	-0.68	0.020
BARD1	194.30	-0.68	0.022
CD72	96.82	-0.68	0.012
LOC517139	404.36	-0.68	< 0.001
FAM175A	189.79	-0.68	0.031
ASPM	1563.08	-0.68	0.009
DACT3	77.90	-0.69	0.044
OIP5	132.87	-0.69	0.001
HMMR	460.20	-0.69	< 0.001
IL20RB	24.85	-0.69	0.043
KIF18A	262.50	-0.69	0.004
BCL2A1	229.35	-0.69	< 0.001
BUB1B	933.74	-0.69	0.009
CFTR	1374.31	-0.69	0.032
MELK	273.26	-0.69	0.001
FGF12	33.36	-0.69	0.035
LOC516742	859.23	-0.69	0.024
UBE2C	481.35	-0.69	0.003
AURKB	394.24	-0.69	0.021
EXO1	178.82	-0.70	0.005
LOC101906009	46.44	-0.70	0.016
MCM6	1294.20	-0.70	0.006
LOC100848761	31.39	-0.70	0.023
TMEM123	1468.14	-0.70	0.020
TEX12	63.23	-0.70	0.021

GBP5	585.00	-0.70	0.008
LOC521580	1495.47	-0.70	0.008
E2F8	200.85	-0.71	0.005
LOC787465	883.86	-0.71	0.036
BUB1	824.79	-0.71	0.007
SMC2	1719.78	-0.71	< 0.001
CNTNAP2	87.03	-0.71	0.015
TPX2	1344.76	-0.71	0.005
LOC618824	1746.49	-0.71	0.022
CD40	267.49	-0.71	0.005
RASGRP3	1066.60	-0.71	0.045
CENPK	76.79	-0.72	0.004
HIST1H2BN	3009.24	-0.72	0.013
CLSPN	603.18	-0.73	0.026
TCEA1	1076.06	-0.73	0.033
BRIP1	265.78	-0.73	0.001
SPAG5	608.56	-0.73	0.006
CCNB1	403.17	-0.73	0.005
CCNB2	368.92	-0.73	0.003
MORN4	46.09	-0.73	0.003
RAB27A	606.00	-0.74	0.003
NCAPG	970.80	-0.74	0.001
HIST1H1B	4599.44	-0.74	0.004
DCK	556.27	-0.74	< 0.001
ARHGAP11A	887.33	-0.74	< 0.001
DEPDC1B	122.30	-0.74	0.002
KNTC1	630.71	-0.74	0.001
CCNE2	124.54	-0.74	0.038
CENPE	1565.29	-0.75	< 0.001
NEIL3	88.67	-0.75	0.011
GABRB2	39.11	-0.75	0.009
FAM64A	63.27	-0.75	0.029
LOC616819	1370.08	-0.75	0.035
HIST1H2AD	1475.39	-0.76	0.009
APITD1	204.10	-0.76	< 0.001
NUF2	288.55	-0.76	< 0.001
TOP2A	4395.56	-0.76	0.001
PNLDC1	54.22	-0.76	0.004
MCM10	479.38	-0.76	0.001
CRCP	326.32	-0.76	0.035
PKMYT1	66.96	-0.76	0.020
CDC6	190.93	-0.76	0.026
LOC101905266	260.61	-0.76	0.043

LOC100140850	231.71	-0.77	0.049
LOC616611	337.40	-0.77	0.046
C1H3orf70	374.12	-0.77	0.031
SLC22A15	86.96	-0.77	0.004
DIAPH3	259.78	-0.77	0.002
NQO1	204.36	-0.78	0.025
KIAA0101	554.49	-0.78	< 0.001
LOC540148	1059.63	-0.78	0.016
HIST2H2BF	801.06	-0.78	0.005
GEN1	301.72	-0.78	< 0.001
ERCC6L	218.71	-0.78	0.002
PCNA	1104.48	-0.78	0.001
SPDL1	157.83	-0.78	< 0.001
SMTN	1119.98	-0.79	0.034
NDC80	334.85	-0.79	0.001
NDC1	617.04	-0.79	0.011
LOC504599	232.18	-0.79	0.031
LOC537017	1919.18	-0.79	0.025
HELLS	446.77	-0.80	0.001
LOC616800	562.97	-0.80	0.010
CNTNAP4	53.69	-0.81	0.003
MSH2	619.67	-0.81	0.001
SKA1	87.83	-0.81	0.001
CENPF	3831.27	-0.82	0.007
RPA3	172.22	-0.82	< 0.001
LOC100847609	42.53	-0.82	0.007
ERO1LB	645.76	-0.82	0.006
TAGLN	4214.01	-0.82	0.033
NEXN	239.42	-0.82	0.044
FADS1	212.95	-0.82	0.003
CENPP	60.83	-0.82	< 0.001
DNAH5	19.82	-0.83	0.046
LOC101902226	84.09	-0.83	0.008
LTB	219.00	-0.83	0.032
HIST1H2AG	1458.14	-0.84	0.033
KIRREL2	83.56	-0.85	0.026
GRAMD2	81.48	-0.85	0.011
SBSPON	15.13	-0.86	0.048
MYLK	5198.98	-0.86	0.015
MYL9	1795.39	-0.86	0.017
TPM1	4449.93	-0.86	0.017
TMEM158	27.36	-0.86	0.009
FGF7	53.21	-0.87	0.004

DMPK	566.61	-0.87	0.033
MYH11	30785.83	-0.87	0.038
LOC505183	1277.55	-0.88	0.008
NUSAP1	438.94	-0.88	0.007
LOC101905399	41.16	-0.88	0.012
PLN	398.36	-0.89	0.022
LOC534578	912.13	-0.89	< 0.001
DYX1C1	27.58	-0.89	0.048
ORC1	239.63	-0.89	0.005
LOC101907303	94.36	-0.90	0.033
DLGAP5	602.86	-0.90	< 0.001
KCNN3	540.13	-0.90	0.044
KCNMB3	50.12	-0.91	0.033
SWAP70	945.45	-0.91	0.021
FANCD2	280.14	-0.91	0.011
SH3BGR	295.11	-0.92	0.017
SFRP5	46.16	-0.92	0.023
ACSL3	669.10	-0.93	< 0.001
SLC6A12	43.87	-0.93	0.046
LOC513486	67.03	-0.93	0.001
KIF11	1024.47	-0.94	< 0.001
DCLK1	104.79	-0.94	0.004
SORBS2	502.38	-0.94	0.034
KLHL6	454.34	-0.94	0.034
LOC101906058	220.67	-0.94	0.021
LOC522960	769.46	-0.95	0.009
RAPGEF5	1919.31	-0.95	0.037
PIF1	40.49	-0.95	0.022
KIF15	892.26	-0.95	0.003
LOC786060	42.45	-0.95	0.016
CENPM	134.18	-0.95	< 0.001
LOC528329	148.44	-0.96	0.045
HCAR3	17.44	-0.97	0.037
XYLT1	186.39	-0.97	0.044
CHN1	55.13	-0.97	0.008
LOC100848985	32.83	-0.98	0.009
DUSP27	32.66	-0.98	0.026
LOC100848911	176.60	-0.99	< 0.001
CYP51A1	2038.28	-1.00	0.026
ADA	403.33	-1.00	0.006
EAF2	299.29	-1.00	0.036
ANO4	26.89	-1.01	0.029
SYNM	7237.96	-1.02	0.034

MYBL2	426.46	-1.02	0.016
LOC100848734	883.30	-1.02	0.007
FGL1	53.10	-1.02	0.032
LRP8	91.79	-1.02	0.009
EPHA7	77.37	-1.02	0.036
FCER1A	146.44	-1.03	0.032
HOMER2	89.47	-1.03	0.041
CTH	43.68	-1.03	0.004
LOC100336949	902.07	-1.03	0.017
BLK	169.78	-1.03	< 0.001
TPM2	4812.27	-1.04	0.023
HIST1H2BB-2	131.36	-1.05	0.002
EPN3	52.98	-1.05	0.005
HIST1H2AJ	884.01	-1.05	< 0.001
WDR87	43.43	-1.05	0.028
SFRP2	35.66	-1.05	0.037
PDGFRL	36.03	-1.05	0.045
ACTN2	34.35	-1.05	0.021
CD19	196.52	-1.06	0.029
METTL7A	383.26	-1.07	0.006
SLC7A10	35.18	-1.07	0.048
DNAJB5	72.59	-1.07	0.004
HSPB6	43.24	-1.08	0.028
CXCL6	325.78	-1.09	0.008
CCL19	50.12	-1.10	0.043
SORCS1	25.56	-1.10	0.029
FAM46B	54.30	-1.11	0.005
PYGM	84.79	-1.11	0.020
SMPX	61.89	-1.12	0.012
GSTO1	118.04	-1.12	0.039
SCD	2419.44	-1.14	0.006
LOC504806-2	188.07	-1.14	0.017
ATP6V0D2	67.80	-1.14	0.013
KIAA1210	23.07	-1.15	0.012
PTPRZ1	29.67	-1.16	0.028
AKR1E2	18.08	-1.17	0.004
FNDC1	47.02	-1.18	0.034
S1PR2	215.71	-1.18	0.030
FCAMR	295.62	-1.18	0.001
CAPSL	20.12	-1.18	0.011
GLDC	21.80	-1.18	0.017
POPDC2	54.78	-1.19	0.019
TARS	6212.45	-1.19	0.023

IL10	25.36	-1.20	0.004
PNCK	29.12	-1.20	0.019
WASF1	22.73	-1.20	0.041
WDR76	538.36	-1.21	0.012
LRRN1	82.09	-1.21	0.042
CA14	33.45	-1.21	0.037
LPL-2	253.57	-1.21	0.002
COL11A1	26.24	-1.22	0.036
PSAT1	214.66	-1.22	0.002
AMACR	132.96	-1.22	0.035
FCRL5	146.11	-1.23	0.001
FMOD	282.50	-1.24	0.005
ARG2	49.49	-1.26	0.023
CENPW	17.11	-1.27	0.013
CNKSR2	26.30	-1.27	0.040
VIP	289.57	-1.34	0.005
LOC781749	18.28	-1.34	0.006
PI15	317.21	-1.34	0.001
ADM2	58.38	-1.36	0.043
LPL	31.19	-1.37	0.003
LPAR3	62.18	-1.39	0.007
ACTG2	6038.11	-1.39	0.006
STMN1	1580.15	-1.40	0.004
FAM184A	54.23	-1.41	0.015
F11	31.19	-1.41	0.030
C5H12orf75	108.31	-1.43	0.013
TKTL2	22.23	-1.43	0.032
RMI2	134.18	-1.46	0.001
HIST1H1A	267.84	-1.47	< 0.001
CA10	20.96	-1.47	0.002
LOC786126	51.49	-1.47	0.011
FADS3	92.45	-1.47	0.047
NTRK2	49.80	-1.48	0.040
LOC613595	35.22	-1.49	0.003
CERS4	24.01	-1.50	0.043
PCP4	194.45	-1.53	0.006
PKIA	312.55	-1.54	0.006
CLCN4	65.15	-1.55	0.017
B3GALT5	27.54	-1.58	0.037
DUOX2	35.84	-1.59	0.037
S100A2	47.66	-1.60	0.023
LOC100337413	36.35	-1.64	0.004
MCOLN3	70.94	-1.66	0.027

NEBL	101.59	-1.69	0.010
CDH26	45.49	-1.69	0.017
GRB14	35.62	-1.69	0.010
MOXD1	48.17	-1.70	0.008
POU2AF1	485.30	-1.71	0.004
CD22	427.03	-1.72	0.027
BCAT1	193.60	-1.74	0.001
PLA2G3	38.75	-1.76	0.027
LOC100847119	1377.90	-1.77	0.001
SLC9B2-2	47.04	-1.77	0.019
AFF2	271.13	-1.86	0.027
LOC787803	251.72	-1.92	0.002
COL8A1	44.79	-1.92	0.007
STRA6	24.65	-1.92	0.014
CLGN	68.11	-2.03	0.006
NAPSA	99.74	-2.03	0.045
BACH2	236.11	-2.06	0.013
PLA2G5	47.86	-2.10	< 0.001
FCRL1	75.03	-2.15	0.007
SLC9B2	32.44	-2.17	0.012
AP3B2	46.04	-2.28	0.009
TSPAN1	36.00	-2.31	0.004
LOC616323	243.57	-2.36	0.004
EHD3	297.08	-2.45	0.017
VPREB3	168.31	-2.88	0.010
LOC100847618	66.79	-3.10	< 0.001
CD79B	203.42	-3.11	0.006
CXCL13	117.43	-3.12	0.006
CD180	121.41	-3.33	0.003
CYP2B6	233.94	-3.43	< 0.001
RGS13	133.95	-3.51	0.001
CR2	714.24	-3.53	0.002
FCRL4	160.26	-3.64	0.009
MS4A1	432.25	-3.70	0.001
ACOT2-2	171.45	-3.86	< 0.001
ELL3	134.81	-3.87	0.003
PAX5	341.44	-4.82	< 0.001
FCRLA	274.86	-5.11	< 0.001
