# Supplementary material for: Analysis of Host Jejunum Transcriptome and Associated Microbial Community Structure Variation in Young Calves with Feed-Induced Acidosis
Source: Metabolites. 2021 Jun 23;11(7):414. doi: 10.3390/metabo11070414 (PMC8303401; doi:10.3390/metabo11070414)
Supplement: Supplementary file 1 [file metabolites-11-00414-s001.zip › Supplemental Table S2_Functional annotation with z-score.pdf]

Supplemental Table S2 Functional annotation of DEGs with z-score

Category	ID	Term	Count	Genes	Log_2FC	p-value	FDR	z-score
GOTERM_CC	GO:0005887	integral component of plasma membrane	53	*TSPAN1*	-2.31	0.001	1.69E+00	2.34
GOTERM_CC	GO:0005887	integral component of plasma membrane	53	*GABRB2*	-0.75	0.001	1.69E+00	2.34
GOTERM_CC	GO:0005887	integral component of plasma membrane	53	*F2RL1*	0.62	0.001	1.69E+00	2.34
GOTERM_CC	GO:0005887	integral component of plasma membrane	53	*SLC7A8*	2.26	0.001	1.69E+00	2.34
GOTERM_CC	GO:0005887	integral component of plasma membrane	53	*AQP7*	2.24	0.001	1.69E+00	2.34
GOTERM_CC	GO:0005887	integral component of plasma membrane	53	*KCNK13*	1.18	0.001	1.69E+00	2.34
GOTERM_CC	GO:0005887	integral component of plasma membrane	53	*AQP3*	0.94	0.001	1.69E+00	2.34
GOTERM_CC	GO:0005887	integral component of plasma membrane	53	*SLC7A7*	1.23	0.001	1.69E+00	2.34
GOTERM_CC	GO:0005887	integral component of plasma membrane	53	*SLC16A1*	-0.61	0.001	1.69E+00	2.34
GOTERM_CC	GO:0005887	integral component of plasma membrane	53	*LTB4R*	0.82	0.001	1.69E+00	2.34
GOTERM_CC	GO:0005887	integral component of plasma membrane	53	*SLC24A1*	1.03	0.001	1.69E+00	2.34
GOTERM_CC	GO:0005887	integral component of plasma membrane	53	*MS4A1*	-3.70	0.001	1.69E+00	2.34
GOTERM_CC	GO:0005887	integral component of plasma membrane	53	*TGFA*	1.03	0.001	1.69E+00	2.34
GOTERM_CC	GO:0005887	integral component of plasma membrane	53	*TRPV4*	0.76	0.001	1.69E+00	2.34
GOTERM_CC	GO:0005887	integral component of plasma membrane	53	*SLC22A5*	0.51	0.001	1.69E+00	2.34
GOTERM_CC	GO:0005887	integral component of plasma membrane	53	*SLC4A4*	1.24	0.001	1.69E+00	2.34
GOTERM_CC	GO:0005887	integral component of plasma membrane	53	*SLC43A2*	1.38	0.001	1.69E+00	2.34
GOTERM_CC	GO:0005887	integral component of plasma membrane	53	*PILRA*	0.56	0.001	1.69E+00	2.34
GOTERM_CC	GO:0005887	integral component of plasma membrane	53	*SLCO4A1*	0.73	0.001	1.69E+00	2.34
GOTERM_CC	GO:0005887	integral component of plasma membrane	53	*SLC7A10*	-1.07	0.001	1.69E+00	2.34
GOTERM_CC	GO:0005887	integral component of plasma membrane	53	*CD40*	-0.71	0.001	1.69E+00	2.34
GOTERM_CC	GO:0005887	integral component of plasma membrane	53	*SLC26A6*	1.78	0.001	1.69E+00	2.34
GOTERM_CC	GO:0005887	integral component of plasma membrane	53	*CLCN1*	1.36	0.001	1.69E+00	2.34

GOTERM_CC	GO:0005887	integral component of plasma membrane	53	*CLCN2*	0.53	0.001	1.69E+00	2.34
GOTERM_CC	GO:0005887	integral component of plasma membrane	53	*MFSD3*	1.06	0.001	1.69E+00	2.34
GOTERM_CC	GO:0005887	integral component of plasma membrane	53	*PTK7*	0.53	0.001	1.69E+00	2.34
GOTERM_CC	GO:0005887	integral component of plasma membrane	53	*KCNJ2*	0.59	0.001	1.69E+00	2.34
GOTERM_CC	GO:0005887	integral component of plasma membrane	53	*CD72*	-0.68	0.001	1.69E+00	2.34
GOTERM_CC	GO:0005887	integral component of plasma membrane	53	*GPR4*	0.95	0.001	1.69E+00	2.34
GOTERM_CC	GO:0005887	integral component of plasma membrane	53	*BEST4*	0.78	0.001	1.69E+00	2.34
GOTERM_CC	GO:0005887	integral component of plasma membrane	53	*ADRB3*	0.75	0.001	1.69E+00	2.34
GOTERM_CC	GO:0005887	integral component of plasma membrane	53	*PVRL1*	0.54	0.001	1.69E+00	2.34
GOTERM_CC	GO:0005887	integral component of plasma membrane	53	*TNFRSF18*	-0.63	0.001	1.69E+00	2.34
GOTERM_CC	GO:0005887	integral component of plasma membrane	53	*PLXND1*	0.60	0.001	1.69E+00	2.34
GOTERM_CC	GO:0005887	integral component of plasma membrane	53	*SCNN1A*	1.17	0.001	1.69E+00	2.34
GOTERM_CC	GO:0005887	integral component of plasma membrane	53	*FCER1A*	-1.03	0.001	1.69E+00	2.34
GOTERM_CC	GO:0005887	integral component of plasma membrane	53	*FLRT2*	-0.52	0.001	1.69E+00	2.34
GOTERM_CC	GO:0005887	integral component of plasma membrane	53	*SLC6A12*	-0.93	0.001	1.69E+00	2.34
GOTERM_CC	GO:0005887	integral component of plasma membrane	53	*NPR2*	0.55	0.001	1.69E+00	2.34
GOTERM_CC	GO:0005887	integral component of plasma membrane	53	*ATP13A2*	0.70	0.001	1.69E+00	2.34
GOTERM_CC	GO:0005887	integral component of plasma membrane	53	*CD180*	-3.33	0.001	1.69E+00	2.34
GOTERM_CC	GO:0005887	integral component of plasma membrane	53	*KCNK3*	0.85	0.001	1.69E+00	2.34
GOTERM_CC	GO:0005887	integral component of plasma membrane	53	*SLC16A3*	0.65	0.001	1.69E+00	2.34
GOTERM_CC	GO:0005887	integral component of plasma membrane	53	*GPR35*	0.65	0.001	1.69E+00	2.34
GOTERM_CC	GO:0005887	integral component of plasma membrane	53	*SLC16A5*	0.64	0.001	1.69E+00	2.34
GOTERM_CC	GO:0005887	integral component of plasma membrane	53	*EPHA7*	-1.02	0.001	1.69E+00	2.34
GOTERM_CC	GO:0005887	integral component of plasma membrane	53	*SLC6A7*	0.78	0.001	1.69E+00	2.34
GOTERM_CC	GO:0005887	integral component of plasma membrane	53	*SLC16A7*	-0.56	0.001	1.69E+00	2.34

GOTERM_CC	GO:0005887	integral component of plasma membrane	53	*SLC6A8*	1.25	0.001	1.69E+00	2.34
GOTERM_CC	GO:0005887	integral component of plasma membrane	53	*CDON*	-0.58	0.001	1.69E+00	2.34
GOTERM_CC	GO:0005887	integral component of plasma membrane	53	*NTRK2*	-1.48	0.001	1.69E+00	2.34
GOTERM_CC	GO:0005887	integral component of plasma membrane	53	*CD79B*	-3.11	0.001	1.69E+00	2.34
GOTERM_CC	GO:0005887	integral component of plasma membrane	53	*CLCN4*	-1.55	0.001	1.69E+00	2.34
GOTERM_BP	GO:0035725	sodium ion transmembrane transport	5	*SLC6A8*	1.25	0.011	1.73E+01	2.24
GOTERM_BP	GO:0035725	sodium ion transmembrane transport	5	*SLC24A1*	1.03	0.011	1.73E+01	2.24
GOTERM_BP	GO:0035725	sodium ion transmembrane transport	5	*SLC4A4*	1.24	0.011	1.73E+01	2.24
GOTERM_BP	GO:0035725	sodium ion transmembrane transport	5	*SCNN1A*	1.17	0.011	1.73E+01	2.24
GOTERM_BP	GO:0035725	sodium ion transmembrane transport	5	*ANO6*	1.01	0.011	1.73E+01	2.24
GOTERM_BP	GO:0032868	response to insulin	5	*EGR1*	0.74	0.028	3.93E+01	2.24
GOTERM_BP	GO:0032868	response to insulin	5	*TRPV4*	0.76	0.028	3.93E+01	2.24
GOTERM_BP	GO:0032868	response to insulin	5	*SESN2*	0.65	0.028	3.93E+01	2.24
GOTERM_BP	GO:0032868	response to insulin	5	*SCAP*	0.55	0.028	3.93E+01	2.24
GOTERM_BP	GO:0032868	response to insulin	5	*PCK1*	3.19	0.028	3.93E+01	2.24
GOTERM_BP	GO:0035914	skeletal muscle cell differentiation	8	*EGR1*	0.74	0.002	3.52E+00	2.12
GOTERM_BP	GO:0035914	skeletal muscle cell differentiation	8	*FOS*	1.03	0.002	3.52E+00	2.12
GOTERM_BP	GO:0035914	skeletal muscle cell differentiation	8	*NOTCH1*	0.59	0.002	3.52E+00	2.12
GOTERM_BP	GO:0035914	skeletal muscle cell differentiation	8	*BTG2*	0.55	0.002	3.52E+00	2.12
GOTERM_BP	GO:0035914	skeletal muscle cell differentiation	8	*NR4A1*	1.03	0.002	3.52E+00	2.12
GOTERM_BP	GO:0035914	skeletal muscle cell differentiation	8	*PAX5*	-4.82	0.002	3.52E+00	2.12
GOTERM_BP	GO:0035914	skeletal muscle cell differentiation	8	*BCL9L*	0.69	0.002	3.52E+00	2.12
GOTERM_BP	GO:0035914	skeletal muscle cell differentiation	8	*CITED2*	0.55	0.002	3.52E+00	2.12
KEGG_PATHWAY	bta04974	Protein digestion and absorption	8	*SLC15A1*	1.82	0.071	6.17E+01	2.12
KEGG_PATHWAY	bta04974	Protein digestion and absorption	8	*SLC3A2*	0.97	0.071	6.17E+01	2.12

KEGG_PATHWAY	bta04974	Protein digestion and absorption	8	*SLC7A8*	2.26	0.071	6.17E+01	2.12
KEGG_PATHWAY	bta04974	Protein digestion and absorption	8	*ACE2*	1.12	0.071	6.17E+01	2.12
KEGG_PATHWAY	bta04974	Protein digestion and absorption	8	*COL11A1*	-1.22	0.071	6.17E+01	2.12
KEGG_PATHWAY	bta04974	Protein digestion and absorption	8	*SLC1A1*	2.91	0.071	6.17E+01	2.12
KEGG_PATHWAY	bta04974	Protein digestion and absorption	8	*SLC7A7*	1.23	0.071	6.17E+01	2.12
KEGG_PATHWAY	bta04974	Protein digestion and absorption	8	*XPNPEP2*	1.91	0.071	6.17E+01	2.12
GOTERM_BP	GO:0051260	protein homooligomerization	11	*OLFM4*	1.13	0.039	4.95E+01	2.11
GOTERM_BP	GO:0051260	protein homooligomerization	11	*ALAD*	0.88	0.039	4.95E+01	2.11
GOTERM_BP	GO:0051260	protein homooligomerization	11	*SPTBN5*	0.69	0.039	4.95E+01	2.11
GOTERM_BP	GO:0051260	protein homooligomerization	11	*SCUBE1*	0.82	0.039	4.95E+01	2.11
GOTERM_BP	GO:0051260	protein homooligomerization	11	*FGFRL1*	0.51	0.039	4.95E+01	2.11
GOTERM_BP	GO:0051260	protein homooligomerization	11	*KCTD1*	0.51	0.039	4.95E+01	2.11
GOTERM_BP	GO:0051260	protein homooligomerization	11	*ECT2*	-0.53	0.039	4.95E+01	2.11
GOTERM_BP	GO:0051260	protein homooligomerization	11	*SLC1A1*	2.91	0.039	4.95E+01	2.11
GOTERM_BP	GO:0051260	protein homooligomerization	11	*EHD3*	-2.45	0.039	4.95E+01	2.11
GOTERM_BP	GO:0051260	protein homooligomerization	11	*LNX1*	1.01	0.039	4.95E+01	2.11
GOTERM_BP	GO:0051260	protein homooligomerization	11	*VWA2*	0.60	0.039	4.95E+01	2.11
GOTERM_BP	GO:0006000	fructose metabolic process	3	*PFKFB4*	0.96	0.039	4.98E+01	1.73
GOTERM_BP	GO:0006000	fructose metabolic process	3	*ALDOB*	0.68	0.039	4.98E+01	1.73
GOTERM_BP	GO:0006000	fructose metabolic process	3	*FBP1*	1.19	0.039	4.98E+01	1.73
GOTERM_MF	GO:0015179	L-amino acid transmembrane transporter activity	3	*SLC7A8*	2.26	0.058	5.87E+01	1.73
GOTERM_MF	GO:0015179	L-amino acid transmembrane transporter activity	3	*SLC43A2*	1.38	0.058	5.87E+01	1.73
GOTERM_MF	GO:0015179	L-amino acid transmembrane transporter	3	*SLC7A7*	1.23	0.058	5.87E+01	1.73

		activity						
GOTERM_BP	GO:0046330	positive regulation of JNK cascade	6	*FGF19*	2.56	0.044	5.41E+01	1.63
GOTERM_BP	GO:0046330	positive regulation of JNK cascade	6	*CTGF*	0.57	0.044	5.41E+01	1.63
GOTERM_BP	GO:0046330	positive regulation of JNK cascade	6	*F2RL1*	0.62	0.044	5.41E+01	1.63
GOTERM_BP	GO:0046330	positive regulation of JNK cascade	6	*TRPV4*	0.76	0.044	5.41E+01	1.63
GOTERM_BP	GO:0046330	positive regulation of JNK cascade	6	*CCL19*	-1.10	0.044	5.41E+01	1.63
GOTERM_BP	GO:0046330	positive regulation of JNK cascade	6	*EDAR*	0.99	0.044	5.41E+01	1.63
GOTERM_BP	GO:0050728	negative regulation of inflammatory response	6	*ZFP36*	0.56	0.072	7.22E+01	1.63
GOTERM_BP	GO:0050728	negative regulation of inflammatory response	6	*ACP5*	0.67	0.072	7.22E+01	1.63
GOTERM_BP	GO:0050728	negative regulation of inflammatory response	6	*NLRX1*	0.66	0.072	7.22E+01	1.63
GOTERM_BP	GO:0050728	negative regulation of inflammatory response	6	*METRNL*	0.50	0.072	7.22E+01	1.63
GOTERM_BP	GO:0050728	negative regulation of inflammatory response	6	*ADA*	-1.00	0.072	7.22E+01	1.63
GOTERM_BP	GO:0050728	negative regulation of inflammatory response	6	*NR1H3*	0.63	0.072	7.22E+01	1.63
GOTERM_BP	GO:1901201	regulation of extracellular matrix assembly	2	*NOTCH1*	0.59		7.61E+01	1.41
GOTERM_BP	GO:1901201	regulation of extracellular matrix assembly	2	*AGT*	0.97		7.61E+01	1.41
GOTERM_MF	GO:0004021	L-alanine:2-oxoglutarate aminotransferase activity	2	*GPT*	0.97		7.01E+01	1.41
GOTERM_MF	GO:0004021	L-alanine:2-oxoglutarate aminotransferase	2	*GPT2*	0.54		7.01E+01	1.41

		activity					
GOTERM_MF	GO:0030250	guanylate cyclase activator activity	2	*GUCA2A*	1.06	7.01E+01	1.41
GOTERM_MF	GO:0030250	guanylate cyclase activator activity	2	*GUCA2B*	0.65	7.01E+01	1.41
GOTERM_BP	GO:0032720	negative regulation of tumor necrosis factor production	5	*AXL*	0.52	4.63E+01	1.34
GOTERM_BP	GO:0032720	negative regulation of tumor necrosis factor production	5	*ACP5*	0.67	4.63E+01	1.34
GOTERM_BP	GO:0032720	negative regulation of tumor necrosis factor production	5	*RARA*	0.54	4.63E+01	1.34
GOTERM_BP	GO:0032720	negative regulation of tumor necrosis factor production	5	*FOXP3*	1.07	4.63E+01	1.34
GOTERM_BP	GO:0032720	negative regulation of tumor necrosis factor production	5	*IL10*	-1.20	4.63E+01	1.34
GOTERM_BP	GO:0001889	liver development	5	*NOTCH1*	0.59	6.02E+01	1.34
GOTERM_BP	GO:0001889	liver development	5	*HNF1A*	0.50	6.02E+01	1.34
GOTERM_BP	GO:0001889	liver development	5	*JUN*	0.57	6.02E+01	1.34
GOTERM_BP	GO:0001889	liver development	5	*ADA*	-1.00	6.02E+01	1.34
GOTERM_BP	GO:0001889	liver development	5	*CITED2*	0.55	6.02E+01	1.34
KEGG_PATHWAY	bta00620	Pyruvate metabolism	5	*ME2*	0.86	6.65E+01	1.34
KEGG_PATHWAY	bta00620	Pyruvate metabolism	5	*PKLR*	0.83	6.65E+01	1.34
KEGG_PATHWAY	bta00620	Pyruvate metabolism	5	*LDHD*	1.10	6.65E+01	1.34
KEGG_PATHWAY	bta00620	Pyruvate metabolism	5	*ACAT1*	-0.64	6.65E+01	1.34
KEGG_PATHWAY	bta00620	Pyruvate metabolism	5	*PCK1*	3.19	6.65E+01	1.34
GOTERM_MF	GO:0042802	identical protein binding	10	*PLK4*	-0.55	7.60E+01	1.26
GOTERM_MF	GO:0042802	identical protein binding	10	*ALAD*	0.88	7.60E+01	1.26

GOTERM_MF	GO:0042802	identical protein binding	10	*FBP1*	1.19	7.60E+01	1.26
GOTERM_MF	GO:0042802	identical protein binding	10	*PCNA*	-0.78	7.60E+01	1.26
GOTERM_MF	GO:0042802	identical protein binding	10	*SERPINA1*	1.78	7.60E+01	1.26
GOTERM_MF	GO:0042802	identical protein binding	10	*NPR2*	0.55	7.60E+01	1.26
GOTERM_MF	GO:0042802	identical protein binding	10	*KCNJ2*	0.59	7.60E+01	1.26
GOTERM_MF	GO:0042802	identical protein binding	10	*ANXA4*	0.94	7.60E+01	1.26
GOTERM_MF	GO:0042802	identical protein binding	10	*S100A2*	-1.60	7.60E+01	1.26
GOTERM_MF	GO:0042802	identical protein binding	10	*ANGPTL4*	0.94	7.60E+01	1.26
GOTERM_BP	GO:0000122	negative regulation of transcription from RNA polymerase II promoter	26	*EGR1*	0.74	4.66E+01	1.18
GOTERM_BP	GO:0000122	negative regulation of transcription from RNA polymerase II promoter	26	*MAF*	0.66	4.66E+01	1.18
GOTERM_BP	GO:0000122	negative regulation of transcription from RNA polymerase II promoter	26	*ZFP36*	0.56	4.66E+01	1.18
GOTERM_BP	GO:0000122	negative regulation of transcription from RNA polymerase II promoter	26	*EID1*	-0.56	4.66E+01	1.18
GOTERM_BP	GO:0000122	negative regulation of transcription from RNA polymerase II promoter	26	*HIST1H1E*	-0.54	4.66E+01	1.18
GOTERM_BP	GO:0000122	negative regulation of transcription from RNA polymerase II promoter	26	*HIST1H1D*	-0.55	4.66E+01	1.18
GOTERM_BP	GO:0000122	negative regulation of transcription from RNA polymerase II promoter	26	*EFNA1*	0.82	4.66E+01	1.18
GOTERM_BP	GO:0000122	negative regulation of transcription from RNA polymerase II promoter	26	*WFS1*	0.66	4.66E+01	1.18
GOTERM_BP	GO:0000122	negative regulation of transcription from RNA polymerase II promoter	26	*E2F8*	-0.71	4.66E+01	1.18

GOTERM_BP	GO:0000122	negative regulation of transcription from RNA polymerase II promoter	26	*KLF11*	1.28	4.66E+01	1.18
GOTERM_BP	GO:0000122	negative regulation of transcription from RNA polymerase II promoter	26	*TLE4*	0.64	4.66E+01	1.18
GOTERM_BP	GO:0000122	negative regulation of transcription from RNA polymerase II promoter	26	*PAX5*	-4.82	4.66E+01	1.18
GOTERM_BP	GO:0000122	negative regulation of transcription from RNA polymerase II promoter	26	*AURKB*	-0.69	4.66E+01	1.18
GOTERM_BP	GO:0000122	negative regulation of transcription from RNA polymerase II promoter	26	*FOXP3*	1.07	4.66E+01	1.18
GOTERM_BP	GO:0000122	negative regulation of transcription from RNA polymerase II promoter	26	*PKIA*	-1.54	4.66E+01	1.18
GOTERM_BP	GO:0000122	negative regulation of transcription from RNA polymerase II promoter	26	*LOC504599*	-0.79	4.66E+01	1.18
GOTERM_BP	GO:0000122	negative regulation of transcription from RNA polymerase II promoter	26	*HIC1*	0.68	4.66E+01	1.18
GOTERM_BP	GO:0000122	negative regulation of transcription from RNA polymerase II promoter	26	*PCGF2*	0.57	4.66E+01	1.18
GOTERM_BP	GO:0000122	negative regulation of transcription from RNA polymerase II promoter	26	*NR1D1*	0.79	4.66E+01	1.18
GOTERM_BP	GO:0000122	negative regulation of transcription from RNA polymerase II promoter	26	*JUN*	0.57	4.66E+01	1.18
GOTERM_BP	GO:0000122	negative regulation of transcription from RNA polymerase II promoter	26	*TXN*	-0.56	4.66E+01	1.18
GOTERM_BP	GO:0000122	negative regulation of transcription from RNA polymerase II promoter	26	*TRPV4*	0.76	4.66E+01	1.18

GOTERM_BP	GO:0000122	negative regulation of transcription from RNA polymerase II promoter	26	*RARA*	0.54	4.66E+01	1.18
GOTERM_BP	GO:0000122	negative regulation of transcription from RNA polymerase II promoter	26	*HSF4*	0.55	4.66E+01	1.18
GOTERM_BP	GO:0000122	negative regulation of transcription from RNA polymerase II promoter	26	*NR1H3*	0.63	4.66E+01	1.18
GOTERM_BP	GO:0000122	negative regulation of transcription from RNA polymerase II promoter	26	*DNAJB5*	-1.07	4.66E+01	1.18
GOTERM_BP	GO:0070328	triglyceride homeostasis	4	*LPL*	-1.37	3.55E+01	1.00
GOTERM_BP	GO:0070328	triglyceride homeostasis	4	*SESN2*	0.65	3.55E+01	1.00
GOTERM_BP	GO:0070328	triglyceride homeostasis	4	*NR1H3*	0.63	3.55E+01	1.00
GOTERM_BP	GO:0070328	triglyceride homeostasis	4	*ANGPTL4*	0.94	3.55E+01	1.00
GOTERM_BP	GO:0032078	negative regulation of endodeoxyribonuclease activity	1	*GZMA*	0.69	7.61E+01	1.00
GOTERM_BP	GO:0009749	response to glucose	4	*EGR1*	0.74	7.91E+01	1.00
GOTERM_BP	GO:0009749	response to glucose	4	*LPL*	-1.37	7.91E+01	1.00
GOTERM_BP	GO:0009749	response to glucose	4	*HNF1A*	0.50	7.91E+01	1.00
GOTERM_BP	GO:0009749	response to glucose	4	*SESN2*	0.65	7.91E+01	1.00
GOTERM_BP	GO:0072593	reactive oxygen species metabolic process	4	*CTGF*	0.57	8.20E+01	1.00
GOTERM_BP	GO:0072593	reactive oxygen species metabolic process	4	*PDK4*	0.96	8.20E+01	1.00
GOTERM_BP	GO:0072593	reactive oxygen species metabolic process	4	*SESN2*	0.65	8.20E+01	1.00
GOTERM_BP	GO:0072593	reactive oxygen species metabolic process	4	*LRRK2*	-0.58	8.20E+01	1.00

GOTERM_BP	GO:0007339	binding of sperm to zona pellucida	4	*CLGN*	-2.03	8.20E+01	1.00
GOTERM_BP	GO:0007339	binding of sperm to zona pellucida	4	*ARSA*	0.83	8.20E+01	1.00
GOTERM_BP	GO:0007339	binding of sperm to zona pellucida	4	*HSPA1A*	0.77	8.20E+01	1.00
GOTERM_BP	GO:0007339	binding of sperm to zona pellucida	4	*PCSK4*	0.60	8.20E+01	1.00
GOTERM_MF	GO:0005247	voltage-gated chloride channel activity	4	*CLCN1*	1.36	6.40E+00	1.00
GOTERM_MF	GO:0005247	voltage-gated chloride channel activity	4	*CLCN2*	0.53	6.40E+00	1.00
GOTERM_MF	GO:0005247	voltage-gated chloride channel activity	4	*ANO6*	1.01	6.40E+00	1.00
GOTERM_MF	GO:0005247	voltage-gated chloride channel activity	4	*CLCN4*	-1.55	6.40E+00	1.00
GOTERM_MF	GO:0015293	symporter activity	4	*SLC16A1*	-0.61	5.28E+01	1.00
GOTERM_MF	GO:0015293	symporter activity	4	*SLC25A22*	0.63	5.28E+01	1.00
GOTERM_MF	GO:0015293	symporter activity	4	*SLC24A1*	1.03	5.28E+01	1.00
GOTERM_MF	GO:0015293	symporter activity	4	*SLC1A1*	2.91	5.28E+01	1.00
KEGG_PATHWAY	bta00590	Arachidonic acid metabolism	9	*GGT5*	1.25	4.69E+00	1.00
KEGG_PATHWAY	bta00590	Arachidonic acid metabolism	9	*CYP2B6*	-3.43	4.69E+00	1.00
KEGG_PATHWAY	bta00590	Arachidonic acid metabolism	9	*GPX3*	1.04	4.69E+00	1.00
KEGG_PATHWAY	bta00590	Arachidonic acid metabolism	9	*GGT1*	1.51	4.69E+00	1.00
KEGG_PATHWAY	bta00590	Arachidonic acid metabolism	9	*ALOX5*	1.14	4.69E+00	1.00
KEGG_PATHWAY	bta00590	Arachidonic acid metabolism	9	*CYP4F2*	2.23	4.69E+00	1.00
KEGG_PATHWAY	bta00590	Arachidonic acid metabolism	9	*PLA2G3*	-1.76	4.69E+00	1.00
KEGG_PATHWAY	bta00590	Arachidonic acid metabolism	9	*PLA2G4B*	0.55	4.69E+00	1.00
KEGG_PATHWAY	bta00590	Arachidonic acid metabolism	9	*PLA2G5*	-2.10	4.69E+00	1.00
GOTERM_MF	GO:0005509	calcium ion binding	31	*CDK5R1*	0.72	6.94E+01	0.90

GOTERM_MF	GO:0005509	calcium ion binding	31	*CLSTN3*	0.74	6.94E+01	0.90
GOTERM_MF	GO:0005509	calcium ion binding	31	*DUOX2*	-1.59	6.94E+01	0.90
GOTERM_MF	GO:0005509	calcium ion binding	31	*CETN4*	-0.55	6.94E+01	0.90
GOTERM_MF	GO:0005509	calcium ion binding	31	*MYL9*	-0.86	6.94E+01	0.90
GOTERM_MF	GO:0005509	calcium ion binding	31	*RASGRP3*	-0.71	6.94E+01	0.90
GOTERM_MF	GO:0005509	calcium ion binding	31	*RASGRP4*	0.52	6.94E+01	0.90
GOTERM_MF	GO:0005509	calcium ion binding	31	*SLC24A1*	1.03	6.94E+01	0.90
GOTERM_MF	GO:0005509	calcium ion binding	31	*OIT3*	1.02	6.94E+01	0.90
GOTERM_MF	GO:0005509	calcium ion binding	31	*PLCD1*	0.78	6.94E+01	0.90
GOTERM_MF	GO:0005509	calcium ion binding	31	*AGRN*	0.59	6.94E+01	0.90
GOTERM_MF	GO:0005509	calcium ion binding	31	*CDH26*	-1.69	6.94E+01	0.90
GOTERM_MF	GO:0005509	calcium ion binding	31	*EHD3*	-2.45	6.94E+01	0.90
GOTERM_MF	GO:0005509	calcium ion binding	31	*IHH*	0.71	6.94E+01	0.90
GOTERM_MF	GO:0005509	calcium ion binding	31	*VWA2*	0.60	6.94E+01	0.90
GOTERM_MF	GO:0005509	calcium ion binding	31	*S100A2*	-1.60	6.94E+01	0.90
GOTERM_MF	GO:0005509	calcium ion binding	31	*CAPSL*	-1.18	6.94E+01	0.90
GOTERM_MF	GO:0005509	calcium ion binding	31	*SCUBE1*	0.82	6.94E+01	0.90
GOTERM_MF	GO:0005509	calcium ion binding	31	*MMP19*	0.69	6.94E+01	0.90
GOTERM_MF	GO:0005509	calcium ion binding	31	*ACTN2*	-1.05	6.94E+01	0.90
GOTERM_MF	GO:0005509	calcium ion binding	31	*ANXA4*	0.94	6.94E+01	0.90
GOTERM_MF	GO:0005509	calcium ion binding	31	*NOTCH1*	0.59	6.94E+01	0.90
GOTERM_MF	GO:0005509	calcium ion binding	31	*CLGN*	-2.03	6.94E+01	0.90
GOTERM_MF	GO:0005509	calcium ion binding	31	*LRP1*	0.50	6.94E+01	0.90
GOTERM_MF	GO:0005509	calcium ion binding	31	*SNED1*	0.69	6.94E+01	0.90
GOTERM_MF	GO:0005509	calcium ion binding	31	*CAPN11*	0.75	6.94E+01	0.90

GOTERM_MF	GO:0005509	calcium ion binding	31	*ARSA*	0.83	6.94E+01	0.90
GOTERM_MF	GO:0005509	calcium ion binding	31	*LRP8*	-1.02	6.94E+01	0.90
GOTERM_MF	GO:0005509	calcium ion binding	31	*PROS1*	0.78	6.94E+01	0.90
GOTERM_MF	GO:0005509	calcium ion binding	31	*PLA2G5*	-2.10	6.94E+01	0.90
GOTERM_MF	GO:0005509	calcium ion binding	31	*MELK*	-0.69	6.94E+01	0.90
GOTERM_BP	GO:0071222	cellular response to lipopolysaccharide	8	*ZFP36*	0.56	3.31E+01	0.71
GOTERM_BP	GO:0071222	cellular response to lipopolysaccharide	8	*HMGB2*	-0.59	3.31E+01	0.71
GOTERM_BP	GO:0071222	cellular response to lipopolysaccharide	8	*NR1D1*	0.79	3.31E+01	0.71
GOTERM_BP	GO:0071222	cellular response to lipopolysaccharide	8	*AXL*	0.52	3.31E+01	0.71
GOTERM_BP	GO:0071222	cellular response to lipopolysaccharide	8	*RARA*	0.54	3.31E+01	0.71
GOTERM_BP	GO:0071222	cellular response to lipopolysaccharide	8	*CD180*	-3.33	3.31E+01	0.71
GOTERM_BP	GO:0071222	cellular response to lipopolysaccharide	8	*IL10*	-1.20	3.31E+01	0.71
GOTERM_BP	GO:0071222	cellular response to lipopolysaccharide	8	*NR1H3*	0.63	3.31E+01	0.71
KEGG_PATHWAY	bta03320	PPAR signaling pathway	8	*LPL*	-1.37	3.38E+01	0.71
KEGG_PATHWAY	bta03320	PPAR signaling pathway	8	*SCD*	-1.14	3.38E+01	0.71
KEGG_PATHWAY	bta03320	PPAR signaling pathway	8	*AQP7*	2.24	3.38E+01	0.71
KEGG_PATHWAY	bta03320	PPAR signaling pathway	8	*ACSL3*	-0.93	3.38E+01	0.71
KEGG_PATHWAY	bta03320	PPAR signaling pathway	8	*CPT1A*	0.95	3.38E+01	0.71
KEGG_PATHWAY	bta03320	PPAR signaling pathway	8	*NR1H3*	0.63	3.38E+01	0.71

KEGG_PATHWAY	bta03320	PPAR signaling pathway	8	*PCK1*	3.19	3.38E+01	0.71
KEGG_PATHWAY	bta03320	PPAR signaling pathway	8	*ANGPTL4*	0.94	3.38E+01	0.71
GOTERM_MF	GO:0008201	heparin binding	10	*F11*	-1.41	3.23E+01	0.63
GOTERM_MF	GO:0008201	heparin binding	10	*LPL*	-1.37	3.23E+01	0.63
GOTERM_MF	GO:0008201	heparin binding	10	*FMOD*	-1.24	3.23E+01	0.63
GOTERM_MF	GO:0008201	heparin binding	10	*CTGF*	0.57	3.23E+01	0.63
GOTERM_MF	GO:0008201	heparin binding	10	*CXCL13*	-3.12	3.23E+01	0.63
GOTERM_MF	GO:0008201	heparin binding	10	*FGFRL1*	0.51	3.23E+01	0.63
GOTERM_MF	GO:0008201	heparin binding	10	*APOH*	0.97	3.23E+01	0.63
GOTERM_MF	GO:0008201	heparin binding	10	*CHRD*	0.70	3.23E+01	0.63
GOTERM_MF	GO:0008201	heparin binding	10	*ANXA4*	0.94	3.23E+01	0.63
GOTERM_MF	GO:0008201	heparin binding	10	*APLP1*	0.51	3.23E+01	0.63
GOTERM_BP	GO:1903779	regulation of cardiac conduction	3	*AGT*	0.97	3.20E+01	0.58
GOTERM_BP	GO:1903779	regulation of cardiac conduction	3	*ACE2*	1.12	3.20E+01	0.58
GOTERM_BP	GO:1903779	regulation of cardiac conduction	3	*EHD3*	-2.45	3.20E+01	0.58
GOTERM_BP	GO:0061001	regulation of dendritic spine morphogenesis	3	*CDK5R1*	0.72	4.10E+01	0.58
GOTERM_BP	GO:0061001	regulation of dendritic spine morphogenesis	3	*LRRK2*	-0.58	4.10E+01	0.58
GOTERM_BP	GO:0061001	regulation of dendritic spine morphogenesis	3	*SHANK3*	0.54	4.10E+01	0.58
GOTERM_BP	GO:0019370	leukotriene biosynthetic process	3	*FCER1A*	-1.03	4.98E+01	0.58
GOTERM_BP	GO:0019370	leukotriene biosynthetic process	3	*GGT5*	1.25	4.98E+01	0.58
GOTERM_BP	GO:0019370	leukotriene biosynthetic process	3	*ALOX5*	1.14	4.98E+01	0.58
GOTERM_BP	GO:0032703	negative regulation of interleukin-2 production	3	*IL20RB*	-0.69	5.80E+01	0.58

GOTERM_BP	GO:0032703	negative regulation of interleukin-2 production	3	*FOXP3*	1.07	5.80E+01	0.58
GOTERM_BP	GO:0032703	negative regulation of interleukin-2 production	3	*VSIG4*	0.57	5.80E+01	0.58
GOTERM_BP	GO:0043277	apoptotic cell clearance	3	*HMGB1*	-0.52	6.54E+01	0.58
GOTERM_BP	GO:0043277	apoptotic cell clearance	3	*AXL*	0.52	6.54E+01	0.58
GOTERM_BP	GO:0043277	apoptotic cell clearance	3	*RARA*	0.54	6.54E+01	0.58
GOTERM_BP	GO:2000811	negative regulation of anoikis	3	*NOTCH1*	0.59	7.19E+01	0.58
GOTERM_BP	GO:2000811	negative regulation of anoikis	3	*PDK4*	0.96	7.19E+01	0.58
GOTERM_BP	GO:2000811	negative regulation of anoikis	3	*NTRK2*	-1.48	7.19E+01	0.58
GOTERM_BP	GO:0031668	cellular response to extracellular stimulus	3	*FOS*	1.03	8.23E+01	0.58
GOTERM_BP	GO:0031668	cellular response to extracellular stimulus	3	*SFRP2*	-1.05	8.23E+01	0.58
GOTERM_BP	GO:0031668	cellular response to extracellular stimulus	3	*AXL*	0.52	8.23E+01	0.58
GOTERM_MF	GO:0031404	chloride ion binding	3	*CLCN1*	1.36	5.15E+01	0.58
GOTERM_MF	GO:0031404	chloride ion binding	3	*CLCN2*	0.53	5.15E+01	0.58
GOTERM_MF	GO:0031404	chloride ion binding	3	*CLCN4*	-1.55	5.15E+01	0.58
GOTERM_BP	GO:0032689	negative regulation of interferon-gamma production	5	*IL20RB*	-0.69	1.05E+01	0.45
GOTERM_BP	GO:0032689	negative regulation of interferon-gamma production	5	*AXL*	0.52	1.05E+01	0.45
GOTERM_BP	GO:0032689	negative regulation of interferon-gamma production	5	*RARA*	0.54	1.05E+01	0.45
GOTERM_BP	GO:0032689	negative regulation of interferon-gamma production	5	*FOXP3*	1.07	1.05E+01	0.45

GOTERM_BP	GO:0032689	negative regulation of interferon-gamma production	5	*IL10*	-1.20	1.05E+01	0.45
KEGG_PATHWAY	bta01200	Carbon metabolism	11	*ME2*	0.86	2.25E+01	0.30
KEGG_PATHWAY	bta01200	Carbon metabolism	11	*PKLR*	0.83	2.25E+01	0.30
KEGG_PATHWAY	bta01200	Carbon metabolism	11	*ALDOB*	0.68	2.25E+01	0.30
KEGG_PATHWAY	bta01200	Carbon metabolism	11	*PHGDH*	-0.59	2.25E+01	0.30
KEGG_PATHWAY	bta01200	Carbon metabolism	11	*FBP1*	1.19	2.25E+01	0.30
KEGG_PATHWAY	bta01200	Carbon metabolism	11	*GPT*	0.97	2.25E+01	0.30
KEGG_PATHWAY	bta01200	Carbon metabolism	11	*TKTL2*	-1.43	2.25E+01	0.30
KEGG_PATHWAY	bta01200	Carbon metabolism	11	*PSAT1*	-1.22	2.25E+01	0.30
KEGG_PATHWAY	bta01200	Carbon metabolism	11	*ACAT1*	-0.64	2.25E+01	0.30
KEGG_PATHWAY	bta01200	Carbon metabolism	11	*GPT2*	0.54	2.25E+01	0.30
KEGG_PATHWAY	bta01200	Carbon metabolism	11	*GLDC*	-1.18	2.25E+01	0.30
GOTERM_MF	GO:0016705	oxidoreductase activity acting on paired donors，with incorporation or reduction of molecular oxygen	4	*CYP4F2*	2.23	4.32E+01	0.00
GOTERM_MF	GO:0016705	oxidoreductase activity acting on paired donors，with incorporation or reduction of molecular oxygen	4	*LOC509506*	0.76	4.32E+01	0.00
GOTERM_MF	GO:0016705	oxidoreductase activity acting on paired donors，with incorporation or reduction of molecular oxygen	4	*CYP2R1*	-0.65	4.32E+01	0.00
GOTERM_MF	GO:0016705	oxidoreductase activity acting on paired donors，with incorporation or reduction of molecular oxygen	4	*CYP2B6*	-3.43	4.32E+01	0.00
GOTERM_BP	GO:1902476	chloride transmembrane transport	8	*CLCN1*	1.36	8.80E+00	0.00

GOTERM_BP	GO:1902476	chloride transmembrane transport	8	*SLC26A6*	1.78	8.80E+00	0.00
GOTERM_BP	GO:1902476	chloride transmembrane transport	8	*CLCN2*	0.53	8.80E+00	0.00
GOTERM_BP	GO:1902476	chloride transmembrane transport	8	*GABRB2*	-0.75	8.80E+00	0.00
GOTERM_BP	GO:1902476	chloride transmembrane transport	8	*CFTR*	-0.69	8.80E+00	0.00
GOTERM_BP	GO:1902476	chloride transmembrane transport	8	*ANO4*	-1.01	8.80E+00	0.00
GOTERM_BP	GO:1902476	chloride transmembrane transport	8	*CLCN4*	-1.55	8.80E+00	0.00
GOTERM_BP	GO:1902476	chloride transmembrane transport	8	*BEST4*	0.78	8.80E+00	0.00
GOTERM_BP	GO:0035879	plasma membrane lactate transport	4	*SLC16A3*	0.65	1.06E+01	0.00
GOTERM_BP	GO:0035879	plasma membrane lactate transport	4	*SLC16A5*	0.64	1.06E+01	0.00
GOTERM_BP	GO:0035879	plasma membrane lactate transport	4	*SLC16A1*	-0.61	1.06E+01	0.00
GOTERM_BP	GO:0035879	plasma membrane lactate transport	4	*SLC16A7*	-0.56	1.06E+01	0.00
GOTERM_BP	GO:0051289	protein homotetramerization	6	*CTH*	-1.03	4.00E+01	0.00
GOTERM_BP	GO:0051289	protein homotetramerization	6	*GBP5*	-0.70	4.00E+01	0.00
GOTERM_BP	GO:0051289	protein homotetramerization	6	*GPX3*	1.04	4.00E+01	0.00
GOTERM_BP	GO:0051289	protein homotetramerization	6	*FBP1*	1.19	4.00E+01	0.00
GOTERM_BP	GO:0051289	protein homotetramerization	6	*ACTN2*	-1.05	4.00E+01	0.00
GOTERM_BP	GO:0051289	protein homotetramerization	6	*KCNJ2*	0.59	4.00E+01	0.00
GOTERM_BP	GO:0019344	cysteine biosynthetic process	2	*CTH*	-1.03	7.61E+01	0.00
GOTERM_BP	GO:0019344	cysteine biosynthetic process	2	*GGT1*	1.51	7.61E+01	0.00
GOTERM_BP	GO:0071287	cellular response to manganese ion	2	*LRRK2*	-0.58	7.61E+01	0.00
GOTERM_BP	GO:0071287	cellular response to manganese ion	2	*ATP13A2*	0.70	7.61E+01	0.00
GOTERM_BP	GO:0061589	calcium activated phosphatidylserine scrambling	2	*ANO4*	-1.01	7.61E+01	0.00
GOTERM_BP	GO:0061589	calcium activated phosphatidylserine scrambling	2	*ANO6*	1.01	7.61E+01	0.00

GOTERM_CC	GO:0031965	nuclear membrane	10	*NDC1*	-0.79	7.56E+01	0.00
GOTERM_CC	GO:0031965	nuclear membrane	10	*SLC16A3*	0.65	7.56E+01	0.00
GOTERM_CC	GO:0031965	nuclear membrane	10	*OSBPL6*	0.57	7.56E+01	0.00
GOTERM_CC	GO:0031965	nuclear membrane	10	*BRIP1*	-0.73	7.56E+01	0.00
GOTERM_CC	GO:0031965	nuclear membrane	10	*CERS4*	-1.50	7.56E+01	0.00
GOTERM_CC	GO:0031965	nuclear membrane	10	*FAM76B*	-0.60	7.56E+01	0.00
GOTERM_CC	GO:0031965	nuclear membrane	10	*ALOX5*	1.14	7.56E+01	0.00
GOTERM_CC	GO:0031965	nuclear membrane	10	*MX1*	0.67	7.56E+01	0.00
GOTERM_CC	GO:0031965	nuclear membrane	10	*ANXA4*	0.94	7.56E+01	0.00
GOTERM_CC	GO:0031965	nuclear membrane	10	*DMPK*	-0.87	7.56E+01	0.00
GOTERM_MF	GO:0008028	monocarboxylic acid transmembrane transporter activity	4	*SLC16A3*	0.65	2.88E+00	0.00
GOTERM_MF	GO:0008028	monocarboxylic acid transmembrane transporter activity	4	*SLC16A5*	0.64	2.88E+00	0.00
GOTERM_MF	GO:0008028	monocarboxylic acid transmembrane transporter activity	4	*SLC16A1*	-0.61	2.88E+00	0.00
GOTERM_MF	GO:0008028	monocarboxylic acid transmembrane transporter activity	4	*SLC16A7*	-0.56	2.88E+00	0.00
GOTERM_MF	GO:0015129	lactate transmembrane transporter activity	4	*SLC16A3*	0.65	1.15E+01	0.00
GOTERM_MF	GO:0015129	lactate transmembrane transporter activity	4	*SLC16A5*	0.64	1.15E+01	0.00
GOTERM_MF	GO:0015129	lactate transmembrane transporter activity	4	*SLC16A1*	-0.61	1.15E+01	0.00
GOTERM_MF	GO:0015129	lactate transmembrane transporter activity	4	*SLC16A7*	-0.56	1.15E+01	0.00

GOTERM_MF	GO:0020037	heme binding	10	*NR1D1*	0.79	2.81E+01	0.00
GOTERM_MF	GO:0020037	heme binding	10	*CYP51A1*	-1.00	2.81E+01	0.00
GOTERM_MF	GO:0020037	heme binding	10	*CYP2D14*	1.00	2.81E+01	0.00
GOTERM_MF	GO:0020037	heme binding	10	*CYP2B6*	-3.43	2.81E+01	0.00
GOTERM_MF	GO:0020037	heme binding	10	*FADS1*	-0.82	2.81E+01	0.00
GOTERM_MF	GO:0020037	heme binding	10	*DUOX2*	-1.59	2.81E+01	0.00
GOTERM_MF	GO:0020037	heme binding	10	*CYP2R1*	-0.65	2.81E+01	0.00
GOTERM_MF	GO:0020037	heme binding	10	*CYGB*	0.68	2.81E+01	0.00
GOTERM_MF	GO:0020037	heme binding	10	*LOC509506*	0.76	2.81E+01	0.00
GOTERM_MF	GO:0020037	heme binding	10	*CYP4F2*	2.23	2.81E+01	0.00
GOTERM_MF	GO:0004497	monooxygenase activity	4	*CYP2B6*	-3.43	4.32E+01	0.00
GOTERM_MF	GO:0004497	monooxygenase activity	4	*CYP2R1*	-0.65	4.32E+01	0.00
GOTERM_MF	GO:0004497	monooxygenase activity	4	*LOC509506*	0.76	4.32E+01	0.00
GOTERM_MF	GO:0004497	monooxygenase activity	4	*CYP4F2*	2.23	4.32E+01	0.00
GOTERM_MF	GO:0005506	iron ion binding	10	*CYP51A1*	-1.00	5.92E+01	0.00
GOTERM_MF	GO:0005506	iron ion binding	10	*CYP2D14*	1.00	5.92E+01	0.00
GOTERM_MF	GO:0005506	iron ion binding	10	*CYP2B6*	-3.43	5.92E+01	0.00
GOTERM_MF	GO:0005506	iron ion binding	10	*PLOD2*	-0.52	5.92E+01	0.00
GOTERM_MF	GO:0005506	iron ion binding	10	*SCD*	-1.14	5.92E+01	0.00
GOTERM_MF	GO:0005506	iron ion binding	10	*CYP2R1*	-0.65	5.92E+01	0.00
GOTERM_MF	GO:0005506	iron ion binding	10	*CYGB*	0.68	5.92E+01	0.00
GOTERM_MF	GO:0005506	iron ion binding	10	*LOC509506*	0.76	5.92E+01	0.00
GOTERM_MF	GO:0005506	iron ion binding	10	*ALOX5*	1.14	5.92E+01	0.00
GOTERM_MF	GO:0005506	iron ion binding	10	*CYP4F2*	2.23	5.92E+01	0.00
KEGG_PATHWAY	bta00480	Glutathione metabolism	6	*GGT5*	1.25	3.13E+01	0.00

KEGG_PATHWAY	bta00480	Glutathione metabolism	6	*RRM2*	-0.64	3.13E+01	0.00
KEGG_PATHWAY	bta00480	Glutathione metabolism	6	*GPX3*	1.04	3.13E+01	0.00
KEGG_PATHWAY	bta00480	Glutathione metabolism	6	*GGT1*	1.51	3.13E+01	0.00
KEGG_PATHWAY	bta00480	Glutathione metabolism	6	*RRM2B*	-0.64	3.13E+01	0.00
KEGG_PATHWAY	bta00480	Glutathione metabolism	6	*GSTO1*	-1.12	3.13E+01	0.00
KEGG_PATHWAY	bta01100	Metabolic pathways	66	*ALAD*	0.88	3.78E+01	0.00
KEGG_PATHWAY	bta01100	Metabolic pathways	66	*B3GALT5*	-1.58	3.78E+01	0.00
KEGG_PATHWAY	bta01100	Metabolic pathways	66	*CNDP2*	0.68	3.78E+01	0.00
KEGG_PATHWAY	bta01100	Metabolic pathways	66	*DTYMK*	-0.57	3.78E+01	0.00
KEGG_PATHWAY	bta01100	Metabolic pathways	66	*GGT1*	1.51	3.78E+01	0.00
KEGG_PATHWAY	bta01100	Metabolic pathways	66	*GLDC*	-1.18	3.78E+01	0.00
KEGG_PATHWAY	bta01100	Metabolic pathways	66	*XYLT1*	-0.97	3.78E+01	0.00
KEGG_PATHWAY	bta01100	Metabolic pathways	66	*MCCC1*	-0.50	3.78E+01	0.00
KEGG_PATHWAY	bta01100	Metabolic pathways	66	*MGC152010*	1.63	3.78E+01	0.00
KEGG_PATHWAY	bta01100	Metabolic pathways	66	*GPT2*	0.54	3.78E+01	0.00
KEGG_PATHWAY	bta01100	Metabolic pathways	66	*SARDH*	0.53	3.78E+01	0.00
KEGG_PATHWAY	bta01100	Metabolic pathways	66	*ATP6V0D2*	-1.14	3.78E+01	0.00
KEGG_PATHWAY	bta01100	Metabolic pathways	66	*NMNAT2*	0.67	3.78E+01	0.00
KEGG_PATHWAY	bta01100	Metabolic pathways	66	*PGAP1*	-0.59	3.78E+01	0.00
KEGG_PATHWAY	bta01100	Metabolic pathways	66	*POLE*	-0.66	3.78E+01	0.00
KEGG_PATHWAY	bta01100	Metabolic pathways	66	*FBP1*	1.19	3.78E+01	0.00
KEGG_PATHWAY	bta01100	Metabolic pathways	66	*LPIN3*	0.53	3.78E+01	0.00
KEGG_PATHWAY	bta01100	Metabolic pathways	66	*NME2*	-0.53	3.78E+01	0.00
KEGG_PATHWAY	bta01100	Metabolic pathways	66	*CTH*	-1.03	3.78E+01	0.00
KEGG_PATHWAY	bta01100	Metabolic pathways	66	*MGAT3*	0.57	3.78E+01	0.00

KEGG_PATHWAY	bta01100	Metabolic pathways	66	*RRM2*	-0.64	3.78E+01	0.00
KEGG_PATHWAY	bta01100	Metabolic pathways	66	*ADK*	-0.63	3.78E+01	0.00
KEGG_PATHWAY	bta01100	Metabolic pathways	66	*PKLR*	0.83	3.78E+01	0.00
KEGG_PATHWAY	bta01100	Metabolic pathways	66	*MGAM*	0.88	3.78E+01	0.00
KEGG_PATHWAY	bta01100	Metabolic pathways	66	*PLA2G3*	-1.76	3.78E+01	0.00
KEGG_PATHWAY	bta01100	Metabolic pathways	66	*DEGS2*	0.68	3.78E+01	0.00
KEGG_PATHWAY	bta01100	Metabolic pathways	66	*PLA2G5*	-2.10	3.78E+01	0.00
KEGG_PATHWAY	bta01100	Metabolic pathways	66	*PRODH*	0.84	3.78E+01	0.00
KEGG_PATHWAY	bta01100	Metabolic pathways	66	*BCAT1*	-1.74	3.78E+01	0.00
KEGG_PATHWAY	bta01100	Metabolic pathways	66	*MGAT5B*	1.10	3.78E+01	0.00
KEGG_PATHWAY	bta01100	Metabolic pathways	66	*CYP2B6*	-3.43	3.78E+01	0.00
KEGG_PATHWAY	bta01100	Metabolic pathways	66	*CYP51A1*	-1.00	3.78E+01	0.00
KEGG_PATHWAY	bta01100	Metabolic pathways	66	*ALDOB*	0.68	3.78E+01	0.00
KEGG_PATHWAY	bta01100	Metabolic pathways	66	*DCK*	-0.74	3.78E+01	0.00
KEGG_PATHWAY	bta01100	Metabolic pathways	66	*CERS4*	-1.50	3.78E+01	0.00
KEGG_PATHWAY	bta01100	Metabolic pathways	66	*TKTL2*	-1.43	3.78E+01	0.00
KEGG_PATHWAY	bta01100	Metabolic pathways	66	*RRM2B*	-0.64	3.78E+01	0.00
KEGG_PATHWAY	bta01100	Metabolic pathways	66	*ACAT1*	-0.64	3.78E+01	0.00
KEGG_PATHWAY	bta01100	Metabolic pathways	66	*PIPOX*	0.81	3.78E+01	0.00
KEGG_PATHWAY	bta01100	Metabolic pathways	66	*ADA*	-1.00	3.78E+01	0.00
KEGG_PATHWAY	bta01100	Metabolic pathways	66	*ISYNA1*	0.61	3.78E+01	0.00
KEGG_PATHWAY	bta01100	Metabolic pathways	66	*ARG2*	-1.26	3.78E+01	0.00
KEGG_PATHWAY	bta01100	Metabolic pathways	66	*FUT1*	1.72	3.78E+01	0.00
KEGG_PATHWAY	bta01100	Metabolic pathways	66	*PLCD1*	0.78	3.78E+01	0.00
KEGG_PATHWAY	bta01100	Metabolic pathways	66	*PAPSS2*	2.51	3.78E+01	0.00

KEGG_PATHWAY	bta01100	Metabolic pathways	66	*ACSL3*	-0.93	3.78E+01	0.00
KEGG_PATHWAY	bta01100	Metabolic pathways	66	*MAOB*	1.08	3.78E+01	0.00
KEGG_PATHWAY	bta01100	Metabolic pathways	66	*AMACR*	-1.22	3.78E+01	0.00
KEGG_PATHWAY	bta01100	Metabolic pathways	66	*UAP1L1*	0.57	3.78E+01	0.00
KEGG_PATHWAY	bta01100	Metabolic pathways	66	*AMPD3*	0.63	3.78E+01	0.00
KEGG_PATHWAY	bta01100	Metabolic pathways	66	*PCK1*	3.19	3.78E+01	0.00
KEGG_PATHWAY	bta01100	Metabolic pathways	66	*GGT5*	1.25	3.78E+01	0.00
KEGG_PATHWAY	bta01100	Metabolic pathways	66	*DHFR*	-0.66	3.78E+01	0.00
KEGG_PATHWAY	bta01100	Metabolic pathways	66	*PYGM*	-1.11	3.78E+01	0.00
KEGG_PATHWAY	bta01100	Metabolic pathways	66	*MLYCD*	0.51	3.78E+01	0.00
KEGG_PATHWAY	bta01100	Metabolic pathways	66	*PHGDH*	-0.59	3.78E+01	0.00
KEGG_PATHWAY	bta01100	Metabolic pathways	66	*GPT*	0.97	3.78E+01	0.00
KEGG_PATHWAY	bta01100	Metabolic pathways	66	*CYP2R1*	-0.65	3.78E+01	0.00
KEGG_PATHWAY	bta01100	Metabolic pathways	66	*ALOX5*	1.14	3.78E+01	0.00
KEGG_PATHWAY	bta01100	Metabolic pathways	66	*AHCYL2*	1.66	3.78E+01	0.00
KEGG_PATHWAY	bta01100	Metabolic pathways	66	*CYP4F2*	2.23	3.78E+01	0.00
KEGG_PATHWAY	bta01100	Metabolic pathways	66	*PSAT1*	-1.22	3.78E+01	0.00
KEGG_PATHWAY	bta01100	Metabolic pathways	66	*PLA2G4B*	0.55	3.78E+01	0.00
KEGG_PATHWAY	bta01100	Metabolic pathways	66	*PAICS*	-0.63	3.78E+01	0.00
KEGG_PATHWAY	bta01100	Metabolic pathways	66	*SMPD3*	0.91	3.78E+01	0.00
KEGG_PATHWAY	bta01100	Metabolic pathways	66	*DUT*	-0.65	3.78E+01	0.00
GOTERM_CC	GO:0070062	extracellular exosome	114	*C29H11ORF54*	-0.59	4.79E+00	-0.19
GOTERM_CC	GO:0070062	extracellular exosome	114	*RARRES2*	0.59	4.79E+00	-0.19
GOTERM_CC	GO:0070062	extracellular exosome	114	*ALAD*	0.88	4.79E+00	-0.19
GOTERM_CC	GO:0070062	extracellular exosome	114	*TSPAN1*	-2.31	4.79E+00	-0.19

GOTERM_CC	GO:0070062	extracellular exosome	114	*GABRB2*	-0.75	4.79E+00	-0.19
GOTERM_CC	GO:0070062	extracellular exosome	114	*EFNA1*	0.82	4.79E+00	-0.19
GOTERM_CC	GO:0070062	extracellular exosome	114	*CNDP2*	0.68	4.79E+00	-0.19
GOTERM_CC	GO:0070062	extracellular exosome	114	*MMP9*	1.04	4.79E+00	-0.19
GOTERM_CC	GO:0070062	extracellular exosome	114	*SLC7A8*	2.26	4.79E+00	-0.19
GOTERM_CC	GO:0070062	extracellular exosome	114	*KIFC3*	0.77	4.79E+00	-0.19
GOTERM_CC	GO:0070062	extracellular exosome	114	*ACTG2*	-1.39	4.79E+00	-0.19
GOTERM_CC	GO:0070062	extracellular exosome	114	*SLC16A1*	-0.61	4.79E+00	-0.19
GOTERM_CC	GO:0070062	extracellular exosome	114	*HIST1H2BN*	-0.72	4.79E+00	-0.19
GOTERM_CC	GO:0070062	extracellular exosome	114	*PLOD2*	-0.52	4.79E+00	-0.19
GOTERM_CC	GO:0070062	extracellular exosome	114	*MS4A1*	-3.70	4.79E+00	-0.19
GOTERM_CC	GO:0070062	extracellular exosome	114	*H2AFZ*	-0.60	4.79E+00	-0.19
GOTERM_CC	GO:0070062	extracellular exosome	114	*GUCA2A*	1.06	4.79E+00	-0.19
GOTERM_CC	GO:0070062	extracellular exosome	114	*SLC4A4*	1.24	4.79E+00	-0.19
GOTERM_CC	GO:0070062	extracellular exosome	114	*GUCA2B*	0.65	4.79E+00	-0.19
GOTERM_CC	GO:0070062	extracellular exosome	114	*NQO1*	-0.78	4.79E+00	-0.19
GOTERM_CC	GO:0070062	extracellular exosome	114	*PI15*	-1.34	4.79E+00	-0.19
GOTERM_CC	GO:0070062	extracellular exosome	114	*F11*	-1.41	4.79E+00	-0.19
GOTERM_CC	GO:0070062	extracellular exosome	114	*FBP1*	1.19	4.79E+00	-0.19
GOTERM_CC	GO:0070062	extracellular exosome	114	*ACTN2*	-1.05	4.79E+00	-0.19
GOTERM_CC	GO:0070062	extracellular exosome	114	*CD40*	-0.71	4.79E+00	-0.19
GOTERM_CC	GO:0070062	extracellular exosome	114	*NAPSA*	-2.03	4.79E+00	-0.19
GOTERM_CC	GO:0070062	extracellular exosome	114	*NEBL*	-1.69	4.79E+00	-0.19
GOTERM_CC	GO:0070062	extracellular exosome	114	*RETN*	0.70	4.79E+00	-0.19
GOTERM_CC	GO:0070062	extracellular exosome	114	*RAB17*	0.91	4.79E+00	-0.19

GOTERM_CC	GO:0070062	extracellular exosome	114	*MGAM*	0.88	4.79E+00	-0.19
GOTERM_CC	GO:0070062	extracellular exosome	114	*RAB15*	0.72	4.79E+00	-0.19
GOTERM_CC	GO:0070062	extracellular exosome	114	*STMN1*	-1.40	4.79E+00	-0.19
GOTERM_CC	GO:0070062	extracellular exosome	114	*LRRK2*	-0.58	4.79E+00	-0.19
GOTERM_CC	GO:0070062	extracellular exosome	114	*VSIG4*	0.57	4.79E+00	-0.19
GOTERM_CC	GO:0070062	extracellular exosome	114	*SH3GL2*	0.95	4.79E+00	-0.19
GOTERM_CC	GO:0070062	extracellular exosome	114	*WFDC2*	0.83	4.79E+00	-0.19
GOTERM_CC	GO:0070062	extracellular exosome	114	*OLFM4*	1.13	4.79E+00	-0.19
GOTERM_CC	GO:0070062	extracellular exosome	114	*ALDOB*	0.68	4.79E+00	-0.19
GOTERM_CC	GO:0070062	extracellular exosome	114	*ACP5*	0.67	4.79E+00	-0.19
GOTERM_CC	GO:0070062	extracellular exosome	114	*RRM2B*	-0.64	4.79E+00	-0.19
GOTERM_CC	GO:0070062	extracellular exosome	114	*ACAT1*	-0.64	4.79E+00	-0.19
GOTERM_CC	GO:0070062	extracellular exosome	114	*FGL1*	-1.02	4.79E+00	-0.19
GOTERM_CC	GO:0070062	extracellular exosome	114	*AGRN*	0.59	4.79E+00	-0.19
GOTERM_CC	GO:0070062	extracellular exosome	114	*LOC505183*	-0.88	4.79E+00	-0.19
GOTERM_CC	GO:0070062	extracellular exosome	114	*CNKSR2*	-1.27	4.79E+00	-0.19
GOTERM_CC	GO:0070062	extracellular exosome	114	*LPL*	-1.37	4.79E+00	-0.19
GOTERM_CC	GO:0070062	extracellular exosome	114	*SPTBN5*	0.69	4.79E+00	-0.19
GOTERM_CC	GO:0070062	extracellular exosome	114	*AKR1E2*	-1.17	4.79E+00	-0.19
GOTERM_CC	GO:0070062	extracellular exosome	114	*FBXO2*	0.57	4.79E+00	-0.19
GOTERM_CC	GO:0070062	extracellular exosome	114	*SERPINI1*	-0.61	4.79E+00	-0.19
GOTERM_CC	GO:0070062	extracellular exosome	114	*LAMA2*	0.56	4.79E+00	-0.19
GOTERM_CC	GO:0070062	extracellular exosome	114	*TMEM8A*	0.59	4.79E+00	-0.19
GOTERM_CC	GO:0070062	extracellular exosome	114	*HEBP2*	0.95	4.79E+00	-0.19
GOTERM_CC	GO:0070062	extracellular exosome	114	*MYH11*	-0.87	4.79E+00	-0.19

GOTERM_CC	GO:0070062	extracellular exosome	114	*PCNA*	-0.78	4.79E+00	-0.19
GOTERM_CC	GO:0070062	extracellular exosome	114	*CD79B*	-3.11	4.79E+00	-0.19
GOTERM_CC	GO:0070062	extracellular exosome	114	*PDZK1*	1.03	4.79E+00	-0.19
GOTERM_CC	GO:0070062	extracellular exosome	114	*MYLK*	-0.86	4.79E+00	-0.19
GOTERM_CC	GO:0070062	extracellular exosome	114	*CLSTN3*	0.74	4.79E+00	-0.19
GOTERM_CC	GO:0070062	extracellular exosome	114	*FAM3B*	0.94	4.79E+00	-0.19
GOTERM_CC	GO:0070062	extracellular exosome	114	*SUSD2*	2.68	4.79E+00	-0.19
GOTERM_CC	GO:0070062	extracellular exosome	114	*DUOX2*	-1.59	4.79E+00	-0.19
GOTERM_CC	GO:0070062	extracellular exosome	114	*GPX3*	1.04	4.79E+00	-0.19
GOTERM_CC	GO:0070062	extracellular exosome	114	*CHST14*	0.53	4.79E+00	-0.19
GOTERM_CC	GO:0070062	extracellular exosome	114	*SERPINA1*	1.78	4.79E+00	-0.19
GOTERM_CC	GO:0070062	extracellular exosome	114	*SLC22A5*	0.51	4.79E+00	-0.19
GOTERM_CC	GO:0070062	extracellular exosome	114	*ANO6*	1.01	4.79E+00	-0.19
GOTERM_CC	GO:0070062	extracellular exosome	114	*ATP6V0D2*	-1.14	4.79E+00	-0.19
GOTERM_CC	GO:0070062	extracellular exosome	114	*SLC1A1*	2.91	4.79E+00	-0.19
GOTERM_CC	GO:0070062	extracellular exosome	114	*VWA2*	0.60	4.79E+00	-0.19
GOTERM_CC	GO:0070062	extracellular exosome	114	*EPN3*	-1.05	4.79E+00	-0.19
GOTERM_CC	GO:0070062	extracellular exosome	114	*CDK1*	-0.54	4.79E+00	-0.19
GOTERM_CC	GO:0070062	extracellular exosome	114	*HIST1H1E*	-0.54	4.79E+00	-0.19
GOTERM_CC	GO:0070062	extracellular exosome	114	*PEPD*	1.03	4.79E+00	-0.19
GOTERM_CC	GO:0070062	extracellular exosome	114	*GEN1*	-0.78	4.79E+00	-0.19
GOTERM_CC	GO:0070062	extracellular exosome	114	*MPP6*	-0.60	4.79E+00	-0.19
GOTERM_CC	GO:0070062	extracellular exosome	114	*CFTR*	-0.69	4.79E+00	-0.19
GOTERM_CC	GO:0070062	extracellular exosome	114	*LOC504599*	-0.79	4.79E+00	-0.19
GOTERM_CC	GO:0070062	extracellular exosome	114	*TARS*	-1.19	4.79E+00	-0.19

GOTERM_CC	GO:0070062	extracellular exosome	114	*CTH*	-1.03	4.79E+00	-0.19
GOTERM_CC	GO:0070062	extracellular exosome	114	*BTG2*	0.55	4.79E+00	-0.19
GOTERM_CC	GO:0070062	extracellular exosome	114	*CFL2*	-0.65	4.79E+00	-0.19
GOTERM_CC	GO:0070062	extracellular exosome	114	*PKLR*	0.83	4.79E+00	-0.19
GOTERM_CC	GO:0070062	extracellular exosome	114	*TXN*	-0.56	4.79E+00	-0.19
GOTERM_CC	GO:0070062	extracellular exosome	114	*LOC787465*	-0.71	4.79E+00	-0.19
GOTERM_CC	GO:0070062	extracellular exosome	114	*PROS1*	0.78	4.79E+00	-0.19
GOTERM_CC	GO:0070062	extracellular exosome	114	*FKBP5*	-0.50	4.79E+00	-0.19
GOTERM_CC	GO:0070062	extracellular exosome	114	*HIST1H2AG*	-0.84	4.79E+00	-0.19
GOTERM_CC	GO:0070062	extracellular exosome	114	*AGT*	0.97	4.79E+00	-0.19
GOTERM_CC	GO:0070062	extracellular exosome	114	*CD22*	-1.72	4.79E+00	-0.19
GOTERM_CC	GO:0070062	extracellular exosome	114	*PLCD1*	0.78	4.79E+00	-0.19
GOTERM_CC	GO:0070062	extracellular exosome	114	*COL8A1*	-1.92	4.79E+00	-0.19
GOTERM_CC	GO:0070062	extracellular exosome	114	*SCNN1A*	1.17	4.79E+00	-0.19
GOTERM_CC	GO:0070062	extracellular exosome	114	*FLRT2*	-0.52	4.79E+00	-0.19
GOTERM_CC	GO:0070062	extracellular exosome	114	*CR2*	-3.53	4.79E+00	-0.19
GOTERM_CC	GO:0070062	extracellular exosome	114	*HIST1H2BD*	-0.59	4.79E+00	-0.19
GOTERM_CC	GO:0070062	extracellular exosome	114	*PM20D1*	0.53	4.79E+00	-0.19
GOTERM_CC	GO:0070062	extracellular exosome	114	*AXL*	0.52	4.79E+00	-0.19
GOTERM_CC	GO:0070062	extracellular exosome	114	*KLK1*	0.80	4.79E+00	-0.19
GOTERM_CC	GO:0070062	extracellular exosome	114	*SMC2*	-0.71	4.79E+00	-0.19
GOTERM_CC	GO:0070062	extracellular exosome	114	*ANXA4*	0.94	4.79E+00	-0.19
GOTERM_CC	GO:0070062	extracellular exosome	114	*LOC616819*	-0.75	4.79E+00	-0.19
GOTERM_CC	GO:0070062	extracellular exosome	114	*XPNPEP2*	1.91	4.79E+00	-0.19
GOTERM_CC	GO:0070062	extracellular exosome	114	*PCK1*	3.19	4.79E+00	-0.19

GOTERM_CC	GO:0070062	extracellular exosome	114	*CD19*	-1.06	4.79E+00	-0.19
GOTERM_CC	GO:0070062	extracellular exosome	114	*SNED1*	0.69	4.79E+00	-0.19
GOTERM_CC	GO:0070062	extracellular exosome	114	*PYGM*	-1.11	4.79E+00	-0.19
GOTERM_CC	GO:0070062	extracellular exosome	114	*CAPG*	2.06	4.79E+00	-0.19
GOTERM_CC	GO:0070062	extracellular exosome	114	*ACE2*	1.12	4.79E+00	-0.19
GOTERM_CC	GO:0070062	extracellular exosome	114	*GPT*	0.97	4.79E+00	-0.19
GOTERM_CC	GO:0070062	extracellular exosome	114	*METRNL*	0.50	4.79E+00	-0.19
GOTERM_CC	GO:0070062	extracellular exosome	114	*HIST1H2AJ*	-1.05	4.79E+00	-0.19
GOTERM_CC	GO:0070062	extracellular exosome	114	*PSAT1*	-1.22	4.79E+00	-0.19
GOTERM_CC	GO:0070062	extracellular exosome	114	*PAICS*	-0.63	4.79E+00	-0.19
GOTERM_BP	GO:0006954	inflammatory response	17	*HMGB1*	-0.52	2.22E+01	-0.24
GOTERM_BP	GO:0006954	inflammatory response	17	*RARRES2*	0.59	2.22E+01	-0.24
GOTERM_BP	GO:0006954	inflammatory response	17	*F2RL1*	0.62	2.22E+01	-0.24
GOTERM_BP	GO:0006954	inflammatory response	17	*AXL*	0.52	2.22E+01	-0.24
GOTERM_BP	GO:0006954	inflammatory response	17	*TLR2*	-0.65	2.22E+01	-0.24
GOTERM_BP	GO:0006954	inflammatory response	17	*CCL19*	-1.10	2.22E+01	-0.24
GOTERM_BP	GO:0006954	inflammatory response	17	*CD40*	-0.71	2.22E+01	-0.24
GOTERM_BP	GO:0006954	inflammatory response	17	*CD180*	-3.33	2.22E+01	-0.24
GOTERM_BP	GO:0006954	inflammatory response	17	*IL10*	-1.20	2.22E+01	-0.24
GOTERM_BP	GO:0006954	inflammatory response	17	*CCL24*	2.14	2.22E+01	-0.24
GOTERM_BP	GO:0006954	inflammatory response	17	*S1PR3*	-0.66	2.22E+01	-0.24
GOTERM_BP	GO:0006954	inflammatory response	17	*GGT5*	1.25	2.22E+01	-0.24
GOTERM_BP	GO:0006954	inflammatory response	17	*CXCL13*	-3.12	2.22E+01	-0.24
GOTERM_BP	GO:0006954	inflammatory response	17	*LTB4R*	0.82	2.22E+01	-0.24
GOTERM_BP	GO:0006954	inflammatory response	17	*CCR3*	1.20	2.22E+01	-0.24

GOTERM_BP	GO:0006954	inflammatory response	17	*TICAM1*	0.91	2.22E+01	-0.24
GOTERM_BP	GO:0006954	inflammatory response	17	*TNFRSF18*	-0.63	2.22E+01	-0.24
KEGG_PATHWAY	bta00260	Glycine serine and threonine metabolism	7	*CTH*	-1.03	9.79E+00	-0.38
KEGG_PATHWAY	bta00260	Glycine serine and threonine metabolism	7	*MAOB*	1.08	9.79E+00	-0.38
KEGG_PATHWAY	bta00260	Glycine serine and threonine metabolism	7	*PHGDH*	-0.60	9.79E+00	-0.38
KEGG_PATHWAY	bta00260	Glycine serine and threonine metabolism	7	*PSAT1*	-1.22	9.79E+00	-0.38
KEGG_PATHWAY	bta00260	Glycine serine and threonine metabolism	7	*SARDH*	0.53	9.79E+00	-0.38
KEGG_PATHWAY	bta00260	Glycine serine and threonine metabolism	7	*PIPOX*	0.81	9.79E+00	-0.38
KEGG_PATHWAY	bta00260	Glycine serine and threonine metabolism	7	*GLDC*	-1.18	9.79E+00	-0.38
GOTERM_BP	GO:0008543	fibroblast growth factor receptor signaling pathway	7	*FGF19*	2.56	5.05E+00	-0.38
GOTERM_BP	GO:0008543	fibroblast growth factor receptor signaling pathway	7	*FLRT2*	-0.52	5.05E+00	-0.38
GOTERM_BP	GO:0008543	fibroblast growth factor receptor signaling pathway	7	*FGF7*	-0.87	5.05E+00	-0.38
GOTERM_BP	GO:0008543	fibroblast growth factor receptor signaling pathway	7	*CTGF*	0.57	5.05E+00	-0.38
GOTERM_BP	GO:0008543	fibroblast growth factor receptor signaling pathway	7	*FGF9*	0.78	5.05E+00	-0.38

GOTERM_BP	GO:0008543	fibroblast growth factor receptor signaling pathway	7	*FGF12*	-0.69	5.05E+00	-0.38
GOTERM_BP	GO:0008543	fibroblast growth factor receptor signaling pathway	7	*SHCBP1*	-0.64	5.05E+00	-0.38
GOTERM_BP	GO:0002244	hematopoietic progenitor cell differentiation	7	*ARL11*	-0.65	4.51E+01	-0.38
GOTERM_BP	GO:0002244	hematopoietic progenitor cell differentiation	7	*KCNAB2*	0.51	4.51E+01	-0.38
GOTERM_BP	GO:0002244	hematopoietic progenitor cell differentiation	7	*PTPRZ1*	-1.16	4.51E+01	-0.38
GOTERM_BP	GO:0002244	hematopoietic progenitor cell differentiation	7	*ANLN*	-0.53	4.51E+01	-0.38
GOTERM_BP	GO:0002244	hematopoietic progenitor cell differentiation	7	*TOP2A*	-0.76	4.51E+01	-0.38
GOTERM_BP	GO:0002244	hematopoietic progenitor cell differentiation	7	*SMPD3*	0.91	4.51E+01	-0.38
GOTERM_BP	GO:0002244	hematopoietic progenitor cell differentiation	7	*CITED2*	0.55	4.51E+01	-0.38
GOTERM_BP	GO:0034599	cellular response to oxidative stress	5	*FANCD2*	-0.91	8.02E+01	-0.45
GOTERM_BP	GO:0034599	cellular response to oxidative stress	5	*TXN*	-0.56	8.02E+01	-0.45
GOTERM_BP	GO:0034599	cellular response to oxidative stress	5	*SESN2*	0.65	8.02E+01	-0.45
GOTERM_BP	GO:0034599	cellular response to oxidative stress	5	*LRRK2*	-0.58	8.02E+01	-0.45
GOTERM_BP	GO:0034599	cellular response to oxidative stress	5	*ATP13A2*	0.70	8.02E+01	-0.45
KEGG_PATHWAY	bta01130	Biosynthesis of antibiotics	20	*BCAT1*	-1.74	1.89E+00	-0.45
KEGG_PATHWAY	bta01130	Biosynthesis of antibiotics	20	*CYP51A1*	-1.00	1.89E+00	-0.45
KEGG_PATHWAY	bta01130	Biosynthesis of antibiotics	20	*ALDOB*	0.68	1.89E+00	-0.45
KEGG_PATHWAY	bta01130	Biosynthesis of antibiotics	20	*FBP1*	1.19	1.89E+00	-0.45

KEGG_PATHWAY	bta01130	Biosynthesis of antibiotics	20	*TKTL2*	-1.43	1.89E+00	-0.45
KEGG_PATHWAY	bta01130	Biosynthesis of antibiotics	20	*UAP1L1*	0.57	1.89E+00	-0.45
KEGG_PATHWAY	bta01130	Biosynthesis of antibiotics	20	*AMPD3*	0.63	1.89E+00	-0.45
KEGG_PATHWAY	bta01130	Biosynthesis of antibiotics	20	*ACAT1*	-0.64	1.89E+00	-0.45
KEGG_PATHWAY	bta01130	Biosynthesis of antibiotics	20	*GLDC*	-1.18	1.89E+00	-0.45
KEGG_PATHWAY	bta01130	Biosynthesis of antibiotics	20	*PCK1*	3.19	1.89E+00	-0.45
KEGG_PATHWAY	bta01130	Biosynthesis of antibiotics	20	*ISYNA1*	0.61	1.89E+00	-0.45
KEGG_PATHWAY	bta01130	Biosynthesis of antibiotics	20	*NME2*	-0.53	1.89E+00	-0.45
KEGG_PATHWAY	bta01130	Biosynthesis of antibiotics	20	*CTH*	-1.03	1.89E+00	-0.45
KEGG_PATHWAY	bta01130	Biosynthesis of antibiotics	20	*ARG2*	-1.26	1.89E+00	-0.45
KEGG_PATHWAY	bta01130	Biosynthesis of antibiotics	20	*PKLR*	0.83	1.89E+00	-0.45
KEGG_PATHWAY	bta01130	Biosynthesis of antibiotics	20	*PHGDH*	-0.59	1.89E+00	-0.45
KEGG_PATHWAY	bta01130	Biosynthesis of antibiotics	20	*PSAT1*	-1.22	1.89E+00	-0.45
KEGG_PATHWAY	bta01130	Biosynthesis of antibiotics	20	*PAPSS2*	2.51	1.89E+00	-0.45
KEGG_PATHWAY	bta01130	Biosynthesis of antibiotics	20	*PAICS*	-0.63	1.89E+00	-0.45
KEGG_PATHWAY	bta01130	Biosynthesis of antibiotics	20	*PRODH*	0.84	1.89E+00	-0.45
GOTERM_BP	GO:0002437	inflammatory response to antigenic stimulus	3	*HMGB2*	-0.59	4.98E+01	-0.58
GOTERM_BP	GO:0002437	inflammatory response to antigenic stimulus	3	*NOTCH1*	0.59	4.98E+01	-0.58
GOTERM_BP	GO:0002437	inflammatory response to antigenic stimulus	3	*IL20RB*	-0.69	4.98E+01	-0.58
GOTERM_BP	GO:0032060	bleb assembly	3	*LPAR3*	-1.39	6.54E+01	-0.58
GOTERM_BP	GO:0032060	bleb assembly	3	*ANO6*	1.01	6.54E+01	-0.58
GOTERM_BP	GO:0032060	bleb assembly	3	*MYLK*	-0.86	6.54E+01	-0.58
GOTERM_BP	GO:0048566	embryonic digestive tract development	3	*RARRES2*	0.59	7.75E+01	-0.58

GOTERM_BP	GO:0048566	embryonic digestive tract development	3	*STRA6*	-1.92	7.75E+01	-0.58
GOTERM_BP	GO:0048566	embryonic digestive tract development	3	*ADA*	-1.00	7.75E+01	-0.58
GOTERM_BP	GO:0043065	positive regulation of apoptotic process	10	*NOTCH1*	0.59	5.42E+01	-0.63
GOTERM_BP	GO:0043065	positive regulation of apoptotic process	10	*SFRP2*	-1.05	5.42E+01	-0.63
GOTERM_BP	GO:0043065	positive regulation of apoptotic process	10	*GZMA*	0.69	5.42E+01	-0.63
GOTERM_BP	GO:0043065	positive regulation of apoptotic process	10	*BCL2A1*	-0.69	5.42E+01	-0.63
GOTERM_BP	GO:0043065	positive regulation of apoptotic process	10	*KLF11*	1.28	5.42E+01	-0.63
GOTERM_BP	GO:0043065	positive regulation of apoptotic process	10	*NR4A1*	1.03	5.42E+01	-0.63
GOTERM_BP	GO:0043065	positive regulation of apoptotic process	10	*TOP2A*	-0.76	5.42E+01	-0.63
GOTERM_BP	GO:0043065	positive regulation of apoptotic process	10	*ECT2*	-0.53	5.42E+01	-0.63
GOTERM_BP	GO:0043065	positive regulation of apoptotic process	10	*MELK*	-0.69	5.42E+01	-0.63
GOTERM_BP	GO:0043065	positive regulation of apoptotic process	10	*BARD1*	-0.68	5.42E+01	-0.63
KEGG_PATHWAY	bta01230	Biosynthesis of amino acids	10	*BCAT1*	-1.74	4.27E+00	-0.63
KEGG_PATHWAY	bta01230	Biosynthesis of amino acids	10	*CTH*	-1.03	4.27E+00	-0.63
KEGG_PATHWAY	bta01230	Biosynthesis of amino acids	10	*ARG2*	-1.26	4.27E+00	-0.63
KEGG_PATHWAY	bta01230	Biosynthesis of amino acids	10	*PKLR*	0.83	4.27E+00	-0.63
KEGG_PATHWAY	bta01230	Biosynthesis of amino acids	10	*ALDOB*	0.68	4.27E+00	-0.63
KEGG_PATHWAY	bta01230	Biosynthesis of amino acids	10	*PHGDH*	-0.59	4.27E+00	-0.63

KEGG_PATHWAY	bta01230	Biosynthesis of amino acids	10	*GPT*	0.97	4.27E+00	-0.63
KEGG_PATHWAY	bta01230	Biosynthesis of amino acids	10	*TKTL2*	-1.43	4.27E+00	-0.63
KEGG_PATHWAY	bta01230	Biosynthesis of amino acids	10	*PSAT1*	-1.22	4.27E+00	-0.63
KEGG_PATHWAY	bta01230	Biosynthesis of amino acids	10	*GPT2*	0.54	4.27E+00	-0.63
GOTERM_MF	GO:0031490	chromatin DNA binding	8	*HIST1H1E*	-0.54	5.70E+00	-0.71
GOTERM_MF	GO:0031490	chromatin DNA binding	8	*NOTCH1*	0.59	5.70E+00	-0.71
GOTERM_MF	GO:0031490	chromatin DNA binding	8	*HIST1H1D*	-0.55	5.70E+00	-0.71
GOTERM_MF	GO:0031490	chromatin DNA binding	8	*HIST1H1A*	-1.47	5.70E+00	-0.71
GOTERM_MF	GO:0031490	chromatin DNA binding	8	*H2AFZ*	-0.60	5.70E+00	-0.71
GOTERM_MF	GO:0031490	chromatin DNA binding	8	*RARA*	0.54	5.70E+00	-0.71
GOTERM_MF	GO:0031490	chromatin DNA binding	8	*GRHL1*	0.85	5.70E+00	-0.71
GOTERM_MF	GO:0031490	chromatin DNA binding	8	*APEX1*	-0.50	5.70E+00	-0.71
GOTERM_BP	GO:0006633	fatty acid biosynthetic process	6	*LPL*	-1.37	1.24E+01	-0.82
GOTERM_BP	GO:0006633	fatty acid biosynthetic process	6	*HNF1A*	0.50	1.24E+01	-0.82
GOTERM_BP	GO:0006633	fatty acid biosynthetic process	6	*MLYCD*	0.51	1.24E+01	-0.82
GOTERM_BP	GO:0006633	fatty acid biosynthetic process	6	*SCD*	-1.14	1.24E+01	-0.82
GOTERM_BP	GO:0006633	fatty acid biosynthetic process	6	*FADS3*	-1.47	1.24E+01	-0.82
GOTERM_BP	GO:0006633	fatty acid biosynthetic process	6	*BRCA1*	-0.56	1.24E+01	-0.82
GOTERM_BP	GO:0008584	male gonad development	6	*HMGB2*	-0.59	6.23E+01	-0.82
GOTERM_BP	GO:0008584	male gonad development	6	*SFRP2*	-1.05	6.23E+01	-0.82
GOTERM_BP	GO:0008584	male gonad development	6	*MSH2*	-0.81	6.23E+01	-0.82
GOTERM_BP	GO:0008584	male gonad development	6	*ASPM*	-0.68	6.23E+01	-0.82
GOTERM_BP	GO:0008584	male gonad development	6	*WNT2B*	0.58	6.23E+01	-0.82
GOTERM_BP	GO:0008584	male gonad development	6	*CITED2*	0.55	6.23E+01	-0.82
GOTERM_CC	GO:0000790	nuclear chromatin	13	*HIST1H2AC*	-0.59	2.53E+01	-0.83

GOTERM_CC	GO:0000790	nuclear chromatin	13	*HMGB1*	-0.52	2.53E+01	-0.83
GOTERM_CC	GO:0000790	nuclear chromatin	13	*HMGB2*	-0.59	2.53E+01	-0.83
GOTERM_CC	GO:0000790	nuclear chromatin	13	*HIST1H1E*	-0.54	2.53E+01	-0.83
GOTERM_CC	GO:0000790	nuclear chromatin	13	*NR1D1*	0.79	2.53E+01	-0.83
GOTERM_CC	GO:0000790	nuclear chromatin	13	*HIST1H2AG*	-0.84	2.53E+01	-0.83
GOTERM_CC	GO:0000790	nuclear chromatin	13	*NASP*	-0.51	2.53E+01	-0.83
GOTERM_CC	GO:0000790	nuclear chromatin	13	*JUN*	0.57	2.53E+01	-0.83
GOTERM_CC	GO:0000790	nuclear chromatin	13	*H2AFZ*	-0.60	2.53E+01	-0.83
GOTERM_CC	GO:0000790	nuclear chromatin	13	*HAT1*	-0.53	2.53E+01	-0.83
GOTERM_CC	GO:0000790	nuclear chromatin	13	*RARA*	0.54	2.53E+01	-0.83
GOTERM_CC	GO:0000790	nuclear chromatin	13	*ZNF385A*	0.63	2.53E+01	-0.83
GOTERM_CC	GO:0000790	nuclear chromatin	13	*CITED2*	0.55	2.53E+01	-0.83
GOTERM_MF	GO:0016491	oxidoreductase activity	11	*AKR1E2*	-1.17	1.53E+01	-0.90
GOTERM_MF	GO:0016491	oxidoreductase activity	11	*DHRS13*	0.74	1.53E+01	-0.90
GOTERM_MF	GO:0016491	oxidoreductase activity	11	*RRM2*	-0.64	1.53E+01	-0.90
GOTERM_MF	GO:0016491	oxidoreductase activity	11	*SCD*	-1.14	1.53E+01	-0.90
GOTERM_MF	GO:0016491	oxidoreductase activity	11	*FADS1*	-0.82	1.53E+01	-0.90
GOTERM_MF	GO:0016491	oxidoreductase activity	11	*MAOB*	1.08	1.53E+01	-0.90
GOTERM_MF	GO:0016491	oxidoreductase activity	11	*FADS3*	-1.47	1.53E+01	-0.90
GOTERM_MF	GO:0016491	oxidoreductase activity	11	*MCM3*	-0.67	1.53E+01	-0.90
GOTERM_MF	GO:0016491	oxidoreductase activity	11	*SARDH*	0.53	1.53E+01	-0.90
GOTERM_MF	GO:0016491	oxidoreductase activity	11	*APEX1*	-0.50	1.53E+01	-0.90
GOTERM_MF	GO:0016491	oxidoreductase activity	11	*DHRS7*	0.64	1.53E+01	-0.90
GOTERM_MF	GO:0008574	ATP-dependent microtubule motor activity plus-end-directed	4	*KIF4A*	-0.59	1.82E+01	-1.00

GOTERM_MF	GO:0008574	ATP-dependent microtubule motor activity plus-end-directed	4	*KIF11*	-0.94	1.82E+01	-1.00
GOTERM_MF	GO:0008574	ATP-dependent microtubule motor activity plus-end-directed	4	*KIF18A*	-0.69	1.82E+01	-1.00
GOTERM_MF	GO:0008574	ATP-dependent microtubule motor activity plus-end-directed	4	*KIF19*	0.64	1.82E+01	-1.00
GOTERM_MF	GO:0008301	DNA binding bending	4	*HMGB1*	-0.52	1.15E+01	-1.00
GOTERM_MF	GO:0008301	DNA binding bending	4	*HMGB2*	-0.59	1.15E+01	-1.00
GOTERM_MF	GO:0008301	DNA binding bending	4	*CRIP1*	0.57	1.15E+01	-1.00
GOTERM_MF	GO:0008301	DNA binding bending	4	*TOP2A*	-0.76	1.15E+01	-1.00
GOTERM_CC	GO:0045120	pronucleus	4	*HNF1A*	0.50	6.42E+00	-1.00
GOTERM_CC	GO:0045120	pronucleus	4	*WASF1*	-1.20	6.42E+00	-1.00
GOTERM_CC	GO:0045120	pronucleus	4	*CENPF*	-0.82	6.42E+00	-1.00
GOTERM_CC	GO:0045120	pronucleus	4	*AURKA*	-0.52	6.42E+00	-1.00
GOTERM_MF	GO:0042056	chemoattractant activity	4	*HMGB1*	-0.52	6.13E+01	-1.00
GOTERM_MF	GO:0042056	chemoattractant activity	4	*HMGB2*	-0.59	6.13E+01	-1.00
GOTERM_MF	GO:0042056	chemoattractant activity	4	*FGF7*	-0.87	6.13E+01	-1.00
GOTERM_MF	GO:0042056	chemoattractant activity	4	*M-SAA3.2*	1.80	6.13E+01	-1.00
GOTERM_BP	GO:0006974	cellular response to DNA damage stimulus	10	*SGK1*	0.54	5.25E+01	-1.26
GOTERM_BP	GO:0006974	cellular response to DNA damage stimulus	10	*MCM8*	-0.61	5.25E+01	-1.26
GOTERM_BP	GO:0006974	cellular response to DNA damage stimulus	10	*BTG2*	0.55	5.25E+01	-1.26
GOTERM_BP	GO:0006974	cellular response to DNA damage stimulus	10	*APITD1*	-0.76	5.25E+01	-1.26

GOTERM_BP	GO:0006974	cellular response to DNA damage stimulus	10	*WDR76*	-1.21	5.25E+01	-1.26
GOTERM_BP	GO:0006974	cellular response to DNA damage stimulus	10	*KIAA0101*	-0.78	5.25E+01	-1.26
GOTERM_BP	GO:0006974	cellular response to DNA damage stimulus	10	*MASTL*	-0.60	5.25E+01	-1.26
GOTERM_BP	GO:0006974	cellular response to DNA damage stimulus	10	*MCM10*	-0.76	5.25E+01	-1.26
GOTERM_BP	GO:0006974	cellular response to DNA damage stimulus	10	*TOP2A*	-0.76	5.25E+01	-1.26
GOTERM_BP	GO:0006974	cellular response to DNA damage stimulus	10	*ZNF385A*	0.63	5.25E+01	-1.26
GOTERM_MF	GO:0003777	microtubule motor activity	10	*KIFC2*	0.50	5.11E-01	-1.26
GOTERM_MF	GO:0003777	microtubule motor activity	10	*KIF2C*	-0.61	5.11E-01	-1.26
GOTERM_MF	GO:0003777	microtubule motor activity	10	*KIF22*	-0.55	5.11E-01	-1.26
GOTERM_MF	GO:0003777	microtubule motor activity	10	*KIF24*	-0.55	5.11E-01	-1.26
GOTERM_MF	GO:0003777	microtubule motor activity	10	*KIF15*	-0.95	5.11E-01	-1.26
GOTERM_MF	GO:0003777	microtubule motor activity	10	*KIF20B*	-0.65	5.11E-01	-1.26
GOTERM_MF	GO:0003777	microtubule motor activity	10	*CENPE*	-0.75	5.11E-01	-1.26
GOTERM_MF	GO:0003777	microtubule motor activity	10	*KIF19*	0.64	5.11E-01	-1.26
GOTERM_MF	GO:0003777	microtubule motor activity	10	*DNAH5*	-0.83	5.11E-01	-1.26
GOTERM_MF	GO:0003777	microtubule motor activity	10	*KIFC3*	0.77	5.11E-01	-1.26
GOTERM_MF	GO:0046982	protein heterodimerization activity	10	*FOS*	1.03	3.98E+01	-1.26
GOTERM_MF	GO:0046982	protein heterodimerization activity	10	*HIST1H2BB*	-0.64	3.98E+01	-1.26
GOTERM_MF	GO:0046982	protein heterodimerization activity	10	*HIST1H2BN*	-0.72	3.98E+01	-1.26
GOTERM_MF	GO:0046982	protein heterodimerization activity	10	*APITD1*	-0.76	3.98E+01	-1.26
GOTERM_MF	GO:0046982	protein heterodimerization activity	10	*BCL2A1*	-0.69	3.98E+01	-1.26

GOTERM_MF	GO:0046982	protein heterodimerization activity	10	*H2AFZ*	-0.60	3.98E+01	-1.26
GOTERM_MF	GO:0046982	protein heterodimerization activity	10	*NR4A1*	1.03	3.98E+01	-1.26
GOTERM_MF	GO:0046982	protein heterodimerization activity	10	*SLC51A*	0.78	3.98E+01	-1.26
GOTERM_MF	GO:0046982	protein heterodimerization activity	10	*SMC2*	-0.71	3.98E+01	-1.26
GOTERM_MF	GO:0046982	protein heterodimerization activity	10	*LOC504599*	-0.79	3.98E+01	-1.26
GOTERM_BP	GO:0050853	B cell receptor signaling pathway	5	*KLHL6*	-0.94	2.91E+01	-1.34
GOTERM_BP	GO:0050853	B cell receptor signaling pathway	5	*CD19*	-1.06	2.91E+01	-1.34
GOTERM_BP	GO:0050853	B cell receptor signaling pathway	5	*BLK*	-1.03	2.91E+01	-1.34
GOTERM_BP	GO:0050853	B cell receptor signaling pathway	5	*CD79B*	-3.11	2.91E+01	-1.34
GOTERM_BP	GO:0050853	B cell receptor signaling pathway	5	*NFAM1*	0.59	2.91E+01	-1.34
GOTERM_CC	GO:0005719	nuclear euchromatin	5	*HIST1H1D*	-0.55	1.70E+01	-1.34
GOTERM_CC	GO:0005719	nuclear euchromatin	5	*HIST1H1A*	-1.47	1.70E+01	-1.34
GOTERM_CC	GO:0005719	nuclear euchromatin	5	*JUN*	0.57	1.70E+01	-1.34
GOTERM_CC	GO:0005719	nuclear euchromatin	5	*H2AFZ*	-0.60	1.70E+01	-1.34
GOTERM_CC	GO:0005719	nuclear euchromatin	5	*CBX3*	-0.59	1.70E+01	-1.34
GOTERM_CC	GO:0072686	mitotic spindle	5	*CDK1*	-0.54	5.02E+01	-1.34
GOTERM_CC	GO:0072686	mitotic spindle	5	*KIF22*	-0.55	5.02E+01	-1.34
GOTERM_CC	GO:0072686	mitotic spindle	5	*SPAG5*	-0.73	5.02E+01	-1.34
GOTERM_CC	GO:0072686	mitotic spindle	5	*CAPG*	2.06	5.02E+01	-1.34
GOTERM_CC	GO:0072686	mitotic spindle	5	*AURKA*	-0.52	5.02E+01	-1.34
GOTERM_MF	GO:0003682	chromatin binding	20	*EXO1*	-0.70	4.64E+01	-1.34
GOTERM_MF	GO:0003682	chromatin binding	20	*CDK1*	-0.54	4.64E+01	-1.34
GOTERM_MF	GO:0003682	chromatin binding	20	*HIST1H1E*	-0.54	4.64E+01	-1.34
GOTERM_MF	GO:0003682	chromatin binding	20	*CAMTA2*	0.56	4.64E+01	-1.34
GOTERM_MF	GO:0003682	chromatin binding	20	*TICRR*	-0.54	4.64E+01	-1.34

GOTERM_MF	GO:0003682	chromatin binding	20	*POLE*	-0.66	4.64E+01	-1.34
GOTERM_MF	GO:0003682	chromatin binding	20	*KIAA0101*	-0.78	4.64E+01	-1.34
GOTERM_MF	GO:0003682	chromatin binding	20	*TLE4*	0.64	4.64E+01	-1.34
GOTERM_MF	GO:0003682	chromatin binding	20	*MCM5*	-0.51	4.64E+01	-1.34
GOTERM_MF	GO:0003682	chromatin binding	20	*CITED2*	0.55	4.64E+01	-1.34
GOTERM_MF	GO:0003682	chromatin binding	20	*FOS*	1.03	4.64E+01	-1.34
GOTERM_MF	GO:0003682	chromatin binding	20	*PCGF2*	0.57	4.64E+01	-1.34
GOTERM_MF	GO:0003682	chromatin binding	20	*APITD1*	-0.76	4.64E+01	-1.34
GOTERM_MF	GO:0003682	chromatin binding	20	*GATA5*	0.52	4.64E+01	-1.34
GOTERM_MF	GO:0003682	chromatin binding	20	*JUN*	0.57	4.64E+01	-1.34
GOTERM_MF	GO:0003682	chromatin binding	20	*PCNA*	-0.78	4.64E+01	-1.34
GOTERM_MF	GO:0003682	chromatin binding	20	*CKS2*	-0.50	4.64E+01	-1.34
GOTERM_MF	GO:0003682	chromatin binding	20	*ORC1*	-0.89	4.64E+01	-1.34
GOTERM_MF	GO:0003682	chromatin binding	20	*TOP2A*	-0.76	4.64E+01	-1.34
GOTERM_MF	GO:0003682	chromatin binding	20	*HELLS*	-0.80	4.64E+01	-1.34
GOTERM_MF	GO:0044378	non-sequence-specific DNA binding bending	2	*HMGB1*	-0.52	7.01E+01	-1.41
GOTERM_MF	GO:0044378	non-sequence-specific DNA binding bending	2	*HMGB2*	-0.59	7.01E+01	-1.41
GOTERM_BP	GO:0051383	kinetochore organization	2	*SMC2*	-0.71	7.61E+01	-1.41
GOTERM_BP	GO:0051383	kinetochore organization	2	*SMC4*	-0.58	7.61E+01	-1.41
GOTERM_BP	GO:0009186	deoxyribonucleoside diphosphate metabolic process	2	*RRM2*	-0.64	7.61E+01	-1.41
GOTERM_BP	GO:0009186	deoxyribonucleoside diphosphate metabolic process	2	*RRM2B*	-0.64	7.61E+01	-1.41
GOTERM_CC	GO:0005814	centriole	8	*PLK4*	-0.55	5.36E+01	-1.41

GOTERM_CC	GO:0005814	centriole	8	*KIF24*	-0.55		5.36E+01	-1.41
GOTERM_CC	GO:0005814	centriole	8	*CETN4*	-0.55		5.36E+01	-1.41
GOTERM_CC	GO:0005814	centriole	8	*CAPG*	2.06		5.36E+01	-1.41
GOTERM_CC	GO:0005814	centriole	8	*AURKA*	-0.52		5.36E+01	-1.41
GOTERM_CC	GO:0005814	centriole	8	*HSPA1A*	0.77		5.36E+01	-1.41
GOTERM_CC	GO:0005814	centriole	8	*PLA2G3*	-1.76		5.36E+01	-1.41
GOTERM_CC	GO:0005814	centriole	8	*TOP2A*	-0.76		5.36E+01	-1.41
GOTERM_CC	GO:0031436	BRCA1-BARD1 complex	2	*BRCA1*	-0.56		6.84E+01	-1.41
GOTERM_CC	GO:0031436	BRCA1-BARD1 complex	2	*BARD1*	-0.68		6.84E+01	-1.41
GOTERM_MF	GO:0032405	MutLalpha complex binding	2	*MSH2*	-0.81		7.01E+01	-1.41
GOTERM_MF	GO:0032405	MutLalpha complex binding	2	*PCNA*	-0.78		7.01E+01	-1.41
KEGG_PATHWAY	bta04662	B cell receptor signaling pathway	8	*FOS*	1.03		3.38E+01	-1.41
KEGG_PATHWAY	bta04662	B cell receptor signaling pathway	8	*CR2*	-3.53		3.38E+01	-1.41
KEGG_PATHWAY	bta04662	B cell receptor signaling pathway	8	*CD19*	-1.06		3.38E+01	-1.41
KEGG_PATHWAY	bta04662	B cell receptor signaling pathway	8	*RASGRP3*	-0.71		3.38E+01	-1.41
KEGG_PATHWAY	bta04662	B cell receptor signaling pathway	8	*JUN*	0.57		3.38E+01	-1.41
KEGG_PATHWAY	bta04662	B cell receptor signaling pathway	8	*CD22*	-1.72		3.38E+01	-1.41
KEGG_PATHWAY	bta04662	B cell receptor signaling pathway	8	*CD79B*	-3.11		3.38E+01	-1.41
KEGG_PATHWAY	bta04662	B cell receptor signaling pathway	8	*CD72*	-0.68		3.38E+01	-1.41
GOTERM_BP	GO:0042127	regulation of cell proliferation	11	*SGK1*	0.54	0.074	7.32E+01	-1.51
GOTERM_BP	GO:0042127	regulation of cell proliferation	11	*CDCA7*	-0.59	0.074	7.32E+01	-1.51
GOTERM_BP	GO:0042127	regulation of cell proliferation	11	*CXCL13*	-3.12	0.074	7.32E+01	-1.51
GOTERM_BP	GO:0042127	regulation of cell proliferation	11	*BLK*	-1.03	0.074	7.32E+01	-1.51
GOTERM_BP	GO:0042127	regulation of cell proliferation	11	*TNC*	-0.53	0.074	7.32E+01	-1.51
GOTERM_BP	GO:0042127	regulation of cell proliferation	11	*JUN*	0.57	0.074	7.32E+01	-1.51

GOTERM_BP	GO:0042127	regulation of cell proliferation	11	*TNFRSF18*	-0.63	0.074	7.32E+01	-1.51
GOTERM_BP	GO:0042127	regulation of cell proliferation	11	*BRCA2*	-0.67	0.074	7.32E+01	-1.51
GOTERM_BP	GO:0042127	regulation of cell proliferation	11	*PLCD1*	0.78	0.074	7.32E+01	-1.51
GOTERM_BP	GO:0042127	regulation of cell proliferation	11	*CD40*	-0.71	0.074	7.32E+01	-1.51
GOTERM_BP	GO:0042127	regulation of cell proliferation	11	*RPA3*	-0.82	0.074	7.32E+01	-1.51
GOTERM_CC	GO:0015629	actin cytoskeleton	11	*NDC1*	-0.79	0.048	4.89E+01	-1.51
GOTERM_CC	GO:0015629	actin cytoskeleton	11	*SLC16A3*	0.65	0.048	4.89E+01	-1.51
GOTERM_CC	GO:0015629	actin cytoskeleton	11	*TARS*	-1.19	0.048	4.89E+01	-1.51
GOTERM_CC	GO:0015629	actin cytoskeleton	11	*NCAPG*	-0.74	0.048	4.89E+01	-1.51
GOTERM_CC	GO:0015629	actin cytoskeleton	11	*CENPQ*	-0.60	0.048	4.89E+01	-1.51
GOTERM_CC	GO:0015629	actin cytoskeleton	11	*CFL2*	-0.65	0.048	4.89E+01	-1.51
GOTERM_CC	GO:0015629	actin cytoskeleton	11	*KNTC1*	-0.74	0.048	4.89E+01	-1.51
GOTERM_CC	GO:0015629	actin cytoskeleton	11	*RAI14*	-0.50	0.048	4.89E+01	-1.51
GOTERM_CC	GO:0015629	actin cytoskeleton	11	*RARA*	0.54	0.048	4.89E+01	-1.51
GOTERM_CC	GO:0015629	actin cytoskeleton	11	*TOPBP1*	-0.54	0.048	4.89E+01	-1.51
GOTERM_CC	GO:0015629	actin cytoskeleton	11	*ESPN*	0.71	0.048	4.89E+01	-1.51
GOTERM_MF	GO:0016887	ATPase activity	11	*KIFC2*	0.50	0.009	1.21E+01	-1.51
GOTERM_MF	GO:0016887	ATPase activity	11	*KIF2C*	-0.61	0.009	1.21E+01	-1.51
GOTERM_MF	GO:0016887	ATPase activity	11	*KIF22*	-0.55	0.009	1.21E+01	-1.51
GOTERM_MF	GO:0016887	ATPase activity	11	*KIF24*	-0.55	0.009	1.21E+01	-1.51
GOTERM_MF	GO:0016887	ATPase activity	11	*MSH2*	-0.81	0.009	1.21E+01	-1.51
GOTERM_MF	GO:0016887	ATPase activity	11	*KIF15*	-0.95	0.009	1.21E+01	-1.51
GOTERM_MF	GO:0016887	ATPase activity	11	*KIF20B*	-0.65	0.009	1.21E+01	-1.51
GOTERM_MF	GO:0016887	ATPase activity	11	*CENPE*	-0.75	0.009	1.21E+01	-1.51
GOTERM_MF	GO:0016887	ATPase activity	11	*KIF19*	0.64	0.009	1.21E+01	-1.51

GOTERM_MF	GO:0016887	ATPase activity	11	*DNAH5*	-0.83	0.009	1.21E+01	-1.51
GOTERM_MF	GO:0016887	ATPase activity	11	*KIFC3*	0.77	0.009	1.21E+01	-1.51
GOTERM_MF	GO:0003690	double-stranded DNA binding	6	*FOS*	1.03	0.039	4.43E+01	-1.63
GOTERM_MF	GO:0003690	double-stranded DNA binding	6	*HMGB1*	-0.52	0.039	4.43E+01	-1.63
GOTERM_MF	GO:0003690	double-stranded DNA binding	6	*HMGB2*	-0.59	0.039	4.43E+01	-1.63
GOTERM_MF	GO:0003690	double-stranded DNA binding	6	*APITD1*	-0.76	0.039	4.43E+01	-1.63
GOTERM_MF	GO:0003690	double-stranded DNA binding	6	*NEIL3*	-0.75	0.039	4.43E+01	-1.63
GOTERM_MF	GO:0003690	double-stranded DNA binding	6	*MND1*	-0.62	0.039	4.43E+01	-1.63
GOTERM_BP	GO:0007018	microtubule-based movement	12	*KIFC2*	0.50	0.000	1.81E-02	-1.73
GOTERM_BP	GO:0007018	microtubule-based movement	12	*KIF2C*	-0.61	0.000	1.81E-02	-1.73
GOTERM_BP	GO:0007018	microtubule-based movement	12	*KIF22*	-0.55	0.000	1.81E-02	-1.73
GOTERM_BP	GO:0007018	microtubule-based movement	12	*KIF4A*	-0.59	0.000	1.81E-02	-1.73
GOTERM_BP	GO:0007018	microtubule-based movement	12	*KIF11*	-0.94	0.000	1.81E-02	-1.73
GOTERM_BP	GO:0007018	microtubule-based movement	12	*KIF24*	-0.55	0.000	1.81E-02	-1.73
GOTERM_BP	GO:0007018	microtubule-based movement	12	*KIF15*	-0.95	0.000	1.81E-02	-1.73
GOTERM_BP	GO:0007018	microtubule-based movement	12	*KIF18A*	-0.69	0.000	1.81E-02	-1.73
GOTERM_BP	GO:0007018	microtubule-based movement	12	*KIF20B*	-0.65	0.000	1.81E-02	-1.73
GOTERM_BP	GO:0007018	microtubule-based movement	12	*CENPE*	-0.75	0.000	1.81E-02	-1.73
GOTERM_BP	GO:0007018	microtubule-based movement	12	*KIF19*	0.64	0.000	1.81E-02	-1.73
GOTERM_BP	GO:0007018	microtubule-based movement	12	*KIFC3*	0.77	0.000	1.81E-02	-1.73
GOTERM_BP	GO:0031052	chromosome breakage	3	*CD19*	-1.06	0.015	2.32E+01	-1.73
GOTERM_BP	GO:0031052	chromosome breakage	3	*BRCA2*	-0.67	0.015	2.32E+01	-1.73
GOTERM_BP	GO:0031052	chromosome breakage	3	*BRCA1*	-0.56	0.015	2.32E+01	-1.73
GOTERM_BP	GO:0033314	mitotic DNA replication checkpoint	3	*CLSPN*	-0.73	0.022	3.20E+01	-1.73
GOTERM_BP	GO:0033314	mitotic DNA replication checkpoint	3	*TICRR*	-0.54	0.022	3.20E+01	-1.73

GOTERM_BP	GO:0033314	mitotic DNA replication checkpoint	3	*TOPBP1*	-0.54	0.022	3.20E+01	-1.73
GOTERM_BP	GO:0051310	metaphase plate congression	3	*FAM83D*	-0.51	0.030	4.10E+01	-1.73
GOTERM_BP	GO:0051310	metaphase plate congression	3	*KIF22*	-0.55	0.030	4.10E+01	-1.73
GOTERM_BP	GO:0051310	metaphase plate congression	3	*CENPF*	-0.82	0.030	4.10E+01	-1.73
GOTERM_BP	GO:0031110	regulation of microtubule polymerization or depolymerization	3	*SKA2*	-0.67	0.030	4.10E+01	-1.73
GOTERM_BP	GO:0031110	regulation of microtubule polymerization or depolymerization	3	*STMN1*	-1.40	0.030	4.10E+01	-1.73
GOTERM_BP	GO:0031110	regulation of microtubule polymerization or depolymerization	3	*SKA1*	-0.81	0.030	4.10E+01	-1.73
GOTERM_BP	GO:0010389	regulation of G2/M transition of mitotic cell cycle	3	*CENPF*	-0.82	0.030	4.10E+01	-1.73
GOTERM_BP	GO:0010389	regulation of G2/M transition of mitotic cell cycle	3	*CCNA2*	-0.67	0.030	4.10E+01	-1.73
GOTERM_BP	GO:0010389	regulation of G2/M transition of mitotic cell cycle	3	*PKIA*	-1.54	0.030	4.10E+01	-1.73
GOTERM_BP	GO:0006265	DNA topological change	3	*HMGB1*	-0.52	0.030	4.10E+01	-1.73
GOTERM_BP	GO:0006265	DNA topological change	3	*HMGB2*	-0.59	0.030	4.10E+01	-1.73
GOTERM_BP	GO:0006265	DNA topological change	3	*TOP2A*	-0.76	0.030	4.10E+01	-1.73
GOTERM_BP	GO:0030071	regulation of mitotic metaphase/anaphase transition	3	*CDC6*	-0.76	0.039	4.98E+01	-1.73
GOTERM_BP	GO:0030071	regulation of mitotic metaphase/anaphase transition	3	*CENPE*	-0.75	0.039	4.98E+01	-1.73
GOTERM_BP	GO:0030071	regulation of mitotic metaphase/anaphase transition	3	*UBE2C*	-0.69	0.039	4.98E+01	-1.73
GOTERM_BP	GO:0019985	translesion synthesis	3	*DTL*	-0.53	0.049	5.80E+01	-1.73

GOTERM_BP	GO:0019985	translesion synthesis	3	*KIAA0101*	-0.78	0.049	5.80E+01	-1.73
GOTERM_BP	GO:0019985	translesion synthesis	3	*PCNA*	-0.78	0.049	5.80E+01	-1.73
GOTERM_BP	GO:0016446	somatic hypermutation of immunoglobulin genes	3	*EXO1*	-0.70	0.060	6.54E+01	-1.73
GOTERM_BP	GO:0016446	somatic hypermutation of immunoglobulin genes	3	*MSH2*	-0.81	0.060	6.54E+01	-1.73
GOTERM_BP	GO:0016446	somatic hypermutation of immunoglobulin genes	3	*UNG*	-0.57	0.060	6.54E+01	-1.73
GOTERM_BP	GO:0006301	postreplication repair	3	*MSH2*	-0.81	0.071	7.19E+01	-1.73
GOTERM_BP	GO:0006301	postreplication repair	3	*RAD18*	-0.58	0.071	7.19E+01	-1.73
GOTERM_BP	GO:0006301	postreplication repair	3	*BRCA1*	-0.56	0.071	7.19E+01	-1.73
GOTERM_BP	GO:0021670	lateral ventricle development	3	*PAX5*	-4.82	0.071	7.19E+01	-1.73
GOTERM_BP	GO:0021670	lateral ventricle development	3	*DNAH5*	-0.83	0.071	7.19E+01	-1.73
GOTERM_BP	GO:0021670	lateral ventricle development	3	*DPCD*	-0.60	0.071	7.19E+01	-1.73
GOTERM_BP	GO:0008340	determination of adult lifespan	3	*MSH2*	-0.81	0.083	7.75E+01	-1.73
GOTERM_BP	GO:0008340	determination of adult lifespan	3	*LRRK2*	-0.58	0.083	7.75E+01	-1.73
GOTERM_BP	GO:0008340	determination of adult lifespan	3	*RAD54L*	-0.54	0.083	7.75E+01	-1.73
GOTERM_CC	GO:0005871	kinesin complex	12	*KIFC2*	0.50	0.000	2.57E-03	-1.73
GOTERM_CC	GO:0005871	kinesin complex	12	*KIF2C*	-0.61	0.000	2.57E-03	-1.73
GOTERM_CC	GO:0005871	kinesin complex	12	*KIF22*	-0.55	0.000	2.57E-03	-1.73
GOTERM_CC	GO:0005871	kinesin complex	12	*KIF4A*	-0.59	0.000	2.57E-03	-1.73
GOTERM_CC	GO:0005871	kinesin complex	12	*KIF11*	-0.94	0.000	2.57E-03	-1.73
GOTERM_CC	GO:0005871	kinesin complex	12	*KIF24*	-0.55	0.000	2.57E-03	-1.73
GOTERM_CC	GO:0005871	kinesin complex	12	*KIF15*	-0.95	0.000	2.57E-03	-1.73
GOTERM_CC	GO:0005871	kinesin complex	12	*KIF18A*	-0.69	0.000	2.57E-03	-1.73

GOTERM_CC	GO:0005871	kinesin complex	12	*KIF20B*	-0.65	0.000	2.57E-03	-1.73
GOTERM_CC	GO:0005871	kinesin complex	12	*CENPE*	-0.75	0.000	2.57E-03	-1.73
GOTERM_CC	GO:0005871	kinesin complex	12	*KIF19*	0.64	0.000	2.57E-03	-1.73
GOTERM_CC	GO:0005871	kinesin complex	12	*KIFC3*	0.77	0.000	2.57E-03	-1.73
GOTERM_CC	GO:0000778	condensed nuclear chromosome kinetochore	3	*BUB1B*	-0.69	0.010	1.24E+01	-1.73
GOTERM_CC	GO:0000778	condensed nuclear chromosome kinetochore	3	*NDC80*	-0.79	0.010	1.24E+01	-1.73
GOTERM_CC	GO:0000778	condensed nuclear chromosome kinetochore	3	*MIS18BP1*	-0.67	0.010	1.24E+01	-1.73
GOTERM_CC	GO:0000796	condensin complex	3	*NCAPG*	-0.74	0.010	1.24E+01	-1.73
GOTERM_CC	GO:0000796	condensin complex	3	*SMC2*	-0.71	0.010	1.24E+01	-1.73
GOTERM_CC	GO:0000796	condensin complex	3	*SMC4*	-0.58	0.010	1.24E+01	-1.73
GOTERM_CC	GO:0000942	condensed nuclear chromosome outer kinetochore	3	*CCNB1*	-0.73	0.010	1.24E+01	-1.73
GOTERM_CC	GO:0000942	condensed nuclear chromosome outer kinetochore	3	*BUB1*	-0.71	0.010	1.24E+01	-1.73
GOTERM_CC	GO:0000942	condensed nuclear chromosome outer kinetochore	3	*NDC80*	-0.79	0.010	1.24E+01	-1.73
GOTERM_CC	GO:0032133	chromosome passenger complex	3	*CDCA8*	-0.67	0.016	1.93E+01	-1.73
GOTERM_CC	GO:0032133	chromosome passenger complex	3	*AURKA*	-0.52	0.016	1.93E+01	-1.73
GOTERM_CC	GO:0032133	chromosome passenger complex	3	*AURKB*	-0.69	0.016	1.93E+01	-1.73
GOTERM_CC	GO:0070531	BRCA1-A complex	3	*FAM175A*	-0.68		3.50E+01	-1.73
GOTERM_CC	GO:0070531	BRCA1-A complex	3	*BRCA1*	-0.56		3.50E+01	-1.73
GOTERM_CC	GO:0070531	BRCA1-A complex	3	*BARD1*	-0.68		3.50E+01	-1.73
GOTERM_CC	GO:0070938	contractile ring	3	*PRC1*	-0.54		3.50E+01	-1.73

GOTERM_CC	GO:0070938	contractile ring	3	*AURKA*	-0.52	3.50E+01	-1.73
GOTERM_CC	GO:0070938	contractile ring	3	*AURKB*	-0.69	3.50E+01	-1.73
GOTERM_CC	GO:0031616	spindle pole centrosome	3	*DLGAP5*	-0.90	5.06E+01	-1.73
GOTERM_CC	GO:0031616	spindle pole centrosome	3	*AURKA*	-0.52	5.06E+01	-1.73
GOTERM_CC	GO:0031616	spindle pole centrosome	3	*AURKB*	-0.69	5.06E+01	-1.73
GOTERM_CC	GO:0010369	chromocenter	3	*CDCA8*	-0.67	5.78E+01	-1.73
GOTERM_CC	GO:0010369	chromocenter	3	*OIP5*	-0.69	5.78E+01	-1.73
GOTERM_CC	GO:0010369	chromocenter	3	*AURKB*	-0.69	5.78E+01	-1.73
GOTERM_CC	GO:0000228	nuclear chromosome	3	*MSH2*	-0.81	7.56E+01	-1.73
GOTERM_CC	GO:0000228	nuclear chromosome	3	*TOP2A*	-0.76	7.56E+01	-1.73
GOTERM_CC	GO:0000228	nuclear chromosome	3	*SMC2*	-0.71	7.56E+01	-1.73
GOTERM_MF	GO:0003678	DNA helicase activity	3	*MCM3*	-0.67	6.53E+01	-1.73
GOTERM_MF	GO:0003678	DNA helicase activity	3	*MCM5*	-0.51	6.53E+01	-1.73
GOTERM_MF	GO:0003678	DNA helicase activity	3	*MCM6*	-0.70	6.53E+01	-1.73
GOTERM_MF	GO:0043142	single-stranded DNA-dependent ATPase activity	3	*RFC4*	-0.56	6.53E+01	-1.73
GOTERM_MF	GO:0043142	single-stranded DNA-dependent ATPase activity	3	*RFC2*	-0.59	6.53E+01	-1.73
GOTERM_MF	GO:0043142	single-stranded DNA-dependent ATPase activity	3	*RAD18*	-0.58	6.53E+01	-1.73
GOTERM_BP	GO:0007019	microtubule depolymerization	4	*KIF2C*	-0.61	1.38E+01	-2.00
GOTERM_BP	GO:0007019	microtubule depolymerization	4	*KIF24*	-0.55	1.38E+01	-2.00
GOTERM_BP	GO:0007019	microtubule depolymerization	4	*KIF18A*	-0.69	1.38E+01	-2.00
GOTERM_BP	GO:0007019	microtubule depolymerization	4	*STMN1*	-1.40	1.38E+01	-2.00
GOTERM_BP	GO:0007076	mitotic chromosome condensation	4	*NCAPG*	-0.74	1.75E+01	-2.00

GOTERM_BP	GO:0007076	mitotic chromosome condensation	4	*NUSAP1*	-0.88	1.75E+01	-2.00
GOTERM_BP	GO:0007076	mitotic chromosome condensation	4	*SMC2*	-0.71	1.75E+01	-2.00
GOTERM_BP	GO:0007076	mitotic chromosome condensation	4	*SMC4*	-0.58	1.75E+01	-2.00
GOTERM_BP	GO:0032508	DNA duplex unwinding	4	*GINS2*	-0.60	5.03E+01	-2.00
GOTERM_BP	GO:0032508	DNA duplex unwinding	4	*MCM3*	-0.67	5.03E+01	-2.00
GOTERM_BP	GO:0032508	DNA duplex unwinding	4	*MCM5*	-0.51	5.03E+01	-2.00
GOTERM_BP	GO:0032508	DNA duplex unwinding	4	*MCM6*	-0.70	5.03E+01	-2.00
GOTERM_BP	GO:0031572	G2 DNA damage checkpoint	4	*CLSPN*	-0.73	5.03E+01	-2.00
GOTERM_BP	GO:0031572	G2 DNA damage checkpoint	4	*FAM175A*	-0.68	5.03E+01	-2.00
GOTERM_BP	GO:0031572	G2 DNA damage checkpoint	4	*DTL*	-0.53	5.03E+01	-2.00
GOTERM_BP	GO:0031572	G2 DNA damage checkpoint	4	*BRCA1*	-0.56	5.03E+01	-2.00
GOTERM_BP	GO:0032147	activation of protein kinase activity	4	*CLSPN*	-0.73	6.42E+01	-2.00
GOTERM_BP	GO:0032147	activation of protein kinase activity	4	*HMGB1*	-0.52	6.42E+01	-2.00
GOTERM_BP	GO:0032147	activation of protein kinase activity	4	*TPX2*	-0.71	6.42E+01	-2.00
GOTERM_BP	GO:0032147	activation of protein kinase activity	4	*ECT2*	-0.53	6.42E+01	-2.00
GOTERM_BP	GO:0045739	positive regulation of DNA repair	4	*FAM175A*	-0.68	6.83E+01	-2.00
GOTERM_BP	GO:0045739	positive regulation of DNA repair	4	*PCNA*	-0.78	6.83E+01	-2.00
GOTERM_BP	GO:0045739	positive regulation of DNA repair	4	*APEX1*	-0.50	6.83E+01	-2.00
GOTERM_BP	GO:0045739	positive regulation of DNA repair	4	*BRCA1*	-0.56	6.83E+01	-2.00
GOTERM_BP	GO:0000281	mitotic cytokinesis	4	*NUSAP1*	-0.88	7.22E+01	-2.00
GOTERM_BP	GO:0000281	mitotic cytokinesis	4	*ANLN*	-0.53	7.22E+01	-2.00
GOTERM_BP	GO:0000281	mitotic cytokinesis	4	*CEP55*	-0.65	7.22E+01	-2.00
GOTERM_BP	GO:0000281	mitotic cytokinesis	4	*STMN1*	-1.40	7.22E+01	-2.00
GOTERM_CC	GO:0031262	Ndc80 complex	4	*SPC24*	-0.56	3.68E-01	-2.00
GOTERM_CC	GO:0031262	Ndc80 complex	4	*SPC25*	-0.62	3.68E-01	-2.00

GOTERM_CC	GO:0031262	Ndc80 complex	4	*NUF2*	-0.76	3.68E-01	-2.00
GOTERM_CC	GO:0031262	Ndc80 complex	4	*NDC80*	-0.79	3.68E-01	-2.00
GOTERM_CC	GO:0000940	condensed chromosome outer kinetochore	4	*BUB1B*	-0.69	1.72E+00	-2.00
GOTERM_CC	GO:0000940	condensed chromosome outer kinetochore	4	*SKA2*	-0.67	1.72E+00	-2.00
GOTERM_CC	GO:0000940	condensed chromosome outer kinetochore	4	*SPDL1*	-0.78	1.72E+00	-2.00
GOTERM_CC	GO:0000940	condensed chromosome outer kinetochore	4	*SKA1*	-0.81	1.72E+00	-2.00
GOTERM_CC	GO:0042555	MCM complex	4	*MMS22L*	-0.54	6.42E+00	-2.00
GOTERM_CC	GO:0042555	MCM complex	4	*MCM3*	-0.67	6.42E+00	-2.00
GOTERM_CC	GO:0042555	MCM complex	4	*MCM5*	-0.51	6.42E+00	-2.00
GOTERM_CC	GO:0042555	MCM complex	4	*MCM6*	-0.70	6.42E+00	-2.00
KEGG_PATHWAY	bta04115	p53 signaling pathway	8	*CCNE2*	-0.74	3.57E+01	-2.12
KEGG_PATHWAY	bta04115	p53 signaling pathway	8	*CCNB1*	-0.73	3.57E+01	-2.12
KEGG_PATHWAY	bta04115	p53 signaling pathway	8	*CDK1*	-0.54	3.57E+01	-2.12
KEGG_PATHWAY	bta04115	p53 signaling pathway	8	*CCNE1*	-0.57	3.57E+01	-2.12
KEGG_PATHWAY	bta04115	p53 signaling pathway	8	*CCNB2*	-0.73	3.57E+01	-2.12
KEGG_PATHWAY	bta04115	p53 signaling pathway	8	*RRM2*	-0.64	3.57E+01	-2.12
KEGG_PATHWAY	bta04115	p53 signaling pathway	8	*RRM2B*	-0.64	3.57E+01	-2.12
KEGG_PATHWAY	bta04115	p53 signaling pathway	8	*SESN2*	0.65	3.57E+01	-2.12
KEGG_PATHWAY	bta04914	Progesterone-mediated oocyte maturation	8	*CCNB1*	-0.73	6.91E+01	-2.12
KEGG_PATHWAY	bta04914	Progesterone-mediated oocyte maturation	8	*CDK1*	-0.54	6.91E+01	-2.12

KEGG_PATHWAY	bta04914	Progesterone-mediated oocyte maturation	8	*MAD2L1*	-0.66	6.91E+01	-2.12
KEGG_PATHWAY	bta04914	Progesterone-mediated oocyte maturation	8	*CCNB2*	-0.73	6.91E+01	-2.12
KEGG_PATHWAY	bta04914	Progesterone-mediated oocyte maturation	8	*BUB1*	-0.71	6.91E+01	-2.12
KEGG_PATHWAY	bta04914	Progesterone-mediated oocyte maturation	8	*PKMYT1*	-0.76	6.91E+01	-2.12
KEGG_PATHWAY	bta04914	Progesterone-mediated oocyte maturation	8	*MAPK11*	1.10	6.91E+01	-2.12
KEGG_PATHWAY	bta04914	Progesterone-mediated oocyte maturation	8	*CCNA2*	-0.67	6.91E+01	-2.12
GOTERM_CC	GO:0000775	chromosome centromeric region	5	*CENPN*	-0.62	4.04E+01	-2.24
GOTERM_CC	GO:0000775	chromosome centromeric region	5	*CDCA8*	-0.67	4.04E+01	-2.24
GOTERM_CC	GO:0000775	chromosome centromeric region	5	*OIP5*	-0.69	4.04E+01	-2.24
GOTERM_CC	GO:0000775	chromosome centromeric region	5	*CENPP*	-0.83	4.04E+01	-2.24
GOTERM_CC	GO:0000775	chromosome centromeric region	5	*AURKB*	-0.69	4.04E+01	-2.24
GOTERM_BP	GO:0034501	protein localization to kinetochore	5	*CDK1*	-0.54	2.84E-01	-2.24
GOTERM_BP	GO:0034501	protein localization to kinetochore	5	*MTBP*	-0.64	2.84E-01	-2.24
GOTERM_BP	GO:0034501	protein localization to kinetochore	5	*BUB1B*	-0.69	2.84E-01	-2.24
GOTERM_BP	GO:0034501	protein localization to kinetochore	5	*SPDL1*	-0.78	2.84E-01	-2.24
GOTERM_BP	GO:0034501	protein localization to kinetochore	5	*AURKB*	-0.69	2.84E-01	-2.24
GOTERM_BP	GO:0006284	base-excision repair	5	*HMGB1*	-0.52	4.28E+01	-2.24
GOTERM_BP	GO:0006284	base-excision repair	5	*NEIL3*	-0.75	4.28E+01	-2.24
GOTERM_BP	GO:0006284	base-excision repair	5	*APEX1*	-0.50	4.28E+01	-2.24
GOTERM_BP	GO:0006284	base-excision repair	5	*FEN1*	-0.55	4.28E+01	-2.24
GOTERM_BP	GO:0006284	base-excision repair	5	*RPA3*	-0.82	4.28E+01	-2.24

GOTERM_BP	GO:0010212	response to ionizing radiation	5	*FAM175A*	-0.68	6.66E+01	-2.24
GOTERM_BP	GO:0010212	response to ionizing radiation	5	*TICRR*	-0.54	6.66E+01	-2.24
GOTERM_BP	GO:0010212	response to ionizing radiation	5	*TOPBP1*	-0.54	6.66E+01	-2.24
GOTERM_BP	GO:0010212	response to ionizing radiation	5	*RAD54L*	-0.54	6.66E+01	-2.24
GOTERM_BP	GO:0010212	response to ionizing radiation	5	*BRCA1*	-0.56	6.66E+01	-2.24
GOTERM_BP	GO:0000082	G1/S transition of mitotic cell cycle	5	*CCNE2*	-0.74	7.52E+01	-2.24
GOTERM_BP	GO:0000082	G1/S transition of mitotic cell cycle	5	*CCNE1*	-0.57	7.52E+01	-2.24
GOTERM_BP	GO:0000082	G1/S transition of mitotic cell cycle	5	*CDKN2C*	-0.65	7.52E+01	-2.24
GOTERM_BP	GO:0000082	G1/S transition of mitotic cell cycle	5	*POLE*	-0.66	7.52E+01	-2.24
GOTERM_BP	GO:0000082	G1/S transition of mitotic cell cycle	5	*CDKN3*	-0.60	7.52E+01	-2.24
GOTERM_CC	GO:0000793	condensed chromosome	5	*HMGB1*	-0.52	4.66E+00	-2.24
GOTERM_CC	GO:0000793	condensed chromosome	5	*HMGB2*	-0.59	4.66E+00	-2.24
GOTERM_CC	GO:0000793	condensed chromosome	5	*NCAPG*	-0.74	4.66E+00	-2.24
GOTERM_CC	GO:0000793	condensed chromosome	5	*FANCD2*	-0.91	4.66E+00	-2.24
GOTERM_CC	GO:0000793	condensed chromosome	5	*TOP2A*	-0.76	4.66E+00	-2.24
GOTERM_MF	GO:0008307	structural constituent of muscle	5	*MYH11*	-0.87	4.54E+00	-2.24
GOTERM_MF	GO:0008307	structural constituent of muscle	5	*SYNM*	-1.02	4.54E+00	-2.24
GOTERM_MF	GO:0008307	structural constituent of muscle	5	*NEXN*	-0.82	4.54E+00	-2.24
GOTERM_MF	GO:0008307	structural constituent of muscle	5	*TPM2*	-1.04	4.54E+00	-2.24
GOTERM_MF	GO:0008307	structural constituent of muscle	5	*TPM1*	-0.86	4.54E+00	-2.24
GOTERM_BP	GO:0018105	peptidyl-serine phosphorylation	9	*CLSPN*	-0.73	7.85E+01	-2.33
GOTERM_BP	GO:0018105	peptidyl-serine phosphorylation	9	*CDK1*	-0.54	7.85E+01	-2.33
GOTERM_BP	GO:0018105	peptidyl-serine phosphorylation	9	*SGK1*	0.54	7.85E+01	-2.33
GOTERM_BP	GO:0018105	peptidyl-serine phosphorylation	9	*VRK1*	-0.58	7.85E+01	-2.33
GOTERM_BP	GO:0018105	peptidyl-serine phosphorylation	9	*MASTL*	-0.60	7.85E+01	-2.33

GOTERM_BP	GO:0018105	peptidyl-serine phosphorylation	9	*LRRK2*	-0.58	7.85E+01	-2.33
GOTERM_BP	GO:0018105	peptidyl-serine phosphorylation	9	*PRKD3*	-0.61	7.85E+01	-2.33
GOTERM_BP	GO:0018105	peptidyl-serine phosphorylation	9	*DCLK1*	-0.94	7.85E+01	-2.33
GOTERM_BP	GO:0018105	peptidyl-serine phosphorylation	9	*DMPK*	-0.87	7.85E+01	-2.33
GOTERM_MF	GO:0003697	single-stranded DNA binding	9	*SSBP4*	0.55	3.33E+00	-2.33
GOTERM_MF	GO:0003697	single-stranded DNA binding	9	*HMGB1*	-0.52	3.33E+00	-2.33
GOTERM_MF	GO:0003697	single-stranded DNA binding	9	*HMGB2*	-0.59	3.33E+00	-2.33
GOTERM_MF	GO:0003697	single-stranded DNA binding	9	*MSH2*	-0.81	3.33E+00	-2.33
GOTERM_MF	GO:0003697	single-stranded DNA binding	9	*NEIL3*	-0.75	3.33E+00	-2.33
GOTERM_MF	GO:0003697	single-stranded DNA binding	9	*BRCA2*	-0.67	3.33E+00	-2.33
GOTERM_MF	GO:0003697	single-stranded DNA binding	9	*MCM10*	-0.76	3.33E+00	-2.33
GOTERM_MF	GO:0003697	single-stranded DNA binding	9	*MCM6*	-0.70	3.33E+00	-2.33
GOTERM_MF	GO:0003697	single-stranded DNA binding	9	*RPA3*	-0.82	3.33E+00	-2.33
GOTERM_BP	GO:0007052	mitotic spindle organization	6	*CCNB1*	-0.73	3.36E+00	-2.45
GOTERM_BP	GO:0007052	mitotic spindle organization	6	*SPC25*	-0.62	3.36E+00	-2.45
GOTERM_BP	GO:0007052	mitotic spindle organization	6	*AURKA*	-0.52	3.36E+00	-2.45
GOTERM_BP	GO:0007052	mitotic spindle organization	6	*NDC80*	-0.79	3.36E+00	-2.45
GOTERM_BP	GO:0007052	mitotic spindle organization	6	*AURKB*	-0.69	3.36E+00	-2.45
GOTERM_BP	GO:0007052	mitotic spindle organization	6	*STMN1*	-1.40	3.36E+00	-2.45
GOTERM_BP	GO:0007080	mitotic metaphase plate congression	6	*CCNB1*	-0.73	1.24E+01	-2.45
GOTERM_BP	GO:0007080	mitotic metaphase plate congression	6	*KIF2C*	-0.61	1.24E+01	-2.45
GOTERM_BP	GO:0007080	mitotic metaphase plate congression	6	*KIF22*	-0.55	1.24E+01	-2.45
GOTERM_BP	GO:0007080	mitotic metaphase plate congression	6	*CDCA8*	-0.67	1.24E+01	-2.45
GOTERM_BP	GO:0007080	mitotic metaphase plate congression	6	*KIF18A*	-0.69	1.24E+01	-2.45
GOTERM_BP	GO:0007080	mitotic metaphase plate congression	6	*SPDL1*	-0.78	1.24E+01	-2.45

GOTERM_MF	GO:0042393	histone binding	6	*HMGB1*	-0.52	3.65E+01	-2.45
GOTERM_MF	GO:0042393	histone binding	6	*HMGB2*	-0.59	3.65E+01	-2.45
GOTERM_MF	GO:0042393	histone binding	6	*ANP32E*	-0.64	3.65E+01	-2.45
GOTERM_MF	GO:0042393	histone binding	6	*CKS2*	-0.50	3.65E+01	-2.45
GOTERM_MF	GO:0042393	histone binding	6	*HAT1*	-0.53	3.65E+01	-2.45
GOTERM_MF	GO:0042393	histone binding	6	*LOC504599*	-0.79	3.65E+01	-2.45
KEGG_PATHWAY	bta03430	Mismatch repair	6	*EXO1*	-0.70	3.35E+00	-2.45
KEGG_PATHWAY	bta03430	Mismatch repair	6	*RFC4*	-0.56	3.35E+00	-2.45
KEGG_PATHWAY	bta03430	Mismatch repair	6	*RFC2*	-0.59	3.35E+00	-2.45
KEGG_PATHWAY	bta03430	Mismatch repair	6	*MSH2*	-0.81	3.35E+00	-2.45
KEGG_PATHWAY	bta03430	Mismatch repair	6	*PCNA*	-0.78	3.35E+00	-2.45
KEGG_PATHWAY	bta03430	Mismatch repair	6	*RPA3*	-0.82	3.35E+00	-2.45
GOTERM_BP	GO:0000724	double-strand break repair via homologous recombination	7	*MCM8*	-0.61	3.81E+01	-2.65
GOTERM_BP	GO:0000724	double-strand break repair via homologous recombination	7	*MMS22L*	-0.54	3.81E+01	-2.65
GOTERM_BP	GO:0000724	double-strand break repair via homologous recombination	7	*GEN1*	-0.78	3.81E+01	-2.65
GOTERM_BP	GO:0000724	double-strand break repair via homologous recombination	7	*BRCA2*	-0.67	3.81E+01	-2.65
GOTERM_BP	GO:0000724	double-strand break repair via homologous recombination	7	*RAD54L*	-0.54	3.81E+01	-2.65
GOTERM_BP	GO:0000724	double-strand break repair via homologous recombination	7	*BRCA1*	-0.56	3.81E+01	-2.65
GOTERM_BP	GO:0000724	double-strand break repair via homologous recombination	7	*RPA3*	-0.82	3.81E+01	-2.65

GOTERM_CC	GO:0000777	condensed chromosome kinetochore	7	*SPC24*	-0.56		5.67E+00	-2.65
GOTERM_CC	GO:0000777	condensed chromosome kinetochore	7	*SPC25*	-0.62		5.67E+00	-2.65
GOTERM_CC	GO:0000777	condensed chromosome kinetochore	7	*CENPN*	-0.62		5.67E+00	-2.65
GOTERM_CC	GO:0000777	condensed chromosome kinetochore	7	*CENPM*	-0.95		5.67E+00	-2.65
GOTERM_CC	GO:0000777	condensed chromosome kinetochore	7	*APITD1*	-0.76		5.67E+00	-2.65
GOTERM_CC	GO:0000777	condensed chromosome kinetochore	7	*ZWILCH*	-0.65		5.67E+00	-2.65
GOTERM_CC	GO:0000777	condensed chromosome kinetochore	7	*ERCC6L*	-0.78		5.67E+00	-2.65
GOTERM_CC	GO:0000788	nuclear nucleosome	7	*HIST1H2BB*	-0.64		1.14E+01	-2.65
GOTERM_CC	GO:0000788	nuclear nucleosome	7	*HIST1H2BM*	-0.53		1.14E+01	-2.65
GOTERM_CC	GO:0000788	nuclear nucleosome	7	*HIST1H2BN*	-0.72		1.14E+01	-2.65
GOTERM_CC	GO:0000788	nuclear nucleosome	7	*HIST1H2BD*	-0.59		1.14E+01	-2.65
GOTERM_CC	GO:0000788	nuclear nucleosome	7	*LOC787465*	-0.71		1.14E+01	-2.65
GOTERM_CC	GO:0000788	nuclear nucleosome	7	*LOC505183*	-0.88		1.14E+01	-2.65
GOTERM_CC	GO:0000788	nuclear nucleosome	7	*LOC616819*	-0.75		1.14E+01	-2.65
KEGG_PATHWAY	bta03410	Base excision repair	7	*HMGB1*	-0.52		3.33E+00	-2.65
KEGG_PATHWAY	bta03410	Base excision repair	7	*NEIL3*	-0.75		3.33E+00	-2.65
KEGG_PATHWAY	bta03410	Base excision repair	7	*UNG*	-0.57		3.33E+00	-2.65
KEGG_PATHWAY	bta03410	Base excision repair	7	*POLE*	-0.66		3.33E+00	-2.65
KEGG_PATHWAY	bta03410	Base excision repair	7	*PCNA*	-0.78		3.33E+00	-2.65
KEGG_PATHWAY	bta03410	Base excision repair	7	*APEX1*	-0.50		3.33E+00	-2.65
KEGG_PATHWAY	bta03410	Base excision repair	7	*FEN1*	-0.55		3.33E+00	-2.65
GOTERM_BP	GO:0006270	DNA replication initiation	8	*CCNE2*	-0.74	0.000	2.13E-02	-2.83
GOTERM_BP	GO:0006270	DNA replication initiation	8	*CDC6*	-0.76	0.000	2.13E-02	-2.83
GOTERM_BP	GO:0006270	DNA replication initiation	8	*CCNE1*	-0.57	0.000	2.13E-02	-2.83
GOTERM_BP	GO:0006270	DNA replication initiation	8	*TOPBP1*	-0.54	0.000	2.13E-02	-2.83

GOTERM_BP	GO:0006270	DNA replication initiation	8	*MCM3*	-0.67	0.000	2.13E-02	-2.83
GOTERM_BP	GO:0006270	DNA replication initiation	8	*MCM10*	-0.76	0.000	2.13E-02	-2.83
GOTERM_BP	GO:0006270	DNA replication initiation	8	*MCM5*	-0.51	0.000	2.13E-02	-2.83
GOTERM_BP	GO:0006270	DNA replication initiation	8	*MCM6*	-0.70	0.000	2.13E-02	-2.83
GOTERM_BP	GO:0007059	chromosome segregation	8	*SPC25*	-0.62	0.002	3.99E+00	-2.83
GOTERM_BP	GO:0007059	chromosome segregation	8	*CENPN*	-0.62	0.002	3.99E+00	-2.83
GOTERM_BP	GO:0007059	chromosome segregation	8	*KIF11*	-0.94	0.002	3.99E+00	-2.83
GOTERM_BP	GO:0007059	chromosome segregation	8	*OIP5*	-0.69	0.002	3.99E+00	-2.83
GOTERM_BP	GO:0007059	chromosome segregation	8	*CENPW*	-1.27	0.002	3.99E+00	-2.83
GOTERM_BP	GO:0007059	chromosome segregation	8	*SKA2*	-0.67	0.002	3.99E+00	-2.83
GOTERM_BP	GO:0007059	chromosome segregation	8	*SKA1*	-0.81	0.002	3.99E+00	-2.83
GOTERM_BP	GO:0007059	chromosome segregation	8	*BRCA1*	-0.56	0.002	3.99E+00	-2.83
GOTERM_CC	GO:0005876	spindle microtubule	8	*CDK1*	-0.54		6.48E-01	-2.83
GOTERM_CC	GO:0005876	spindle microtubule	8	*KIF11*	-0.94		6.48E-01	-2.83
GOTERM_CC	GO:0005876	spindle microtubule	8	*SPAG5*	-0.73		6.48E-01	-2.83
GOTERM_CC	GO:0005876	spindle microtubule	8	*NUSAP1*	-0.88		6.48E-01	-2.83
GOTERM_CC	GO:0005876	spindle microtubule	8	*AURKA*	-0.52		6.48E-01	-2.83
GOTERM_CC	GO:0005876	spindle microtubule	8	*SKA2*	-0.67		6.48E-01	-2.83
GOTERM_CC	GO:0005876	spindle microtubule	8	*AURKB*	-0.69		6.48E-01	-2.83
GOTERM_CC	GO:0005876	spindle microtubule	8	*SKA1*	-0.81		6.48E-01	-2.83
GOTERM_CC	GO:0000776	kinetochore	8	*KIF2C*	-0.61		1.44E+01	-2.83
GOTERM_CC	GO:0000776	kinetochore	8	*SPAG5*	-0.73		1.44E+01	-2.83
GOTERM_CC	GO:0000776	kinetochore	8	*KIF18A*	-0.69		1.44E+01	-2.83
GOTERM_CC	GO:0000776	kinetochore	8	*MTBP*	-0.64		1.44E+01	-2.83
GOTERM_CC	GO:0000776	kinetochore	8	*CENPF*	-0.82		1.44E+01	-2.83

GOTERM_CC	GO:0000776	kinetochore	8	*CENPW*	-1.27	1.44E+01	-2.83
GOTERM_CC	GO:0000776	kinetochore	8	*CENPE*	-0.75	1.44E+01	-2.83
GOTERM_CC	GO:0000776	kinetochore	8	*AURKB*	-0.69	1.44E+01	-2.83
KEGG_PATHWAY	bta03460	Fanconi anemia pathway	8	*APITD1*	-0.76	8.39E+00	-2.83
KEGG_PATHWAY	bta03460	Fanconi anemia pathway	8	*FANCD2*	-0.91	8.39E+00	-2.83
KEGG_PATHWAY	bta03460	Fanconi anemia pathway	8	*BRIP1*	-0.73	8.39E+00	-2.83
KEGG_PATHWAY	bta03460	Fanconi anemia pathway	8	*BRCA2*	-0.67	8.39E+00	-2.83
KEGG_PATHWAY	bta03460	Fanconi anemia pathway	8	*RMI2*	-1.46	8.39E+00	-2.83
KEGG_PATHWAY	bta03460	Fanconi anemia pathway	8	*FANCB*	-0.66	8.39E+00	-2.83
KEGG_PATHWAY	bta03460	Fanconi anemia pathway	8	*BRCA1*	-0.56	8.39E+00	-2.83
KEGG_PATHWAY	bta03460	Fanconi anemia pathway	8	*RPA3*	-0.82	8.39E+00	-2.83
KEGG_PATHWAY	bta05203	Viral carcinogenesis	19	*HIST1H2BB*	-0.64	7.59E+00	-2.98
KEGG_PATHWAY	bta05203	Viral carcinogenesis	19	*CDK1*	-0.54	7.59E+00	-2.98
KEGG_PATHWAY	bta05203	Viral carcinogenesis	19	*LOC516742*	-0.69	7.59E+00	-2.98
KEGG_PATHWAY	bta05203	Viral carcinogenesis	19	*HIST1H2BD*	-0.59	7.59E+00	-2.98
KEGG_PATHWAY	bta05203	Viral carcinogenesis	19	*LOC527388*	-0.62	7.59E+00	-2.98
KEGG_PATHWAY	bta05203	Viral carcinogenesis	19	*ACTN2*	-1.05	7.59E+00	-2.98
KEGG_PATHWAY	bta05203	Viral carcinogenesis	19	*LOC528329*	-0.96	7.59E+00	-2.98
KEGG_PATHWAY	bta05203	Viral carcinogenesis	19	*CCNE2*	-0.74	7.59E+00	-2.98
KEGG_PATHWAY	bta05203	Viral carcinogenesis	19	*CCNE1*	-0.57	7.59E+00	-2.98
KEGG_PATHWAY	bta05203	Viral carcinogenesis	19	*HIST1H2BM*	-0.53	7.59E+00	-2.98
KEGG_PATHWAY	bta05203	Viral carcinogenesis	19	*HIST1H2BN*	-0.72	7.59E+00	-2.98
KEGG_PATHWAY	bta05203	Viral carcinogenesis	19	*CCR3*	1.20	7.59E+00	-2.98
KEGG_PATHWAY	bta05203	Viral carcinogenesis	19	*IRF7*	0.54	7.59E+00	-2.98
KEGG_PATHWAY	bta05203	Viral carcinogenesis	19	*JUN*	0.57	7.59E+00	-2.98

KEGG_PATHWAY	bta05203	Viral carcinogenesis	19	*HIST2H2BF*	-0.78	7.59E+00	-2.98
KEGG_PATHWAY	bta05203	Viral carcinogenesis	19	*LOC505183*	-0.88	7.59E+00	-2.98
KEGG_PATHWAY	bta05203	Viral carcinogenesis	19	*LOC787465*	-0.71	7.59E+00	-2.98
KEGG_PATHWAY	bta05203	Viral carcinogenesis	19	*CCNA2*	-0.67	7.59E+00	-2.98
KEGG_PATHWAY	bta05203	Viral carcinogenesis	19	*ATP6V0D2*	-1.14	7.59E+00	-2.98
GOTERM_BP	GO:0006260	DNA replication	9	*MCM8*	-0.61	3.80E+00	-3.00
GOTERM_BP	GO:0006260	DNA replication	9	*TICRR*	-0.54	3.80E+00	-3.00
GOTERM_BP	GO:0006260	DNA replication	9	*NASP*	-0.51	3.80E+00	-3.00
GOTERM_BP	GO:0006260	DNA replication	9	*POLE*	-0.66	3.80E+00	-3.00
GOTERM_BP	GO:0006260	DNA replication	9	*KIAA0101*	-0.78	3.80E+00	-3.00
GOTERM_BP	GO:0006260	DNA replication	9	*RMI2*	-1.46	3.80E+00	-3.00
GOTERM_BP	GO:0006260	DNA replication	9	*ORC1*	-0.89	3.80E+00	-3.00
GOTERM_BP	GO:0006260	DNA replication	9	*BRCA1*	-0.56	3.80E+00	-3.00
GOTERM_BP	GO:0006260	DNA replication	9	*RPA3*	-0.82	3.80E+00	-3.00
GOTERM_CC	GO:0000922	spindle pole	9	*CCNB1*	-0.73	1.49E+01	-3.00
GOTERM_CC	GO:0000922	spindle pole	9	*CDC6*	-0.76	1.49E+01	-3.00
GOTERM_CC	GO:0000922	spindle pole	9	*KIF11*	-0.94	1.49E+01	-3.00
GOTERM_CC	GO:0000922	spindle pole	9	*CKAP2L*	-0.55	1.49E+01	-3.00
GOTERM_CC	GO:0000922	spindle pole	9	*TPX2*	-0.71	1.49E+01	-3.00
GOTERM_CC	GO:0000922	spindle pole	9	*KNTC1*	-0.74	1.49E+01	-3.00
GOTERM_CC	GO:0000922	spindle pole	9	*CENPF*	-0.82	1.49E+01	-3.00
GOTERM_CC	GO:0000922	spindle pole	9	*AURKA*	-0.52	1.49E+01	-3.00
GOTERM_CC	GO:0000922	spindle pole	9	*SPDL1*	-0.78	1.49E+01	-3.00
GOTERM_CC	GO:0030496	midbody	9	*CDK1*	-0.54	5.48E+01	-3.00
GOTERM_CC	GO:0030496	midbody	9	*CDCA8*	-0.67	5.48E+01	-3.00

GOTERM_CC	GO:0030496	midbody	9	*PRC1*	-0.54	5.48E+01	-3.00
GOTERM_CC	GO:0030496	midbody	9	*CENPF*	-0.82	5.48E+01	-3.00
GOTERM_CC	GO:0030496	midbody	9	*CENPE*	-0.75	5.48E+01	-3.00
GOTERM_CC	GO:0030496	midbody	9	*CEP55*	-0.65	5.48E+01	-3.00
GOTERM_CC	GO:0030496	midbody	9	*AURKB*	-0.69	5.48E+01	-3.00
GOTERM_CC	GO:0030496	midbody	9	*ECT2*	-0.53	5.48E+01	-3.00
GOTERM_CC	GO:0030496	midbody	9	*ASPM*	-0.68	5.48E+01	-3.00
GOTERM_MF	GO:0003684	damaged DNA binding	9	*HMGB1*	-0.52	8.42E-01	-3.00
GOTERM_MF	GO:0003684	damaged DNA binding	9	*HMGB2*	-0.59	8.42E-01	-3.00
GOTERM_MF	GO:0003684	damaged DNA binding	9	*MSH2*	-0.81	8.42E-01	-3.00
GOTERM_MF	GO:0003684	damaged DNA binding	9	*NEIL3*	-0.75	8.42E-01	-3.00
GOTERM_MF	GO:0003684	damaged DNA binding	9	*UNG*	-0.57	8.42E-01	-3.00
GOTERM_MF	GO:0003684	damaged DNA binding	9	*PCNA*	-0.78	8.42E-01	-3.00
GOTERM_MF	GO:0003684	damaged DNA binding	9	*APEX1*	-0.50	8.42E-01	-3.00
GOTERM_MF	GO:0003684	damaged DNA binding	9	*BRCA1*	-0.56	8.42E-01	-3.00
GOTERM_MF	GO:0003684	damaged DNA binding	9	*RPA3*	-0.82	8.42E-01	-3.00
KEGG_PATHWAY	bta03030	DNA replication	9	*RFC4*	-0.56	1.55E-01	-3.00
KEGG_PATHWAY	bta03030	DNA replication	9	*RFC2*	-0.59	1.55E-01	-3.00
KEGG_PATHWAY	bta03030	DNA replication	9	*POLE*	-0.66	1.55E-01	-3.00
KEGG_PATHWAY	bta03030	DNA replication	9	*PCNA*	-0.78	1.55E-01	-3.00
KEGG_PATHWAY	bta03030	DNA replication	9	*MCM3*	-0.67	1.55E-01	-3.00
KEGG_PATHWAY	bta03030	DNA replication	9	*MCM5*	-0.51	1.55E-01	-3.00
KEGG_PATHWAY	bta03030	DNA replication	9	*FEN1*	-0.55	1.55E-01	-3.00
KEGG_PATHWAY	bta03030	DNA replication	9	*MCM6*	-0.70	1.55E-01	-3.00
KEGG_PATHWAY	bta03030	DNA replication	9	*RPA3*	-0.82	1.55E-01	-3.00

GOTERM_CC	GO:0000786	nucleosome	10	*HIST1H2AC*	-0.59	4.04E-01	-3.16
GOTERM_CC	GO:0000786	nucleosome	10	*HIST1H1E*	-0.54	4.04E-01	-3.16
GOTERM_CC	GO:0000786	nucleosome	10	*HIST1H1D*	-0.55	4.04E-01	-3.16
GOTERM_CC	GO:0000786	nucleosome	10	*HIST1H2AG*	-0.84	4.04E-01	-3.16
GOTERM_CC	GO:0000786	nucleosome	10	*HIST1H1A*	-1.47	4.04E-01	-3.16
GOTERM_CC	GO:0000786	nucleosome	10	*H2AFZ*	-0.60	4.04E-01	-3.16
GOTERM_CC	GO:0000786	nucleosome	10	*DCK*	-0.74	4.04E-01	-3.16
GOTERM_CC	GO:0000786	nucleosome	10	*HIST1H2AJ*	-1.05	4.04E-01	-3.16
GOTERM_CC	GO:0000786	nucleosome	10	*LOC616819*	-0.75	4.04E-01	-3.16
GOTERM_CC	GO:0000786	nucleosome	10	*LOC504599*	-0.79	4.04E-01	-3.16
GOTERM_CC	GO:0000784	nuclear chromosome telomeric region	11	*CDK1*	-0.54	4.01E+00	-3.32
GOTERM_CC	GO:0000784	nuclear chromosome telomeric region	11	*MSH2*	-0.81	4.01E+00	-3.32
GOTERM_CC	GO:0000784	nuclear chromosome telomeric region	11	*PCNA*	-0.78	4.01E+00	-3.32
GOTERM_CC	GO:0000784	nuclear chromosome telomeric region	11	*HAT1*	-0.53	4.01E+00	-3.32
GOTERM_CC	GO:0000784	nuclear chromosome telomeric region	11	*BRCA2*	-0.67	4.01E+00	-3.32
GOTERM_CC	GO:0000784	nuclear chromosome telomeric region	11	*MCM3*	-0.67	4.01E+00	-3.32
GOTERM_CC	GO:0000784	nuclear chromosome telomeric region	11	*ORC1*	-0.90	4.01E+00	-3.32
GOTERM_CC	GO:0000784	nuclear chromosome telomeric region	11	*LOC616819*	-0.76	4.01E+00	-3.32
GOTERM_CC	GO:0000784	nuclear chromosome telomeric region	11	*MCM5*	-0.51	4.01E+00	-3.32
GOTERM_CC	GO:0000784	nuclear chromosome telomeric region	11	*FEN1*	-0.55	4.01E+00	-3.32
GOTERM_CC	GO:0000784	nuclear chromosome telomeric region	11	*MCM6*	-0.70	4.01E+00	-3.32
KEGG_PATHWAY	bta05034	Alcoholism	22	*LOC516742*	-0.69	6.69E-02	-3.84
KEGG_PATHWAY	bta05034	Alcoholism	22	*HIST1H2BB*	-0.64	6.69E-02	-3.84
KEGG_PATHWAY	bta05034	Alcoholism	22	*HIST1H2AC*	-0.59	6.69E-02	-3.84
KEGG_PATHWAY	bta05034	Alcoholism	22	*HIST1H2BD*	-0.59	6.69E-02	-3.84

KEGG_PATHWAY	bta05034	Alcoholism	22	*HIST1H2AG*	-0.84	6.69E-02	-3.84
KEGG_PATHWAY	bta05034	Alcoholism	22	*MAOB*	1.08	6.69E-02	-3.84
KEGG_PATHWAY	bta05034	Alcoholism	22	*HAT1*	-0.53	6.69E-02	-3.84
KEGG_PATHWAY	bta05034	Alcoholism	22	*LOC527388*	-0.62	6.69E-02	-3.84
KEGG_PATHWAY	bta05034	Alcoholism	22	*PKIA*	-1.54	6.69E-02	-3.84
KEGG_PATHWAY	bta05034	Alcoholism	22	*LOC528329*	-0.96	6.69E-02	-3.84
KEGG_PATHWAY	bta05034	Alcoholism	22	*LOC616819*	-0.75	6.69E-02	-3.84
KEGG_PATHWAY	bta05034	Alcoholism	22	*LOC504599*	-0.79	6.69E-02	-3.84
KEGG_PATHWAY	bta05034	Alcoholism	22	*CAMKK1*	0.74	6.69E-02	-3.84
KEGG_PATHWAY	bta05034	Alcoholism	22	*HIST1H2BM*	-0.53	6.69E-02	-3.84
KEGG_PATHWAY	bta05034	Alcoholism	22	*HIST1H2BN*	-0.72	6.69E-02	-3.84
KEGG_PATHWAY	bta05034	Alcoholism	22	*HIST2H2BF*	-0.78	6.69E-02	-3.84
KEGG_PATHWAY	bta05034	Alcoholism	22	*NTRK2*	-1.48	6.69E-02	-3.84
KEGG_PATHWAY	bta05034	Alcoholism	22	*H2AFZ*	-0.60	6.69E-02	-3.84
KEGG_PATHWAY	bta05034	Alcoholism	22	*HIST1H2AH*	-0.66	6.69E-02	-3.84
KEGG_PATHWAY	bta05034	Alcoholism	22	*HIST1H2AJ*	-1.05	6.69E-02	-3.84
KEGG_PATHWAY	bta05034	Alcoholism	22	*LOC505183*	-0.88	6.69E-02	-3.84
KEGG_PATHWAY	bta05034	Alcoholism	22	*LOC787465*	-0.71	6.69E-02	-3.84
GOTERM_BP	GO:0006334	nucleosome assembly	15	*HMGB1*	-0.52	7.87E-02	-3.87
GOTERM_BP	GO:0006334	nucleosome assembly	15	*HIST1H2BB*	-0.64	7.87E-02	-3.87
GOTERM_BP	GO:0006334	nucleosome assembly	15	*HIST1H1E*	-0.54	7.87E-02	-3.87
GOTERM_BP	GO:0006334	nucleosome assembly	15	*HIST1H1D*	-0.55	7.87E-02	-3.87
GOTERM_BP	GO:0006334	nucleosome assembly	15	*HIST1H2BD*	-0.59	7.87E-02	-3.87
GOTERM_BP	GO:0006334	nucleosome assembly	15	*HIST1H1A*	-1.47	7.87E-02	-3.87
GOTERM_BP	GO:0006334	nucleosome assembly	15	*ANP32E*	-0.64	7.87E-02	-3.87

GOTERM_BP	GO:0006334	nucleosome assembly	15	*NAP1L1*	-0.55		7.87E-02	-3.87
GOTERM_BP	GO:0006334	nucleosome assembly	15	*DCK*	-0.74		7.87E-02	-3.87
GOTERM_BP	GO:0006334	nucleosome assembly	15	*LOC616819*	-0.75		7.87E-02	-3.87
GOTERM_BP	GO:0006334	nucleosome assembly	15	*LOC504599*	-0.79		7.87E-02	-3.87
GOTERM_BP	GO:0006334	nucleosome assembly	15	*HIST1H2BM*	-0.53		7.87E-02	-3.87
GOTERM_BP	GO:0006334	nucleosome assembly	15	*HIST1H2BN*	-0.72		7.87E-02	-3.87
GOTERM_BP	GO:0006334	nucleosome assembly	15	*LOC505183*	-0.88		7.87E-02	-3.87
GOTERM_BP	GO:0006334	nucleosome assembly	15	*LOC787465*	-0.71		7.87E-02	-3.87
GOTERM_BP	GO:0007067	mitotic nuclear division	18	*CENPN*	-0.62	0.000	1.76E-03	-4.24
GOTERM_BP	GO:0007067	mitotic nuclear division	18	*CDK1*	-0.54	0.000	1.76E-03	-4.24
GOTERM_BP	GO:0007067	mitotic nuclear division	18	*NUF2*	-0.76	0.000	1.76E-03	-4.24
GOTERM_BP	GO:0007067	mitotic nuclear division	18	*SPC24*	-0.56	0.000	1.76E-03	-4.24
GOTERM_BP	GO:0007067	mitotic nuclear division	18	*FAM83D*	-0.51	0.000	1.76E-03	-4.24
GOTERM_BP	GO:0007067	mitotic nuclear division	18	*SPC25*	-0.62	0.000	1.76E-03	-4.24
GOTERM_BP	GO:0007067	mitotic nuclear division	18	*VRK1*	-0.58	0.000	1.76E-03	-4.24
GOTERM_BP	GO:0007067	mitotic nuclear division	18	*CCNB2*	-0.73	0.000	1.76E-03	-4.24
GOTERM_BP	GO:0007067	mitotic nuclear division	18	*APITD1*	-0.76	0.000	1.76E-03	-4.24
GOTERM_BP	GO:0007067	mitotic nuclear division	18	*CDCA2*	-0.59	0.000	1.76E-03	-4.24
GOTERM_BP	GO:0007067	mitotic nuclear division	18	*CENPW*	-1.27	0.000	1.76E-03	-4.24
GOTERM_BP	GO:0007067	mitotic nuclear division	18	*SKA2*	-0.67	0.000	1.76E-03	-4.24
GOTERM_BP	GO:0007067	mitotic nuclear division	18	*MASTL*	-0.60	0.000	1.76E-03	-4.24
GOTERM_BP	GO:0007067	mitotic nuclear division	18	*SKA1*	-0.81	0.000	1.76E-03	-4.24
GOTERM_BP	GO:0007067	mitotic nuclear division	18	*ZWILCH*	-0.65	0.000	1.76E-03	-4.24
GOTERM_BP	GO:0007067	mitotic nuclear division	18	*CCNA2*	-0.67	0.000	1.76E-03	-4.24
GOTERM_BP	GO:0007067	mitotic nuclear division	18	*ASPM*	-0.68	0.000	1.76E-03	-4.24

GOTERM_BP	GO:0007067	mitotic nuclear division	18	*ERCC6L*	-0.78	0.000	1.76E-03	-4.24
KEGG_PATHWAY	bta04110	Cell cycle	18	*CDC6*	-0.76	0.000	3.21E-02	-4.24
KEGG_PATHWAY	bta04110	Cell cycle	18	*CDK1*	-0.54	0.000	3.21E-02	-4.24
KEGG_PATHWAY	bta04110	Cell cycle	18	*DBF4*	-0.51	0.000	3.21E-02	-4.24
KEGG_PATHWAY	bta04110	Cell cycle	18	*PKMYT1*	-0.76	0.000	3.21E-02	-4.24
KEGG_PATHWAY	bta04110	Cell cycle	18	*MCM3*	-0.67	0.000	3.21E-02	-4.24
KEGG_PATHWAY	bta04110	Cell cycle	18	*MCM5*	-0.51	0.000	3.21E-02	-4.24
KEGG_PATHWAY	bta04110	Cell cycle	18	*MCM6*	-0.70	0.000	3.21E-02	-4.24
KEGG_PATHWAY	bta04110	Cell cycle	18	*CCNE2*	-0.74	0.000	3.21E-02	-4.24
KEGG_PATHWAY	bta04110	Cell cycle	18	*CCNB1*	-0.73	0.000	3.21E-02	-4.24
KEGG_PATHWAY	bta04110	Cell cycle	18	*CCNE1*	-0.57	0.000	3.21E-02	-4.24
KEGG_PATHWAY	bta04110	Cell cycle	18	*CCNB2*	-0.73	0.000	3.21E-02	-4.24
KEGG_PATHWAY	bta04110	Cell cycle	18	*MAD2L1*	-0.66	0.000	3.21E-02	-4.24
KEGG_PATHWAY	bta04110	Cell cycle	18	*CDKN2C*	-0.65	0.000	3.21E-02	-4.24
KEGG_PATHWAY	bta04110	Cell cycle	18	*BUB1*	-0.71	0.000	3.21E-02	-4.24
KEGG_PATHWAY	bta04110	Cell cycle	18	*PCNA*	-0.78	0.000	3.21E-02	-4.24
KEGG_PATHWAY	bta04110	Cell cycle	18	*BUB1B*	-0.69	0.000	3.21E-02	-4.24
KEGG_PATHWAY	bta04110	Cell cycle	18	*CCNA2*	-0.67	0.000	3.21E-02	-4.24
KEGG_PATHWAY	bta04110	Cell cycle	18	*ORC1*	-0.89	0.000	3.21E-02	-4.24
GOTERM_MF	GO:0005524	ATP binding	76	*KIFC2*	0.50	0.000	9.73E-02	-4.59
GOTERM_MF	GO:0005524	ATP binding	76	*KIF22*	-0.55	0.000	9.73E-02	-4.59
GOTERM_MF	GO:0005524	ATP binding	76	*KIF24*	-0.55	0.000	9.73E-02	-4.59
GOTERM_MF	GO:0005524	ATP binding	76	*MYO7A*	0.59	0.000	9.73E-02	-4.59
GOTERM_MF	GO:0005524	ATP binding	76	*DTYMK*	-0.57	0.000	9.73E-02	-4.59
GOTERM_MF	GO:0005524	ATP binding	76	*PKMYT1*	-0.76	0.000	9.73E-02	-4.59

GOTERM_MF	GO:0005524	ATP binding	76	*AURKA*	-0.52	0.000	9.73E-02	-4.59
GOTERM_MF	GO:0005524	ATP binding	76	*AURKB*	-0.69	0.000	9.73E-02	-4.59
GOTERM_MF	GO:0005524	ATP binding	76	*CAMKK1*	0.74	0.000	9.73E-02	-4.59
GOTERM_MF	GO:0005524	ATP binding	76	*KIFC3*	0.77	0.000	9.73E-02	-4.59
GOTERM_MF	GO:0005524	ATP binding	76	*DMPK*	-0.87	0.000	9.73E-02	-4.59
GOTERM_MF	GO:0005524	ATP binding	76	*ACTG2*	-1.39	0.000	9.73E-02	-4.59
GOTERM_MF	GO:0005524	ATP binding	76	*KIF2C*	-0.61	0.000	9.73E-02	-4.59
GOTERM_MF	GO:0005524	ATP binding	76	*MCM8*	-0.61	0.000	9.73E-02	-4.59
GOTERM_MF	GO:0005524	ATP binding	76	*MCCC1*	-0.50	0.000	9.73E-02	-4.59
GOTERM_MF	GO:0005524	ATP binding	76	*WNK4*	1.02	0.000	9.73E-02	-4.59
GOTERM_MF	GO:0005524	ATP binding	76	*MASTL*	-0.60	0.000	9.73E-02	-4.59
GOTERM_MF	GO:0005524	ATP binding	76	*ORC1*	-0.89	0.000	9.73E-02	-4.59
GOTERM_MF	GO:0005524	ATP binding	76	*TOP2A*	-0.76	0.000	9.73E-02	-4.59
GOTERM_MF	GO:0005524	ATP binding	76	*NMNAT2*	0.67	0.000	9.73E-02	-4.59
GOTERM_MF	GO:0005524	ATP binding	76	*CDK1*	-0.54	0.000	9.73E-02	-4.59
GOTERM_MF	GO:0005524	ATP binding	76	*SGK1*	0.54	0.000	9.73E-02	-4.59
GOTERM_MF	GO:0005524	ATP binding	76	*KIF11*	-0.94	0.000	9.73E-02	-4.59
GOTERM_MF	GO:0005524	ATP binding	76	*PNCK*	-1.20	0.000	9.73E-02	-4.59
GOTERM_MF	GO:0005524	ATP binding	76	*KIF15*	-0.95	0.000	9.73E-02	-4.59
GOTERM_MF	GO:0005524	ATP binding	76	*CFTR*	-0.69	0.000	9.73E-02	-4.59
GOTERM_MF	GO:0005524	ATP binding	76	*PBK*	-0.67	0.000	9.73E-02	-4.59
GOTERM_MF	GO:0005524	ATP binding	76	*UBE2C*	-0.69	0.000	9.73E-02	-4.59
GOTERM_MF	GO:0005524	ATP binding	76	*MCM3*	-0.67	0.000	9.73E-02	-4.59
GOTERM_MF	GO:0005524	ATP binding	76	*MCM5*	-0.51	0.000	9.73E-02	-4.59
GOTERM_MF	GO:0005524	ATP binding	76	*MCM6*	-0.70	0.000	9.73E-02	-4.59

GOTERM_MF	GO:0005524	ATP binding	76	*TARS*	-1.19	0.000	9.73E-02	-4.59
GOTERM_MF	GO:0005524	ATP binding	76	*NME2*	-0.53	0.000	9.73E-02	-4.59
GOTERM_MF	GO:0005524	ATP binding	76	*RFC4*	-0.56	0.000	9.73E-02	-4.59
GOTERM_MF	GO:0005524	ATP binding	76	*RFC2*	-0.59	0.000	9.73E-02	-4.59
GOTERM_MF	GO:0005524	ATP binding	76	*BUB1B*	-0.69	0.000	9.73E-02	-4.59
GOTERM_MF	GO:0005524	ATP binding	76	*KIF19*	0.64	0.000	9.73E-02	-4.59
GOTERM_MF	GO:0005524	ATP binding	76	*LRRK2*	-0.58	0.000	9.73E-02	-4.59
GOTERM_MF	GO:0005524	ATP binding	76	*PRKD3*	-0.61	0.000	9.73E-02	-4.59
GOTERM_MF	GO:0005524	ATP binding	76	*MELK*	-0.69	0.000	9.73E-02	-4.59
GOTERM_MF	GO:0005524	ATP binding	76	*KIF4A*	-0.59	0.000	9.73E-02	-4.59
GOTERM_MF	GO:0005524	ATP binding	76	*PFKFB4*	0.96	0.000	9.73E-02	-4.59
GOTERM_MF	GO:0005524	ATP binding	76	*BLK*	-1.03	0.000	9.73E-02	-4.59
GOTERM_MF	GO:0005524	ATP binding	76	*PTK7*	0.53	0.000	9.73E-02	-4.59
GOTERM_MF	GO:0005524	ATP binding	76	*DCK*	-0.74	0.000	9.73E-02	-4.59
GOTERM_MF	GO:0005524	ATP binding	76	*NLRX1*	0.66	0.000	9.73E-02	-4.59
GOTERM_MF	GO:0005524	ATP binding	76	*HSPA1A*	0.77	0.000	9.73E-02	-4.59
GOTERM_MF	GO:0005524	ATP binding	76	*DNAH5*	-0.83	0.000	9.73E-02	-4.59
GOTERM_MF	GO:0005524	ATP binding	76	*VRK1*	-0.58	0.000	9.73E-02	-4.59
GOTERM_MF	GO:0005524	ATP binding	76	*CCT6B*	-0.66	0.000	9.73E-02	-4.59
GOTERM_MF	GO:0005524	ATP binding	76	*BUB1*	-0.71	0.000	9.73E-02	-4.59
GOTERM_MF	GO:0005524	ATP binding	76	*PAPSS2*	2.51	0.000	9.73E-02	-4.59
GOTERM_MF	GO:0005524	ATP binding	76	*DCLK1*	-0.94	0.000	9.73E-02	-4.59
GOTERM_MF	GO:0005524	ATP binding	76	*HELLS*	-0.80	0.000	9.73E-02	-4.59
GOTERM_MF	GO:0005524	ATP binding	76	*ERCC6L*	-0.78	0.000	9.73E-02	-4.59
GOTERM_MF	GO:0005524	ATP binding	76	*SWAP70*	-0.91	0.000	9.73E-02	-4.59

GOTERM_MF	GO:0005524	ATP binding	76	*MSH2*	-0.81	0.000	9.73E-02	-4.59
GOTERM_MF	GO:0005524	ATP binding	76	*PDK4*	0.96	0.000	9.73E-02	-4.59
GOTERM_MF	GO:0005524	ATP binding	76	*AXL*	0.52	0.000	9.73E-02	-4.59
GOTERM_MF	GO:0005524	ATP binding	76	*KIF18A*	-0.69	0.000	9.73E-02	-4.59
GOTERM_MF	GO:0005524	ATP binding	76	*BRIP1*	-0.73	0.000	9.73E-02	-4.59
GOTERM_MF	GO:0005524	ATP binding	76	*MAPK11*	1.10	0.000	9.73E-02	-4.59
GOTERM_MF	GO:0005524	ATP binding	76	*NPR2*	0.55	0.000	9.73E-02	-4.59
GOTERM_MF	GO:0005524	ATP binding	76	*ATAD5*	-0.64	0.000	9.73E-02	-4.59
GOTERM_MF	GO:0005524	ATP binding	76	*CENPE*	-0.75	0.000	9.73E-02	-4.59
GOTERM_MF	GO:0005524	ATP binding	76	*SMC2*	-0.71	0.000	9.73E-02	-4.59
GOTERM_MF	GO:0005524	ATP binding	76	*ATP13A2*	0.70	0.000	9.73E-02	-4.59
GOTERM_MF	GO:0005524	ATP binding	76	*RAD54L*	-0.54	0.000	9.73E-02	-4.59
GOTERM_MF	GO:0005524	ATP binding	76	*SMC4*	-0.58	0.000	9.73E-02	-4.59
GOTERM_MF	GO:0005524	ATP binding	76	*EPHA7*	-1.02	0.000	9.73E-02	-4.59
GOTERM_MF	GO:0005524	ATP binding	76	*PLK4*	-0.55	0.000	9.73E-02	-4.59
GOTERM_MF	GO:0005524	ATP binding	76	*NTRK2*	-1.48	0.000	9.73E-02	-4.59
GOTERM_MF	GO:0005524	ATP binding	76	*KIF20B*	-0.65	0.000	9.73E-02	-4.59
GOTERM_MF	GO:0005524	ATP binding	76	*MYH11*	-0.87	0.000	9.73E-02	-4.59
GOTERM_MF	GO:0005524	ATP binding	76	*PAICS*	-0.63	0.000	9.73E-02	-4.59
GOTERM_MF	GO:0005524	ATP binding	76	*MYLK*	-0.86	0.000	9.73E-02	-4.59
GOTERM_CC	GO:0005654	nucleoplasm	65	*CLSPN*	-0.73	0.083	6.95E+01	-4.59
GOTERM_CC	GO:0005654	nucleoplasm	65	*PRC1*	-0.54	0.083	6.95E+01	-4.59
GOTERM_CC	GO:0005654	nucleoplasm	65	*CNDP2*	0.68	0.083	6.95E+01	-4.59
GOTERM_CC	GO:0005654	nucleoplasm	65	*AURKB*	-0.69	0.083	6.95E+01	-4.59
GOTERM_CC	GO:0005654	nucleoplasm	65	*CCNE1*	-0.57	0.083	6.95E+01	-4.59

GOTERM_CC	GO:0005654	nucleoplasm	65	*FOS*	1.03	0.083	6.95E+01	-4.59
GOTERM_CC	GO:0005654	nucleoplasm	65	*HIST1H2BN*	-0.72	0.083	6.95E+01	-4.59
GOTERM_CC	GO:0005654	nucleoplasm	65	*CDCA7*	-0.59	0.083	6.95E+01	-4.59
GOTERM_CC	GO:0005654	nucleoplasm	65	*SLC25A22*	0.63	0.083	6.95E+01	-4.59
GOTERM_CC	GO:0005654	nucleoplasm	65	*MASTL*	-0.60	0.083	6.95E+01	-4.59
GOTERM_CC	GO:0005654	nucleoplasm	65	*HSF4*	0.55	0.083	6.95E+01	-4.59
GOTERM_CC	GO:0005654	nucleoplasm	65	*TOP2A*	-0.76	0.083	6.95E+01	-4.59
GOTERM_CC	GO:0005654	nucleoplasm	65	*CCNA2*	-0.67	0.083	6.95E+01	-4.59
GOTERM_CC	GO:0005654	nucleoplasm	65	*EGR1*	0.74	0.083	6.95E+01	-4.59
GOTERM_CC	GO:0005654	nucleoplasm	65	*CDC6*	-0.76	0.083	6.95E+01	-4.59
GOTERM_CC	GO:0005654	nucleoplasm	65	*EPN3*	-1.05	0.083	6.95E+01	-4.59
GOTERM_CC	GO:0005654	nucleoplasm	65	*SGK1*	0.54	0.083	6.95E+01	-4.59
GOTERM_CC	GO:0005654	nucleoplasm	65	*DTL*	-0.53	0.083	6.95E+01	-4.59
GOTERM_CC	GO:0005654	nucleoplasm	65	*NEIL3*	-0.75	0.083	6.95E+01	-4.59
GOTERM_CC	GO:0005654	nucleoplasm	65	*POLE*	-0.66	0.083	6.95E+01	-4.59
GOTERM_CC	GO:0005654	nucleoplasm	65	*TPX2*	-0.71	0.083	6.95E+01	-4.59
GOTERM_CC	GO:0005654	nucleoplasm	65	*RAI14*	-0.50	0.083	6.95E+01	-4.59
GOTERM_CC	GO:0005654	nucleoplasm	65	*MCM3*	-0.67	0.083	6.95E+01	-4.59
GOTERM_CC	GO:0005654	nucleoplasm	65	*GRHL1*	0.85	0.083	6.95E+01	-4.59
GOTERM_CC	GO:0005654	nucleoplasm	65	*MCM5*	-0.51	0.083	6.95E+01	-4.59
GOTERM_CC	GO:0005654	nucleoplasm	65	*MCM6*	-0.70	0.083	6.95E+01	-4.59
GOTERM_CC	GO:0005654	nucleoplasm	65	*JUN*	0.57	0.083	6.95E+01	-4.59
GOTERM_CC	GO:0005654	nucleoplasm	65	*RAD18*	-0.58	0.083	6.95E+01	-4.59
GOTERM_CC	GO:0005654	nucleoplasm	65	*LOC787465*	-0.71	0.083	6.95E+01	-4.59
GOTERM_CC	GO:0005654	nucleoplasm	65	*KPNA2*	-0.52	0.083	6.95E+01	-4.59

GOTERM_CC	GO:0005654	nucleoplasm	65	*PRKD3*	-0.61	0.083	6.95E+01	-4.59
GOTERM_CC	GO:0005654	nucleoplasm	65	*CDK5R1*	0.72	0.083	6.95E+01	-4.59
GOTERM_CC	GO:0005654	nucleoplasm	65	*LMNB1*	-0.67	0.083	6.95E+01	-4.59
GOTERM_CC	GO:0005654	nucleoplasm	65	*FKBP5*	-0.50	0.083	6.95E+01	-4.59
GOTERM_CC	GO:0005654	nucleoplasm	65	*TICRR*	-0.54	0.083	6.95E+01	-4.59
GOTERM_CC	GO:0005654	nucleoplasm	65	*ZNF367*	-0.56	0.083	6.95E+01	-4.59
GOTERM_CC	GO:0005654	nucleoplasm	65	*HAT1*	-0.53	0.083	6.95E+01	-4.59
GOTERM_CC	GO:0005654	nucleoplasm	65	*ANLN*	-0.53	0.083	6.95E+01	-4.59
GOTERM_CC	GO:0005654	nucleoplasm	65	*RRM2B*	-0.64	0.083	6.95E+01	-4.59
GOTERM_CC	GO:0005654	nucleoplasm	65	*MYBL2*	-1.02	0.083	6.95E+01	-4.59
GOTERM_CC	GO:0005654	nucleoplasm	65	*HIC1*	0.68	0.083	6.95E+01	-4.59
GOTERM_CC	GO:0005654	nucleoplasm	65	*VRK1*	-0.58	0.083	6.95E+01	-4.59
GOTERM_CC	GO:0005654	nucleoplasm	65	*BUB1*	-0.71	0.083	6.95E+01	-4.59
GOTERM_CC	GO:0005654	nucleoplasm	65	*BCL9L*	0.69	0.083	6.95E+01	-4.59
GOTERM_CC	GO:0005654	nucleoplasm	65	*LOC505183*	-0.88	0.083	6.95E+01	-4.59
GOTERM_CC	GO:0005654	nucleoplasm	65	*WDHD1*	-0.65	0.083	6.95E+01	-4.59
GOTERM_CC	GO:0005654	nucleoplasm	65	*APEX1*	-0.50	0.083	6.95E+01	-4.59
GOTERM_CC	GO:0005654	nucleoplasm	65	*FEN1*	-0.55	0.083	6.95E+01	-4.59
GOTERM_CC	GO:0005654	nucleoplasm	65	*ERCC6L*	-0.78	0.083	6.95E+01	-4.59
GOTERM_CC	GO:0005654	nucleoplasm	65	*EXO1*	-0.70	0.083	6.95E+01	-4.59
GOTERM_CC	GO:0005654	nucleoplasm	65	*HIST1H2BD*	-0.59	0.083	6.95E+01	-4.59
GOTERM_CC	GO:0005654	nucleoplasm	65	*CEBPE*	1.58	0.083	6.95E+01	-4.59
GOTERM_CC	GO:0005654	nucleoplasm	65	*CENPQ*	-0.60	0.083	6.95E+01	-4.59
GOTERM_CC	GO:0005654	nucleoplasm	65	*NASP*	-0.51	0.083	6.95E+01	-4.59
GOTERM_CC	GO:0005654	nucleoplasm	65	*BRCA2*	-0.67	0.083	6.95E+01	-4.59

GOTERM_CC	GO:0005654	nucleoplasm	65	*CENPF*	-0.82	0.083	6.95E+01	-4.59
GOTERM_CC	GO:0005654	nucleoplasm	65	*RAD54L*	-0.54	0.083	6.95E+01	-4.59
GOTERM_CC	GO:0005654	nucleoplasm	65	*SMC4*	-0.58	0.083	6.95E+01	-4.59
GOTERM_CC	GO:0005654	nucleoplasm	65	*IRF7*	0.54	0.083	6.95E+01	-4.59
GOTERM_CC	GO:0005654	nucleoplasm	65	*CAPG*	2.06	0.083	6.95E+01	-4.59
GOTERM_CC	GO:0005654	nucleoplasm	65	*PARPBP*	-0.59	0.083	6.95E+01	-4.59
GOTERM_CC	GO:0005654	nucleoplasm	65	*PCNA*	-0.78	0.083	6.95E+01	-4.59
GOTERM_CC	GO:0005654	nucleoplasm	65	*CD79B*	-3.11	0.083	6.95E+01	-4.59
GOTERM_CC	GO:0005654	nucleoplasm	65	*SNRNP25*	-0.56	0.083	6.95E+01	-4.59
GOTERM_CC	GO:0005654	nucleoplasm	65	*BARD1*	-0.68	0.083	6.95E+01	-4.59
GOTERM_CC	GO:0005634	nucleus	134	*C29H11ORF54*	-0.59	0.057	5.52E+01	-4.84
GOTERM_CC	GO:0005634	nucleus	134	*ALAD*	0.88	0.057	5.52E+01	-4.84
GOTERM_CC	GO:0005634	nucleus	134	*DBF4*	-0.51	0.057	5.52E+01	-4.84
GOTERM_CC	GO:0005634	nucleus	134	*DTYMK*	-0.57	0.057	5.52E+01	-4.84
GOTERM_CC	GO:0005634	nucleus	134	*PKMYT1*	-0.76	0.057	5.52E+01	-4.84
GOTERM_CC	GO:0005634	nucleus	134	*TCEAL8*	-0.63	0.057	5.52E+01	-4.84
GOTERM_CC	GO:0005634	nucleus	134	*AURKB*	-0.69	0.057	5.52E+01	-4.84
GOTERM_CC	GO:0005634	nucleus	134	*AQP3*	0.94	0.057	5.52E+01	-4.84
GOTERM_CC	GO:0005634	nucleus	134	*GLDC*	-1.18	0.057	5.52E+01	-4.84
GOTERM_CC	GO:0005634	nucleus	134	*CITED2*	0.55	0.057	5.52E+01	-4.84
GOTERM_CC	GO:0005634	nucleus	134	*CDCA7*	-0.59	0.057	5.52E+01	-4.84
GOTERM_CC	GO:0005634	nucleus	134	*GATA5*	0.52	0.057	5.52E+01	-4.84
GOTERM_CC	GO:0005634	nucleus	134	*CDKN2C*	-0.65	0.057	5.52E+01	-4.84
GOTERM_CC	GO:0005634	nucleus	134	*OIP5*	-0.69	0.057	5.52E+01	-4.84
GOTERM_CC	GO:0005634	nucleus	134	*WDR76*	-1.21	0.057	5.52E+01	-4.84

GOTERM_CC	GO:0005634	nucleus	134	*CDCA2*	-0.59	0.057	5.52E+01	-4.84
GOTERM_CC	GO:0005634	nucleus	134	*RARA*	0.54	0.057	5.52E+01	-4.84
GOTERM_CC	GO:0005634	nucleus	134	*MX1*	0.67	0.057	5.52E+01	-4.84
GOTERM_CC	GO:0005634	nucleus	134	*CCNA2*	-0.67	0.057	5.52E+01	-4.84
GOTERM_CC	GO:0005634	nucleus	134	*ASPM*	-0.68	0.057	5.52E+01	-4.84
GOTERM_CC	GO:0005634	nucleus	134	*POU2AF1*	-1.71	0.057	5.52E+01	-4.84
GOTERM_CC	GO:0005634	nucleus	134	*DYX1C1*	-0.89	0.057	5.52E+01	-4.84
GOTERM_CC	GO:0005634	nucleus	134	*NEIL3*	-0.75	0.057	5.52E+01	-4.84
GOTERM_CC	GO:0005634	nucleus	134	*MND1*	-0.62	0.057	5.52E+01	-4.84
GOTERM_CC	GO:0005634	nucleus	134	*RMI2*	-1.46	0.057	5.52E+01	-4.84
GOTERM_CC	GO:0005634	nucleus	134	*BASP1*	-0.53	0.057	5.52E+01	-4.84
GOTERM_CC	GO:0005634	nucleus	134	*GRHL1*	0.85	0.057	5.52E+01	-4.84
GOTERM_CC	GO:0005634	nucleus	134	*ELL3*	-3.87	0.057	5.52E+01	-4.84
GOTERM_CC	GO:0005634	nucleus	134	*PKIA*	-1.54	0.057	5.52E+01	-4.84
GOTERM_CC	GO:0005634	nucleus	134	*DPCD*	-0.60	0.057	5.52E+01	-4.84
GOTERM_CC	GO:0005634	nucleus	134	*TEX30*	-0.52	0.057	5.52E+01	-4.84
GOTERM_CC	GO:0005634	nucleus	134	*NME2*	-0.53	0.057	5.52E+01	-4.84
GOTERM_CC	GO:0005634	nucleus	134	*HSPB6*	-1.08	0.057	5.52E+01	-4.84
GOTERM_CC	GO:0005634	nucleus	134	*ZNF783*	0.53	0.057	5.52E+01	-4.84
GOTERM_CC	GO:0005634	nucleus	134	*RFC2*	-0.59	0.057	5.52E+01	-4.84
GOTERM_CC	GO:0005634	nucleus	134	*RAD18*	-0.58	0.057	5.52E+01	-4.84
GOTERM_CC	GO:0005634	nucleus	134	*SMPX*	-1.12	0.057	5.52E+01	-4.84
GOTERM_CC	GO:0005634	nucleus	134	*CDCA7L*	-0.52	0.057	5.52E+01	-4.84
GOTERM_CC	GO:0005634	nucleus	134	*NFE2L3*	-0.57	0.057	5.52E+01	-4.84
GOTERM_CC	GO:0005634	nucleus	134	*ASB4*	1.92	0.057	5.52E+01	-4.84

GOTERM_CC	GO:0005634	nucleus	134	*FGD2*	0.58	0.057	5.52E+01	-4.84
GOTERM_CC	GO:0005634	nucleus	134	*EID1*	-0.56	0.057	5.52E+01	-4.84
GOTERM_CC	GO:0005634	nucleus	134	*CDK5R1*	0.72	0.057	5.52E+01	-4.84
GOTERM_CC	GO:0005634	nucleus	134	*HMGB2*	-0.59	0.057	5.52E+01	-4.84
GOTERM_CC	GO:0005634	nucleus	134	*CAMTA2*	0.56	0.057	5.52E+01	-4.84
GOTERM_CC	GO:0005634	nucleus	134	*SESN2*	0.65	0.057	5.52E+01	-4.84
GOTERM_CC	GO:0005634	nucleus	134	*MYBL2*	-1.02	0.057	5.52E+01	-4.84
GOTERM_CC	GO:0005634	nucleus	134	*SPC24*	-0.56	0.057	5.52E+01	-4.84
GOTERM_CC	GO:0005634	nucleus	134	*SPC25*	-0.62	0.057	5.52E+01	-4.84
GOTERM_CC	GO:0005634	nucleus	134	*VRK1*	-0.58	0.057	5.52E+01	-4.84
GOTERM_CC	GO:0005634	nucleus	134	*HELLS*	-0.80	0.057	5.52E+01	-4.84
GOTERM_CC	GO:0005634	nucleus	134	*NR1H3*	0.63	0.057	5.52E+01	-4.84
GOTERM_CC	GO:0005634	nucleus	134	*IKZF2*	-0.62	0.057	5.52E+01	-4.84
GOTERM_CC	GO:0005634	nucleus	134	*KCTD1*	0.51	0.057	5.52E+01	-4.84
GOTERM_CC	GO:0005634	nucleus	134	*NUF2*	-0.76	0.057	5.52E+01	-4.84
GOTERM_CC	GO:0005634	nucleus	134	*BRIP1*	-0.73	0.057	5.52E+01	-4.84
GOTERM_CC	GO:0005634	nucleus	134	*NR4A1*	1.03	0.057	5.52E+01	-4.84
GOTERM_CC	GO:0005634	nucleus	134	*BRCA2*	-0.67	0.057	5.52E+01	-4.84
GOTERM_CC	GO:0005634	nucleus	134	*SPDL1*	-0.78	0.057	5.52E+01	-4.84
GOTERM_CC	GO:0005634	nucleus	134	*FOXP3*	1.07	0.057	5.52E+01	-4.84
GOTERM_CC	GO:0005634	nucleus	134	*BRCA1*	-0.56	0.057	5.52E+01	-4.84
GOTERM_CC	GO:0005634	nucleus	134	*NOTCH1*	0.59	0.057	5.52E+01	-4.84
GOTERM_CC	GO:0005634	nucleus	134	*DLX4*	1.05	0.057	5.52E+01	-4.84
GOTERM_CC	GO:0005634	nucleus	134	*PCNA*	-0.78	0.057	5.52E+01	-4.84
GOTERM_CC	GO:0005634	nucleus	134	*DNAJB5*	-1.07	0.057	5.52E+01	-4.84

GOTERM_CC	GO:0005634	nucleus	134	*ISX*	0.88	0.057	5.52E+01	-4.84
GOTERM_CC	GO:0005634	nucleus	134	*KIF22*	-0.55	0.057	5.52E+01	-4.84
GOTERM_CC	GO:0005634	nucleus	134	*HNF1A*	0.50	0.057	5.52E+01	-4.84
GOTERM_CC	GO:0005634	nucleus	134	*BACH2*	-2.06	0.057	5.52E+01	-4.84
GOTERM_CC	GO:0005634	nucleus	134	*FAM175A*	-0.68	0.057	5.52E+01	-4.84
GOTERM_CC	GO:0005634	nucleus	134	*WASF1*	-1.20	0.057	5.52E+01	-4.84
GOTERM_CC	GO:0005634	nucleus	134	*E2F8*	-0.71	0.057	5.52E+01	-4.84
GOTERM_CC	GO:0005634	nucleus	134	*NAP1L1*	-0.55	0.057	5.52E+01	-4.84
GOTERM_CC	GO:0005634	nucleus	134	*PAX5*	-4.82	0.057	5.52E+01	-4.84
GOTERM_CC	GO:0005634	nucleus	134	*CCNE2*	-0.74	0.057	5.52E+01	-4.84
GOTERM_CC	GO:0005634	nucleus	134	*FOS*	1.03	0.057	5.52E+01	-4.84
GOTERM_CC	GO:0005634	nucleus	134	*MCM8*	-0.61	0.057	5.52E+01	-4.84
GOTERM_CC	GO:0005634	nucleus	134	*CDIP1*	1.14	0.057	5.52E+01	-4.84
GOTERM_CC	GO:0005634	nucleus	134	*MASTL*	-0.60	0.057	5.52E+01	-4.84
GOTERM_CC	GO:0005634	nucleus	134	*FAM83G*	0.59	0.057	5.52E+01	-4.84
GOTERM_CC	GO:0005634	nucleus	134	*TOP2A*	-0.76	0.057	5.52E+01	-4.84
GOTERM_CC	GO:0005634	nucleus	134	*EGR1*	0.74	0.057	5.52E+01	-4.84
GOTERM_CC	GO:0005634	nucleus	134	*ZFP36*	0.56	0.057	5.52E+01	-4.84
GOTERM_CC	GO:0005634	nucleus	134	*CDK1*	-0.54	0.057	5.52E+01	-4.84
GOTERM_CC	GO:0005634	nucleus	134	*SGK1*	0.54	0.057	5.52E+01	-4.84
GOTERM_CC	GO:0005634	nucleus	134	*GZMA*	0.69	0.057	5.52E+01	-4.84
GOTERM_CC	GO:0005634	nucleus	134	*ANP32E*	-0.64	0.057	5.52E+01	-4.84
GOTERM_CC	GO:0005634	nucleus	134	*TPX2*	-0.71	0.057	5.52E+01	-4.84
GOTERM_CC	GO:0005634	nucleus	134	*TLE4*	0.64	0.057	5.52E+01	-4.84
GOTERM_CC	GO:0005634	nucleus	134	*FAM76B*	-0.60	0.057	5.52E+01	-4.84

GOTERM_CC	GO:0005634	nucleus	134	*MCM3*	-0.67	0.057	5.52E+01	-4.84
GOTERM_CC	GO:0005634	nucleus	134	*UBE2C*	-0.69	0.057	5.52E+01	-4.84
GOTERM_CC	GO:0005634	nucleus	134	*ECT2*	-0.53	0.057	5.52E+01	-4.84
GOTERM_CC	GO:0005634	nucleus	134	*LOC504599*	-0.79	0.057	5.52E+01	-4.84
GOTERM_CC	GO:0005634	nucleus	134	*MCM5*	-0.51	0.057	5.52E+01	-4.84
GOTERM_CC	GO:0005634	nucleus	134	*MCM6*	-0.70	0.057	5.52E+01	-4.84
GOTERM_CC	GO:0005634	nucleus	134	*LPIN3*	0.53	0.057	5.52E+01	-4.84
GOTERM_CC	GO:0005634	nucleus	134	*LARP6*	0.78	0.057	5.52E+01	-4.84
GOTERM_CC	GO:0005634	nucleus	134	*CTH*	-1.03	0.057	5.52E+01	-4.84
GOTERM_CC	GO:0005634	nucleus	134	*FANCD2*	-0.91	0.057	5.52E+01	-4.84
GOTERM_CC	GO:0005634	nucleus	134	*ZNF135*	0.60	0.057	5.52E+01	-4.84
GOTERM_CC	GO:0005634	nucleus	134	*RRM2*	-0.64	0.057	5.52E+01	-4.84
GOTERM_CC	GO:0005634	nucleus	134	*TXN*	-0.56	0.057	5.52E+01	-4.84
GOTERM_CC	GO:0005634	nucleus	134	*MYRF*	0.67	0.057	5.52E+01	-4.84
GOTERM_CC	GO:0005634	nucleus	134	*THOC7*	-0.54	0.057	5.52E+01	-4.84
GOTERM_CC	GO:0005634	nucleus	134	*PRKD3*	-0.61	0.057	5.52E+01	-4.84
GOTERM_CC	GO:0005634	nucleus	134	*MELK*	-0.69	0.057	5.52E+01	-4.84
GOTERM_CC	GO:0005634	nucleus	134	*HIST1H2AC*	-0.59	0.057	5.52E+01	-4.84
GOTERM_CC	GO:0005634	nucleus	134	*DCK*	-0.74	0.057	5.52E+01	-4.84
GOTERM_CC	GO:0005634	nucleus	134	*KIAA0101*	-0.78	0.057	5.52E+01	-4.84
GOTERM_CC	GO:0005634	nucleus	134	*HAT1*	-0.53	0.057	5.52E+01	-4.84
GOTERM_CC	GO:0005634	nucleus	134	*SUMO2*	-0.57	0.057	5.52E+01	-4.84
GOTERM_CC	GO:0005634	nucleus	134	*PLIN2*	0.64	0.057	5.52E+01	-4.84
GOTERM_CC	GO:0005634	nucleus	134	*NR1D1*	0.79	0.057	5.52E+01	-4.84
GOTERM_CC	GO:0005634	nucleus	134	*TCEA1*	-0.73	0.057	5.52E+01	-4.84

GOTERM_CC	GO:0005634	nucleus	134	*APEX1*	-0.50	0.057	5.52E+01	-4.84
GOTERM_CC	GO:0005634	nucleus	134	*MAF*	0.66	0.057	5.52E+01	-4.84
GOTERM_CC	GO:0005634	nucleus	134	*CENPN*	-0.62	0.057	5.52E+01	-4.84
GOTERM_CC	GO:0005634	nucleus	134	*CENPM*	-0.95	0.057	5.52E+01	-4.84
GOTERM_CC	GO:0005634	nucleus	134	*SWAP70*	-0.91	0.057	5.52E+01	-4.84
GOTERM_CC	GO:0005634	nucleus	134	*DLGAP5*	-0.90	0.057	5.52E+01	-4.84
GOTERM_CC	GO:0005634	nucleus	134	*NASP*	-0.51	0.057	5.52E+01	-4.84
GOTERM_CC	GO:0005634	nucleus	134	*KIF18A*	-0.69	0.057	5.52E+01	-4.84
GOTERM_CC	GO:0005634	nucleus	134	*CENPE*	-0.75	0.057	5.52E+01	-4.84
GOTERM_CC	GO:0005634	nucleus	134	*KLK1*	0.80	0.057	5.52E+01	-4.84
GOTERM_CC	GO:0005634	nucleus	134	*CDKN3*	-0.60	0.057	5.52E+01	-4.84
GOTERM_CC	GO:0005634	nucleus	134	*CENPK*	-0.72	0.057	5.52E+01	-4.84
GOTERM_CC	GO:0005634	nucleus	134	*ANXA4*	0.94	0.057	5.52E+01	-4.84
GOTERM_CC	GO:0005634	nucleus	134	*PNLDC1*	-0.76	0.057	5.52E+01	-4.84
GOTERM_CC	GO:0005634	nucleus	134	*LOC616819*	-0.75	0.057	5.52E+01	-4.84
GOTERM_CC	GO:0005634	nucleus	134	*RGS13*	-3.51	0.057	5.52E+01	-4.84
GOTERM_CC	GO:0005634	nucleus	134	*CCNB2*	-0.73	0.057	5.52E+01	-4.84
GOTERM_CC	GO:0005634	nucleus	134	*HIST1H2AJ*	-1.05	0.057	5.52E+01	-4.84
GOTERM_CC	GO:0005634	nucleus	134	*PBX4*	0.61	0.057	5.52E+01	-4.84
GOTERM_BP	GO:0051301	cell division	24	*CDC6*	-0.76	0.000	6.37E-05	-4.90
GOTERM_BP	GO:0051301	cell division	24	*CDK1*	-0.54	0.000	6.37E-05	-4.90
GOTERM_BP	GO:0051301	cell division	24	*TPX2*	-0.71	0.000	6.37E-05	-4.90
GOTERM_BP	GO:0051301	cell division	24	*SPDL1*	-0.78	0.000	6.37E-05	-4.90
GOTERM_BP	GO:0051301	cell division	24	*AURKA*	-0.52	0.000	6.37E-05	-4.90
GOTERM_BP	GO:0051301	cell division	24	*UBE2C*	-0.69	0.000	6.37E-05	-4.90

GOTERM_BP	GO:0051301	cell division	24	*CCNB1*	-0.73	0.000	6.37E-05	-4.90
GOTERM_BP	GO:0051301	cell division	24	*CCNE2*	-0.74	0.000	6.37E-05	-4.90
GOTERM_BP	GO:0051301	cell division	24	*SPC24*	-0.56	0.000	6.37E-05	-4.90
GOTERM_BP	GO:0051301	cell division	24	*FAM83D*	-0.51	0.000	6.37E-05	-4.90
GOTERM_BP	GO:0051301	cell division	24	*SPC25*	-0.62	0.000	6.37E-05	-4.90
GOTERM_BP	GO:0051301	cell division	24	*VRK1*	-0.58	0.000	6.37E-05	-4.90
GOTERM_BP	GO:0051301	cell division	24	*CCNB2*	-0.73	0.000	6.37E-05	-4.90
GOTERM_BP	GO:0051301	cell division	24	*APITD1*	-0.76	0.000	6.37E-05	-4.90
GOTERM_BP	GO:0051301	cell division	24	*CDCA2*	-0.59	0.000	6.37E-05	-4.90
GOTERM_BP	GO:0051301	cell division	24	*CKS2*	-0.50	0.000	6.37E-05	-4.90
GOTERM_BP	GO:0051301	cell division	24	*SKA2*	-0.67	0.000	6.37E-05	-4.90
GOTERM_BP	GO:0051301	cell division	24	*MASTL*	-0.60	0.000	6.37E-05	-4.90
GOTERM_BP	GO:0051301	cell division	24	*SKA1*	-0.81	0.000	6.37E-05	-4.90
GOTERM_BP	GO:0051301	cell division	24	*ZWILCH*	-0.65	0.000	6.37E-05	-4.90
GOTERM_BP	GO:0051301	cell division	24	*CCNA2*	-0.67	0.000	6.37E-05	-4.90
GOTERM_BP	GO:0051301	cell division	24	*ASPM*	-0.68	0.000	6.37E-05	-4.90
GOTERM_BP	GO:0051301	cell division	24	*CDCA3*	-0.64	0.000	6.37E-05	-4.90
GOTERM_BP	GO:0051301	cell division	24	*ERCC6L*	-0.78	0.000	6.37E-05	-4.90
GOTERM_CC	GO:0005737	cytoplasm	144	*PRC1*	-0.54	0.071	6.34E+01	-5.00
GOTERM_CC	GO:0005737	cytoplasm	144	*CNDP2*	0.68	0.071	6.34E+01	-5.00
GOTERM_CC	GO:0005737	cytoplasm	144	*KNTC1*	-0.74	0.071	6.34E+01	-5.00
GOTERM_CC	GO:0005737	cytoplasm	144	*PKMYT1*	-0.76	0.071	6.34E+01	-5.00
GOTERM_CC	GO:0005737	cytoplasm	144	*AURKA*	-0.52	0.071	6.34E+01	-5.00
GOTERM_CC	GO:0005737	cytoplasm	144	*AURKB*	-0.69	0.071	6.34E+01	-5.00
GOTERM_CC	GO:0005737	cytoplasm	144	*MCM10*	-0.76	0.071	6.34E+01	-5.00

GOTERM_CC	GO:0005737	cytoplasm	144	*AQP3*	0.94	0.071	6.34E+01	-5.00
GOTERM_CC	GO:0005737	cytoplasm	144	*LNX1*	1.01	0.071	6.34E+01	-5.00
GOTERM_CC	GO:0005737	cytoplasm	144	*ACTG2*	-1.39	0.071	6.34E+01	-5.00
GOTERM_CC	GO:0005737	cytoplasm	144	*CDCA7*	-0.59	0.071	6.34E+01	-5.00
GOTERM_CC	GO:0005737	cytoplasm	144	*HIST1H2BN*	-0.72	0.071	6.34E+01	-5.00
GOTERM_CC	GO:0005737	cytoplasm	144	*MCOLN3*	-1.66	0.071	6.34E+01	-5.00
GOTERM_CC	GO:0005737	cytoplasm	144	*CDKN2C*	-0.65	0.071	6.34E+01	-5.00
GOTERM_CC	GO:0005737	cytoplasm	144	*ROBO1*	-0.54	0.071	6.34E+01	-5.00
GOTERM_CC	GO:0005737	cytoplasm	144	*OIP5*	-0.69	0.071	6.34E+01	-5.00
GOTERM_CC	GO:0005737	cytoplasm	144	*RARA*	0.54	0.071	6.34E+01	-5.00
GOTERM_CC	GO:0005737	cytoplasm	144	*NQO1*	-0.78	0.071	6.34E+01	-5.00
GOTERM_CC	GO:0005737	cytoplasm	144	*MX1*	0.67	0.071	6.34E+01	-5.00
GOTERM_CC	GO:0005737	cytoplasm	144	*CCNA2*	-0.67	0.071	6.34E+01	-5.00
GOTERM_CC	GO:0005737	cytoplasm	144	*ORC1*	-0.89	0.071	6.34E+01	-5.00
GOTERM_CC	GO:0005737	cytoplasm	144	*ASPM*	-0.68	0.071	6.34E+01	-5.00
GOTERM_CC	GO:0005737	cytoplasm	144	*DTL*	-0.53	0.071	6.34E+01	-5.00
GOTERM_CC	GO:0005737	cytoplasm	144	*DYX1C1*	-0.89	0.071	6.34E+01	-5.00
GOTERM_CC	GO:0005737	cytoplasm	144	*FBP1*	1.19	0.071	6.34E+01	-5.00
GOTERM_CC	GO:0005737	cytoplasm	144	*RMI2*	-1.46	0.071	6.34E+01	-5.00
GOTERM_CC	GO:0005737	cytoplasm	144	*BASP1*	-0.53	0.071	6.34E+01	-5.00
GOTERM_CC	GO:0005737	cytoplasm	144	*METTL7A*	-1.07	0.071	6.34E+01	-5.00
GOTERM_CC	GO:0005737	cytoplasm	144	*PKIA*	-1.54	0.071	6.34E+01	-5.00
GOTERM_CC	GO:0005737	cytoplasm	144	*MOXD1*	-1.70	0.071	6.34E+01	-5.00
GOTERM_CC	GO:0005737	cytoplasm	144	*NME2*	-0.53	0.071	6.34E+01	-5.00
GOTERM_CC	GO:0005737	cytoplasm	144	*TAGLN*	-0.82	0.071	6.34E+01	-5.00

GOTERM_CC	GO:0005737	cytoplasm	144	*HSPB6*	-1.08	0.071	6.34E+01	-5.00
GOTERM_CC	GO:0005737	cytoplasm	144	*SPAG5*	-0.73	0.071	6.34E+01	-5.00
GOTERM_CC	GO:0005737	cytoplasm	144	*CDCA7L*	-0.52	0.071	6.34E+01	-5.00
GOTERM_CC	GO:0005737	cytoplasm	144	*STMN1*	-1.40	0.071	6.34E+01	-5.00
GOTERM_CC	GO:0005737	cytoplasm	144	*NFE2L3*	-0.57	0.071	6.34E+01	-5.00
GOTERM_CC	GO:0005737	cytoplasm	144	*ASB4*	1.92	0.071	6.34E+01	-5.00
GOTERM_CC	GO:0005737	cytoplasm	144	*SH3GL2*	0.95	0.071	6.34E+01	-5.00
GOTERM_CC	GO:0005737	cytoplasm	144	*GRB14*	-1.69	0.071	6.34E+01	-5.00
GOTERM_CC	GO:0005737	cytoplasm	144	*CDK5R1*	0.72	0.071	6.34E+01	-5.00
GOTERM_CC	GO:0005737	cytoplasm	144	*HMGB2*	-0.59	0.071	6.34E+01	-5.00
GOTERM_CC	GO:0005737	cytoplasm	144	*ALDOB*	0.68	0.071	6.34E+01	-5.00
GOTERM_CC	GO:0005737	cytoplasm	144	*SESN2*	0.65	0.071	6.34E+01	-5.00
GOTERM_CC	GO:0005737	cytoplasm	144	*VRK1*	-0.58	0.071	6.34E+01	-5.00
GOTERM_CC	GO:0005737	cytoplasm	144	*ISYNA1*	0.61	0.071	6.34E+01	-5.00
GOTERM_CC	GO:0005737	cytoplasm	144	*SKA2*	-0.67	0.071	6.34E+01	-5.00
GOTERM_CC	GO:0005737	cytoplasm	144	*SKA1*	-0.81	0.071	6.34E+01	-5.00
GOTERM_CC	GO:0005737	cytoplasm	144	*AGRN*	0.59	0.071	6.34E+01	-5.00
GOTERM_CC	GO:0005737	cytoplasm	144	*LOC505183*	-0.88	0.071	6.34E+01	-5.00
GOTERM_CC	GO:0005737	cytoplasm	144	*ERCC6L*	-0.78	0.071	6.34E+01	-5.00
GOTERM_CC	GO:0005737	cytoplasm	144	*CRIP1*	0.57	0.071	6.34E+01	-5.00
GOTERM_CC	GO:0005737	cytoplasm	144	*LOC537017*	-0.79	0.071	6.34E+01	-5.00
GOTERM_CC	GO:0005737	cytoplasm	144	*SPTBN5*	0.69	0.071	6.34E+01	-5.00
GOTERM_CC	GO:0005737	cytoplasm	144	*BRIP1*	-0.73	0.071	6.34E+01	-5.00
GOTERM_CC	GO:0005737	cytoplasm	144	*NR4A1*	1.03	0.071	6.34E+01	-5.00
GOTERM_CC	GO:0005737	cytoplasm	144	*SPDL1*	-0.78	0.071	6.34E+01	-5.00

GOTERM_CC	GO:0005737	cytoplasm	144	*FOXP3*	1.07	0.071	6.34E+01	-5.00
GOTERM_CC	GO:0005737	cytoplasm	144	*HOMER2*	-1.03	0.071	6.34E+01	-5.00
GOTERM_CC	GO:0005737	cytoplasm	144	*BRCA1*	-0.56	0.071	6.34E+01	-5.00
GOTERM_CC	GO:0005737	cytoplasm	144	*FAM64A*	-0.75	0.071	6.34E+01	-5.00
GOTERM_CC	GO:0005737	cytoplasm	144	*PARPBP*	-0.59	0.071	6.34E+01	-5.00
GOTERM_CC	GO:0005737	cytoplasm	144	*PCNA*	-0.78	0.071	6.34E+01	-5.00
GOTERM_CC	GO:0005737	cytoplasm	144	*CD79B*	-3.11	0.071	6.34E+01	-5.00
GOTERM_CC	GO:0005737	cytoplasm	144	*PLA2G4B*	0.55	0.071	6.34E+01	-5.00
GOTERM_CC	GO:0005737	cytoplasm	144	*MYLK*	-0.86	0.071	6.34E+01	-5.00
GOTERM_CC	GO:0005737	cytoplasm	144	*SNRNP25*	-0.56	0.071	6.34E+01	-5.00
GOTERM_CC	GO:0005737	cytoplasm	144	*DUT*	-0.65	0.071	6.34E+01	-5.00
GOTERM_CC	GO:0005737	cytoplasm	144	*CLSPN*	-0.73	0.071	6.34E+01	-5.00
GOTERM_CC	GO:0005737	cytoplasm	144	*KIF22*	-0.55	0.071	6.34E+01	-5.00
GOTERM_CC	GO:0005737	cytoplasm	144	*DLEC1*	1.24	0.071	6.34E+01	-5.00
GOTERM_CC	GO:0005737	cytoplasm	144	*HNF1A*	0.50	0.071	6.34E+01	-5.00
GOTERM_CC	GO:0005737	cytoplasm	144	*KCNAB2*	0.51	0.071	6.34E+01	-5.00
GOTERM_CC	GO:0005737	cytoplasm	144	*WASF1*	-1.20	0.071	6.34E+01	-5.00
GOTERM_CC	GO:0005737	cytoplasm	144	*SH2D4A*	0.76	0.071	6.34E+01	-5.00
GOTERM_CC	GO:0005737	cytoplasm	144	*FAM83D*	-0.51	0.071	6.34E+01	-5.00
GOTERM_CC	GO:0005737	cytoplasm	144	*MASTL*	-0.60	0.071	6.34E+01	-5.00
GOTERM_CC	GO:0005737	cytoplasm	144	*FBXL15*	0.52	0.071	6.34E+01	-5.00
GOTERM_CC	GO:0005737	cytoplasm	144	*PPP1R14A*	-0.60	0.071	6.34E+01	-5.00
GOTERM_CC	GO:0005737	cytoplasm	144	*TUBA1C*	0.53	0.071	6.34E+01	-5.00
GOTERM_CC	GO:0005737	cytoplasm	144	*ALS2CL*	0.53	0.071	6.34E+01	-5.00
GOTERM_CC	GO:0005737	cytoplasm	144	*EGR1*	0.74	0.071	6.34E+01	-5.00

GOTERM_CC	GO:0005737	cytoplasm	144	*ZFP36*	0.56	0.071	6.34E+01	-5.00
GOTERM_CC	GO:0005737	cytoplasm	144	*SGK1*	0.54	0.071	6.34E+01	-5.00
GOTERM_CC	GO:0005737	cytoplasm	144	*KIF11*	-0.94	0.071	6.34E+01	-5.00
GOTERM_CC	GO:0005737	cytoplasm	144	*TPX2*	-0.71	0.071	6.34E+01	-5.00
GOTERM_CC	GO:0005737	cytoplasm	144	*NUSAP1*	-0.88	0.071	6.34E+01	-5.00
GOTERM_CC	GO:0005737	cytoplasm	144	*UBE2C*	-0.69	0.071	6.34E+01	-5.00
GOTERM_CC	GO:0005737	cytoplasm	144	*ELMO3*	0.52	0.071	6.34E+01	-5.00
GOTERM_CC	GO:0005737	cytoplasm	144	*ECT2*	-0.53	0.071	6.34E+01	-5.00
GOTERM_CC	GO:0005737	cytoplasm	144	*TARS*	-1.19	0.071	6.34E+01	-5.00
GOTERM_CC	GO:0005737	cytoplasm	144	*CTH*	-1.03	0.071	6.34E+01	-5.00
GOTERM_CC	GO:0005737	cytoplasm	144	*CAPN11*	0.75	0.071	6.34E+01	-5.00
GOTERM_CC	GO:0005737	cytoplasm	144	*FANCD2*	-0.91	0.071	6.34E+01	-5.00
GOTERM_CC	GO:0005737	cytoplasm	144	*DACT3*	-0.69	0.071	6.34E+01	-5.00
GOTERM_CC	GO:0005737	cytoplasm	144	*CKAP2L*	-0.55	0.071	6.34E+01	-5.00
GOTERM_CC	GO:0005737	cytoplasm	144	*TXN*	-0.56	0.071	6.34E+01	-5.00
GOTERM_CC	GO:0005737	cytoplasm	144	*THOC7*	-0.54	0.071	6.34E+01	-5.00
GOTERM_CC	GO:0005737	cytoplasm	144	*FCRLA*	-5.11	0.071	6.34E+01	-5.00
GOTERM_CC	GO:0005737	cytoplasm	144	*LOC787465*	-0.71	0.071	6.34E+01	-5.00
GOTERM_CC	GO:0005737	cytoplasm	144	*PRKD3*	-0.61	0.071	6.34E+01	-5.00
GOTERM_CC	GO:0005737	cytoplasm	144	*MELK*	-0.69	0.071	6.34E+01	-5.00
GOTERM_CC	GO:0005737	cytoplasm	144	*ABTB1*	0.53	0.071	6.34E+01	-5.00
GOTERM_CC	GO:0005737	cytoplasm	144	*TKTL2*	-1.43	0.071	6.34E+01	-5.00
GOTERM_CC	GO:0005737	cytoplasm	144	*TPM2*	-1.04	0.071	6.34E+01	-5.00
GOTERM_CC	GO:0005737	cytoplasm	144	*TPM1*	-0.86	0.071	6.34E+01	-5.00
GOTERM_CC	GO:0005737	cytoplasm	144	*HIC1*	0.68	0.071	6.34E+01	-5.00

GOTERM_CC	GO:0005737	cytoplasm	144	*NDC1*	-0.79	0.071	6.34E+01	-5.00
GOTERM_CC	GO:0005737	cytoplasm	144	*CCT6B*	-0.66	0.071	6.34E+01	-5.00
GOTERM_CC	GO:0005737	cytoplasm	144	*NR1D1*	0.79	0.071	6.34E+01	-5.00
GOTERM_CC	GO:0005737	cytoplasm	144	*NCAPG*	-0.74	0.071	6.34E+01	-5.00
GOTERM_CC	GO:0005737	cytoplasm	144	*RASGRP4*	0.52	0.071	6.34E+01	-5.00
GOTERM_CC	GO:0005737	cytoplasm	144	*SAPCD2*	0.66	0.071	6.34E+01	-5.00
GOTERM_CC	GO:0005737	cytoplasm	144	*BUB1*	-0.71	0.071	6.34E+01	-5.00
GOTERM_CC	GO:0005737	cytoplasm	144	*GSTO1*	-1.12	0.071	6.34E+01	-5.00
GOTERM_CC	GO:0005737	cytoplasm	144	*APEX1*	-0.50	0.071	6.34E+01	-5.00
GOTERM_CC	GO:0005737	cytoplasm	144	*MAF*	0.66	0.071	6.34E+01	-5.00
GOTERM_CC	GO:0005737	cytoplasm	144	*EXO1*	-0.70	0.071	6.34E+01	-5.00
GOTERM_CC	GO:0005737	cytoplasm	144	*FLRT2*	-0.52	0.071	6.34E+01	-5.00
GOTERM_CC	GO:0005737	cytoplasm	144	*CENPM*	-0.95	0.071	6.34E+01	-5.00
GOTERM_CC	GO:0005737	cytoplasm	144	*HIST1H2BD*	-0.59	0.071	6.34E+01	-5.00
GOTERM_CC	GO:0005737	cytoplasm	144	*SWAP70*	-0.91	0.071	6.34E+01	-5.00
GOTERM_CC	GO:0005737	cytoplasm	144	*DLGAP5*	-0.90	0.071	6.34E+01	-5.00
GOTERM_CC	GO:0005737	cytoplasm	144	*NASP*	-0.51	0.071	6.34E+01	-5.00
GOTERM_CC	GO:0005737	cytoplasm	144	*KIF18A*	-0.69	0.071	6.34E+01	-5.00
GOTERM_CC	GO:0005737	cytoplasm	144	*CENPF*	-0.82	0.071	6.34E+01	-5.00
GOTERM_CC	GO:0005737	cytoplasm	144	*RCAN1*	-0.55	0.071	6.34E+01	-5.00
GOTERM_CC	GO:0005737	cytoplasm	144	*CENPE*	-0.75	0.071	6.34E+01	-5.00
GOTERM_CC	GO:0005737	cytoplasm	144	*CDKN3*	-0.60	0.071	6.34E+01	-5.00
GOTERM_CC	GO:0005737	cytoplasm	144	*SMC2*	-0.71	0.071	6.34E+01	-5.00
GOTERM_CC	GO:0005737	cytoplasm	144	*CISH*	0.52	0.071	6.34E+01	-5.00
GOTERM_CC	GO:0005737	cytoplasm	144	*RGS13*	-3.51	0.071	6.34E+01	-5.00

GOTERM_CC	GO:0005737	cytoplasm	144	*SMC4*	-0.58	0.071	6.34E+01	-5.00
GOTERM_CC	GO:0005737	cytoplasm	144	*PCK1*	3.19	0.071	6.34E+01	-5.00
GOTERM_CC	GO:0005737	cytoplasm	144	*CCNB1*	-0.73	0.071	6.34E+01	-5.00
GOTERM_CC	GO:0005737	cytoplasm	144	*MICALCL*	0.62	0.071	6.34E+01	-5.00
GOTERM_CC	GO:0005737	cytoplasm	144	*PYGM*	-1.11	0.071	6.34E+01	-5.00
GOTERM_CC	GO:0005737	cytoplasm	144	*IRF7*	0.54	0.071	6.34E+01	-5.00
GOTERM_CC	GO:0005737	cytoplasm	144	*KCNN3*	-0.90	0.071	6.34E+01	-5.00
GOTERM_CC	GO:0005737	cytoplasm	144	*CHN1*	-0.97	0.071	6.34E+01	-5.00
GOTERM_CC	GO:0005737	cytoplasm	144	*ACE2*	1.12	0.071	6.34E+01	-5.00
GOTERM_CC	GO:0005737	cytoplasm	144	*GPT*	0.97	0.071	6.34E+01	-5.00
GOTERM_CC	GO:0005737	cytoplasm	144	*PSAT1*	-1.22	0.071	6.34E+01	-5.00
GOTERM_CC	GO:0005737	cytoplasm	144	*BARD1*	-0.68	0.071	6.34E+01	-5.00
GOTERM_MF	GO:0003677	DNA binding	43	*HIST1H2AC*	-0.59	0.001	8.64E-01	-5.03
GOTERM_MF	GO:0003677	DNA binding	43	*KIF22*	-0.55	0.001	8.64E-01	-5.03
GOTERM_MF	GO:0003677	DNA binding	43	*HIST1H2AG*	-0.84	0.001	8.64E-01	-5.03
GOTERM_MF	GO:0003677	DNA binding	43	*DCK*	-0.74	0.001	8.64E-01	-5.03
GOTERM_MF	GO:0003677	DNA binding	43	*CERS4*	-1.50	0.001	8.64E-01	-5.03
GOTERM_MF	GO:0003677	DNA binding	43	*FOS*	1.03	0.001	8.64E-01	-5.03
GOTERM_MF	GO:0003677	DNA binding	43	*HIST1H2BM*	-0.53	0.001	8.64E-01	-5.03
GOTERM_MF	GO:0003677	DNA binding	43	*MCM8*	-0.61	0.001	8.64E-01	-5.03
GOTERM_MF	GO:0003677	DNA binding	43	*HIST1H2BN*	-0.72	0.001	8.64E-01	-5.03
GOTERM_MF	GO:0003677	DNA binding	43	*WDR76*	-1.21	0.001	8.64E-01	-5.03
GOTERM_MF	GO:0003677	DNA binding	43	*H2AFZ*	-0.60	0.001	8.64E-01	-5.03
GOTERM_MF	GO:0003677	DNA binding	43	*TCEA1*	-0.73	0.001	8.64E-01	-5.03
GOTERM_MF	GO:0003677	DNA binding	43	*LOC505183*	-0.88	0.001	8.64E-01	-5.03

GOTERM_MF	GO:0003677	DNA binding	43	*ORC1*	-0.89	0.001	8.64E-01	-5.03
GOTERM_MF	GO:0003677	DNA binding	43	*APEX1*	-0.50	0.001	8.64E-01	-5.03
GOTERM_MF	GO:0003677	DNA binding	43	*FEN1*	-0.55	0.001	8.64E-01	-5.03
GOTERM_MF	GO:0003677	DNA binding	43	*ERCC6L*	-0.78	0.001	8.64E-01	-5.03
GOTERM_MF	GO:0003677	DNA binding	43	*EXO1*	-0.70	0.001	8.64E-01	-5.03
GOTERM_MF	GO:0003677	DNA binding	43	*ZFP36*	0.56	0.001	8.64E-01	-5.03
GOTERM_MF	GO:0003677	DNA binding	43	*HIST1H2BB*	-0.64	0.001	8.64E-01	-5.03
GOTERM_MF	GO:0003677	DNA binding	43	*POU2AF1*	-1.71	0.001	8.64E-01	-5.03
GOTERM_MF	GO:0003677	DNA binding	43	*HIST1H1E*	-0.54	0.001	8.64E-01	-5.03
GOTERM_MF	GO:0003677	DNA binding	43	*CR2*	-3.53	0.001	8.64E-01	-5.03
GOTERM_MF	GO:0003677	DNA binding	43	*HIST1H2BD*	-0.59	0.001	8.64E-01	-5.03
GOTERM_MF	GO:0003677	DNA binding	43	*GEN1*	-0.78	0.001	8.64E-01	-5.03
GOTERM_MF	GO:0003677	DNA binding	43	*SWAP70*	-0.91	0.001	8.64E-01	-5.03
GOTERM_MF	GO:0003677	DNA binding	43	*POLE*	-0.66	0.001	8.64E-01	-5.03
GOTERM_MF	GO:0003677	DNA binding	43	*BRIP1*	-0.73	0.001	8.64E-01	-5.03
GOTERM_MF	GO:0003677	DNA binding	43	*NR4A1*	1.03	0.001	8.64E-01	-5.03
GOTERM_MF	GO:0003677	DNA binding	43	*NUSAP1*	-0.88	0.001	8.64E-01	-5.03
GOTERM_MF	GO:0003677	DNA binding	43	*RMI2*	-1.46	0.001	8.64E-01	-5.03
GOTERM_MF	GO:0003677	DNA binding	43	*MCM3*	-0.67	0.001	8.64E-01	-5.03
GOTERM_MF	GO:0003677	DNA binding	43	*BRCA1*	-0.56	0.001	8.64E-01	-5.03
GOTERM_MF	GO:0003677	DNA binding	43	*MCM6*	-0.70	0.001	8.64E-01	-5.03
GOTERM_MF	GO:0003677	DNA binding	43	*APITD1*	-0.76	0.001	8.64E-01	-5.03
GOTERM_MF	GO:0003677	DNA binding	43	*ZNF135*	0.60	0.001	8.64E-01	-5.03
GOTERM_MF	GO:0003677	DNA binding	43	*PARPBP*	-0.59	0.001	8.64E-01	-5.03
GOTERM_MF	GO:0003677	DNA binding	43	*MYRF*	0.67	0.001	8.64E-01	-5.03

GOTERM_MF	GO:0003677	DNA binding	43	*CENPW*	-1.27	0.001	8.64E-01	-5.03
GOTERM_MF	GO:0003677	DNA binding	43	*RAD18*	-0.58	0.001	8.64E-01	-5.03
GOTERM_MF	GO:0003677	DNA binding	43	*MIS18BP1*	-0.67	0.001	8.64E-01	-5.03
GOTERM_MF	GO:0003677	DNA binding	43	*HIST1H2AJ*	-1.05	0.001	8.64E-01	-5.03
GOTERM_MF	GO:0003677	DNA binding	43	*LOC787465*	-0.71	0.001	8.64E-01	-5.03
