# Supplementary material for: Analysis of Host Jejunum Transcriptome and Associated Microbial Community Structure Variation in Young Calves with Feed-Induced Acidosis
Source: Metabolites. 2021 Jun 23;11(7):414. doi: 10.3390/metabo11070414 (PMC8303401; doi:10.3390/metabo11070414)
Supplement: Supplementary file 1 [file metabolites-11-00414-s001.zip › Supplemental Table S3-Function-SLC.pdf]

Supplemental Table S3 Functional annotation for differentially expressed SLC protein-coding genes between Aci and Con groups

Category	Term	Count	*P*-value	Genes
Biological process	GO:0035879~plasma membrane lactate transport	4	< 0.001	SLC16A3, SLC16A5, SLC16A1, SLC16A7
	GO:0035725~sodium ion transmembrane transport	3	0.001	SLC6A8, SLC24A1, SLC4A4
	GO:0015804~neutral amino acid transport	2	0.010	SLC7A8, SLC43A2
	GO:0089711~L-glutamate transmembrane transport	2	0.010	SLC25A22, SLC1A1
	GO:0006836~neurotransmitter transport	2	0.027	SLC6A8, SLC6A12
	GO:0015701~bicarbonate transport	2	0.028	SLC26A6, SLC4A4
	GO:0006412~translation	3	0.030	SLC25A20, SLC25A34, SLC25A22
	GO:0006814~sodium ion transport	2	0.045	SLC9B2, SLC4A4
Cellular constituent	GO:0005887~integral component of plasma membrane	16	< 0.001	SLCO4A1, SLC6A12, SLC7A8, SLC7A10, SLC7A7, SLC16A3, SLC16A5, SLC26A6, SLC16A1, SLC6A7, SLC16A7, SLC6A8, SLC24A1, SLC22A5, SLC4A4, SLC43A2
	GO:0016021~integral component of membrane	15	0.002	SLC15A1, SLC22A15, SLC6A12, SLC3A2, SLC7A10, SLC7A7, SLC25A20, SLC25A34, SLC25A22, SLC9B2, SLC22A5, SLC4A4, SLC51A, SLC1A1, SLC31A1
	GO:0016324~apical plasma membrane	3	0.028	SLC26A6, SLC22A5, SLC1A1
	GO:0031526~brush border membrane	2	0.041	SLC26A6, SLC22A5
Molecular function	GO:0008028~monocarboxylic acid transmembrane transporter activity	4	< 0.001	SLC16A3, SLC16A5, SLC16A1, SLC16A7
	GO:0015129~lactate transmembrane transporter activity	4	< 0.001	SLC16A3, SLC16A5, SLC16A1, SLC16A7
	GO:0015293~symporter activity	4	< 0.001	SLC16A1, SLC25A22, SLC24A1, SLC1A1
	GO:0015179~L-amino acid transmembrane transporter activity	3	< 0.001	SLC7A8, SLC43A2, SLC7A7
	GO:0005328~neurotransmitter:sodium symporter activity	3	< 0.001	SLC6A7, SLC6A8, SLC6A12
	GO:0015297~antiporter activity	3	0.001	SLC7A8, SLC7A10, SLC7A7
	GO:0015175~neutral amino acid transmembrane transporter activity	2	0.011	SLC7A8, SLC43A2
	GO:0005313~L-glutamate transmembrane transporter activity	2	0.015	SLC25A22, SLC1A1
	GO:0015171~amino acid transmembrane transporter activity	2	0.049	SLC7A10, SLC7A7
Pathway	bta04974:Protein digestion and absorption	5	< 0.001	SLC15A1, SLC3A2, SLC7A8, SLC1A1, SLC7A7
