# Supplementary material for: Analysis of Host Jejunum Transcriptome and Associated Microbial Community Structure Variation in Young Calves with Feed-Induced Acidosis
Source: Metabolites. 2021 Jun 23;11(7):414. doi: 10.3390/metabo11070414 (PMC8303401; doi:10.3390/metabo11070414)
Supplement: Supplementary file 1 [file metabolites-11-00414-s001.zip › Supplemental Table S4-Genera.pdf]

Supplemental Table S4 Read counts of genera in the jejunum between Aci and Con groups

N	Genus	Con_mean_RC	Aci_mean_RC
1	*Cutibacterium*	1.75	4
2	*Streptococcus*	14	17
3	*Spirosoma*	0.25	0.5
4	*Nitrosospira*	68.75	14.25
5	*Actinomyces*	2.5	1
6	*Staphylococcus*	31	26.75
7	*Synechococcus*	2	3
8	*Pseudomonas*	28	26.5
9	*Tannerella*	1	1.5
10	*Sphingomonas*	4.5	4.25
11	*Bacteroides*	47.5	32.5
12	*Selenomonas*	95	23
13	*Sphaerochaeta*	3.5	3.5
14	*Dickeya*	40	14.5
15	*Desulfovibrio*	3.75	4.5
16	*Erysipelothrix*	0.25	1
17	*Veillonella*	6	6.25
18	*Treponema*	27	13.5
19	*Porphyromonas*	0.5	0.5
20	*Orthobunyavirus*	0.5	1.25
21	*Francisella*	55	14
22	*Lactobacillus*	33	28.25
23	*Desulfococcus*	0.25	0
24	*Ochrobactrum*	21.75	8.25
25	*Mycobacterium*	2.25	0.25
26	*Bifidobacterium*	13.25	8
27	*Parabacteroides*	23.25	14.5
28	*Streptomyces*	2.25	5.25
29	*Bacillus*	15	5.25
30	*Clostridium*	56.25	61.75
31	*Burkholderia*	0	0.75
32	*Blautia*	25.25	8
33	*Kyrpidia*	3.25	1
34	*Olsenella*	7	12.25
35	*Methylobacterium*	13.75	13
36	*Aeromonas*	1.5	2.25
37	*Ralstonia*	0.25	0
38	*Salmonella*	0.75	1
39	*Methanobrevibacter*	11.5	6.5
40	*Paenibacillus*	4	2.25

41	*Haemophilus*	0.25	0.25
42	*Campylobacter*	13.75	23.5
43	*Aerococcus*	1	1.5
44	*Mucilaginibacter*	3.5	0.5
45	*Mycoplasma*	2.5	4.25
46	*Eubacterium*	2.25	0.75
47	*Psychrobacter*	9.75	6.25
48	*Acinetobacter*	89.75	63.75
49	*Ruminococcus*	24.25	4.5
50	*Lachnoclostridium*	91.75	12.75
51	*Flavobacterium*	12.25	8.5
52	*Verrucomicrobium*	0.5	0.25
53	*Acidaminococcus*	10	13.25
54	*Anaerococcus*	6.5	1.5
55	*Prevotella*	538.5	166.5
56	*Moraxella*	5.75	0.5
57	*Butyrivibrio*	26	6
58	*Paracoccus*	0.75	0.5
59	*Corynebacterium*	16.5	18
60	*Hymenobacter*	1	1.5
